# Declining *Onchocerca volvulus* transmission despite limited ivermectin delivery in the Kakoi–Koda focus, Ituri, Democratic Republic of the Congo: An epidemiological, entomological and landscape evidence synthesis

**DOI:** 10.1371/journal.pntd.0014164

**Published:** 2026-08-31

**Authors:** Luís-Jorge Amaral, Tony Ukety, Jules Upenjirwoth, Deogratias Ucima Wonya’Rossi, Michel Ndahura Mandro, Françoise Nyisi, Pascal Adroba, Wilma A. Stolk, Joseph N. Siewe Fodjo, María-Gloria Basáñez, Anne Laudisoit, Robert Colebunders

**Affiliations:** 1 Global Health Institute, Faculty of Medicine and Health Sciences, University of Antwerp, Antwerpen, Belgium; 2 UK Medical Research Council Centre for Global Infectious Disease Analysis, Department of Infectious Disease Epidemiology, School of Public Health, Imperial College London, London, United Kingdom; 3 Centre de Recherche en Maladies Tropicales (CRMT), Rethy, Democratic Republic of Congo; 4 Provincial Health Division Ituri, Ministry of Health, Bunia, Democratic Republic of Congo; 5 Department of Public Health, Erasmus University Rotterdam, Rotterdam, The Netherlands; 6 Evolutionary Ecology Group, Faculty of Sciences, University of Antwerp, Antwerpen, Belgium; 7 Nature.Health.Global, New York, New York, United States of America; University of Buea, CAMEROON

## Abstract

**Background:**

The Kakoi–Koda focus in Ituri Province was historically hyperendemic for onchocerciasis, yet screening for a recent trial suggested a marked decline, including in Logo Health Zone, which had never received routine ivermectin. We explored whether this decline extended across the focus and how infection indicators corresponded spatially with ivermectin delivery, entomological observations and deforestation.

**Methodology:**

We conducted a scoping evidence synthesis of epidemiological, programmatic, entomological and geospatial sources. Against a 2003 nodule-mapping baseline, change was assessed from repeated cross-sectional moxidectin trial screenings in 2010–11 and 2021–2023, which applied the same four-skin-snip protocol to community-recruited residents aged ≥12 years. Anti-Ov16 serology (2015–21), two-skin-snip surveys (2015/17), exploratory blackfly observations (2009–18) and remotely sensed tree-cover loss (2001–24) provided further contextual evidence.

**Principal findings:**

Between the two trial screenings, microfilarial prevalence significantly declined from 79.0% to 9.0% (Draju) and 68.9% to 8.6% (Kanga) in Logo, similar to declines observed in villages of Nyarambe Health Zone (72.2% to 2.9%), which received routine ivermectin for lymphatic filariasis. Mean infection intensity mirrored this pattern, from 17-26 to 1 microfilariae per milligram of skin in Logo, and 11 to 0.4 in Nyarambe. Seroprevalence in children aged 3–10 years from 2016 onward was low (0–5%), geographically circumscribed and broadly concordant with the skin-snip spatial pattern. Opportunistic blackfly collections and breeding-site prospections detected *Simulium dentulosum* and *S. vorax* as the current anthropophagic species, with no evidence of *S. neavei* after 2009. Extensive dense forest loss (75–90% in historically hyperendemic Logo) and canopy opening are consistent with a shift from crab-associated *S. neavei* habitats towards more open-habitat vectors, providing a plausible ecological mechanism.

**Significance:**

Parasitological, serological, entomological and geospatial evidence consistently indicates substantial declines in *O. volvulus* infection indicators across Kakoi-Koda, compatible with reduced transmission. Residual positive indicators were spatially circumscribed, including in Logo where no routine ivermectin was delivered. Whether the current simuliid species can sustain transmission above elimination thresholds remains uncertain. Standardised, representative surveys in the Muda/Kuda and Lebu River basins are warranted to guide decisions on starting and stopping ivermectin delivery.

## Author summary

Onchocerciasis, also known as river blindness, is a parasitic disease of public health concern in sub-Saharan Africa, transmitted by blackfly vectors. The disease is responsible for skin and eye clinical manifestations and is associated with neurological complications. We investigated an area in north-eastern Democratic Republic of the Congo called the Kakoi-Koda onchocerciasis focus, where the infection was common in the early 2000s. We reviewed and assembled data from past studies on infection in humans and blackflies, and analysed satellite imagery to assess the loss of tree cover that can affect where blackflies live and breed. We found that the prevalence of onchocerciasis in Kakoi-Koda has declined markedly in recent years. This decline appears to be linked to the deforestation of the habitat suitable for some blackfly species, and to ivermectin distribution to treat onchocerciasis in parts of the focus. Our findings help to understand why onchocerciasis has decreased across the Kakoi-Koda focus and highlight a number of fast-flowing river segments where certain blackfly species may allow small pockets of local transmission. These results support continued, targeted monitoring to determine whether the disease is still transmitted in specific locations, where elimination interventions may still be needed.

## 1. Introduction

Onchocerciasis is a neglected tropical disease (NTD) targeted for interruption (elimination) of transmission by the World Health Organization [1]. The causative agent, the filarial nematode worm *Onchocerca volvulus*, is transmitted among humans by female *Simulium* blackfly bites [1]. More than 99% of infections occur in sub-Saharan Africa [2], where control relies mainly on community-directed treatment with ivermectin (CDTI) delivered once or twice a year to populations at risk [3]. Ivermectin is primarily microfilaricidal (kills microfilariae, the embryonic stage of the parasite) and transiently suppresses embryogenesis in adult female worms [3]. In addition, a moderate permanent sterilising effect has also been described [4]. Although macrofilaricidal activity (killing of adult worms) has been reported and quantified [5,6], adult worms can persist for many years despite periodic treatment [3,7]. Therefore, CDTI must be regularly administered for prolonged periods, accounting for both the long reproductive lifespan of adult worms [8] and the possibility of continued reinfection in areas of intense transmission. Historically, vector control (against blackflies) has also been implemented, mainly in West Africa [9,10]. The disease is characterised by a range of cutaneous and ocular clinical manifestations (including irreversible blindness), and is epidemiologically associated with neurological sequelae within the spectrum of onchocerciasis-associated-epilepsy (OAE) [11].

The Democratic Republic of the Congo contains numerous *O. volvulus* foci associated with fast-flowing rivers that harbour blackfly breeding sites [12–15]. The African Programme for Onchocerciasis Control (APOC, 1995–2015) supported the scale-up of CDTI in those areas where onchocerciasis was considered to be a public health problem (i.e., where the baseline infection level was at least mesoendemic) [16], leading to 22 CDTI projects in DRC by 2012 [17–20]. However, geographical and therapeutic coverage remained inconsistent due to insecurity, programme boundaries and reporting constraints [21].

The Kakoi-Koda onchocerciasis focus lies on the steep Ituri highland slopes above Lake Albert [18]. Rapid epidemiological mapping of onchocerciasis (REMO) surveys conducted in 2003 identified several highly endemic villages of the focus, within the Angumu, Logo and Nyarambe Health Zones (HZs) [22,23]. Routine onchocerciasis CDTI was subsequently implemented in Angumu HZ, whereas Logo and Nyarambe HZs were excluded as most of their communities were considered to have low onchocerciasis endemicity [19,20,24]. Nyarambe later received mass drug administration of ivermectin and albendazole through the lymphatic-filariasis control programme [21]. Community screening in Logo and Nyarambe HZs for clinical trials of moxidectin versus ivermectin in 2010–2011 and 2021–2023 documented a marked decline in *O. volvulus* microfilarial prevalence and infection intensity between trials [22].

The microfilarial prevalence decline observed in Logo, in the absence of CDTI, was unexpected and raised the question about how it related to the longer-term epidemiological history and wider spatial context of the Kakoi–Koda focus. In particular, the geographical extent of the decline, the location of possible residual transmission signals and their spatial correspondence with heterogeneous ivermectin delivery, entomological observations over time and landscape change remained to be elucidated. We therefore synthesised the available epidemiological, serological, programmatic, entomological and geospatial evidence in the last 25 years, including forest-cover change around streams, to describe changes in transmission indicators across the focus and to place the trial-screening findings within their broader eco-epidemiological context. While Ngave *et al.* (2026) describe the individual-level comparison of the two trial-screening periods [22], the present manuscript addresses the wider spatial and temporal evidence base. Our objectives were to identify temporal trends in infection indicators, characterise ivermectin delivery and entomological observations, and assess their descriptive spatial correspondence with tree-cover loss.

## 2. Methods

### 2.1 Ethical statement

This study was conducted under approvals from the Ethics Committee of the University of Antwerp (B300201525249; B300201733350), the Ethics Committee of Ngaliema Hospital, Kinshasa (P: Eth/436/2015) and the Ethics Committee of the University of Kinshasa School of Public Health (ESP/CE/013/2018). Permissions were also obtained from provincial and health-zone authorities. The blackfly collections reported here were conducted under a previously published protocol [18] and approved by the ethics described above. Blackfly collectors were adults (≥18 years) from local communities who provided written informed consent and were provided with ivermectin prior to participating in the collections.

### 2.2 Study setting and periods

**Fig 1:**
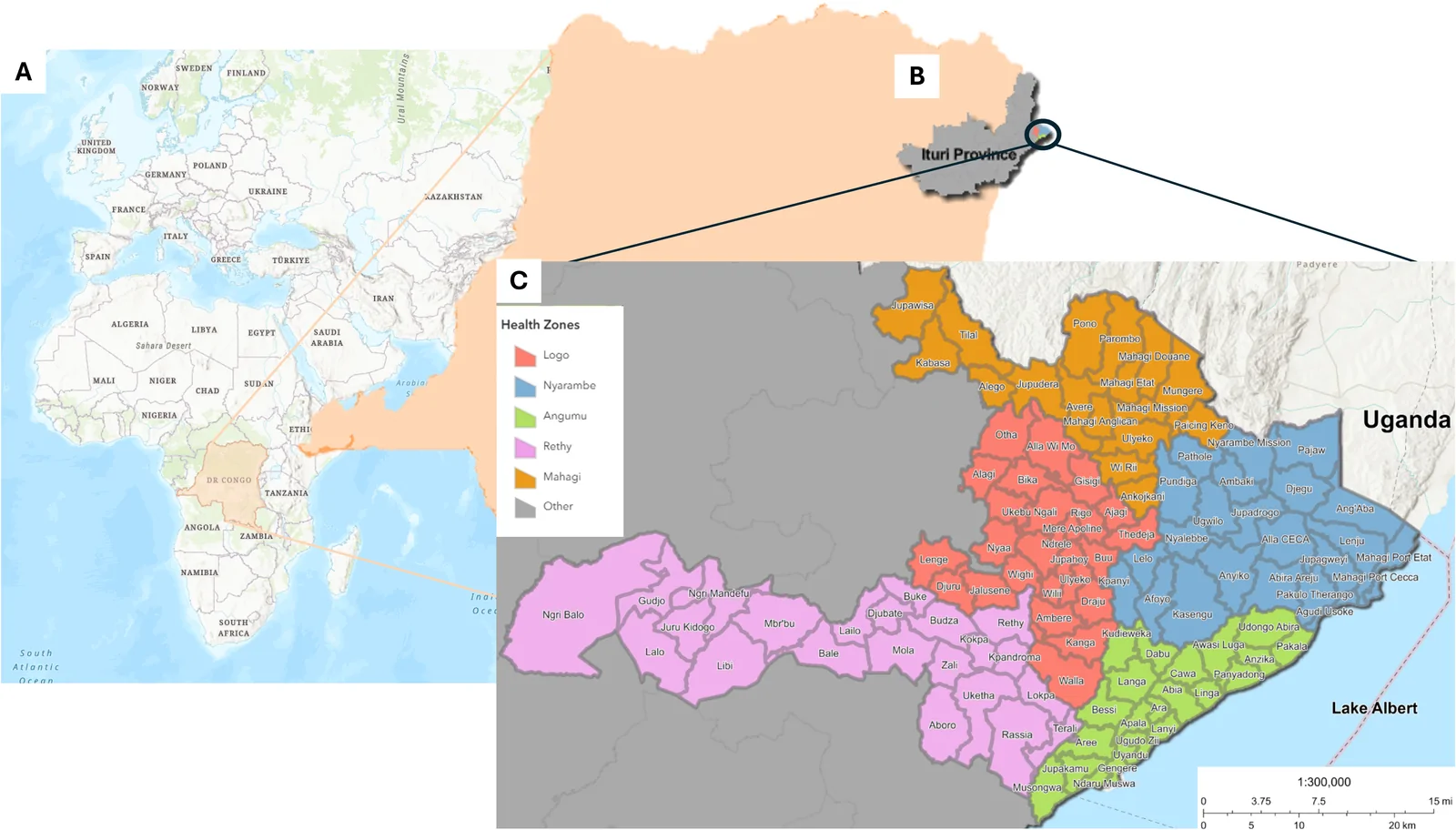
Location of the study areas in the Democratic Republic of the Congo (DRC). **(A)** Position of the DRC (orange) within Africa. **(B)** Ituri Province (dark grey) highlighted within the DRC. **(C)** Map showing the Health Zones of Angumu (green), Logo (blue), Mahagi (orange), Nyarambe (red) and Rethy (pink) in East Ituri Province, with their respective Health Areas delineated (light grey). The map was produced in ArcGIS Pro (Esri, Redlands, CA, USA) using publicly available spatial datasets (GRID3 COD – Health Areas v5.0 https://doi.org/10.7916/nnew-da26 [25], and GRID3 COD – Health Zones v5.0 https://doi.org/10.7916/d8rn-6e24 [26]).

**Fig 2:**
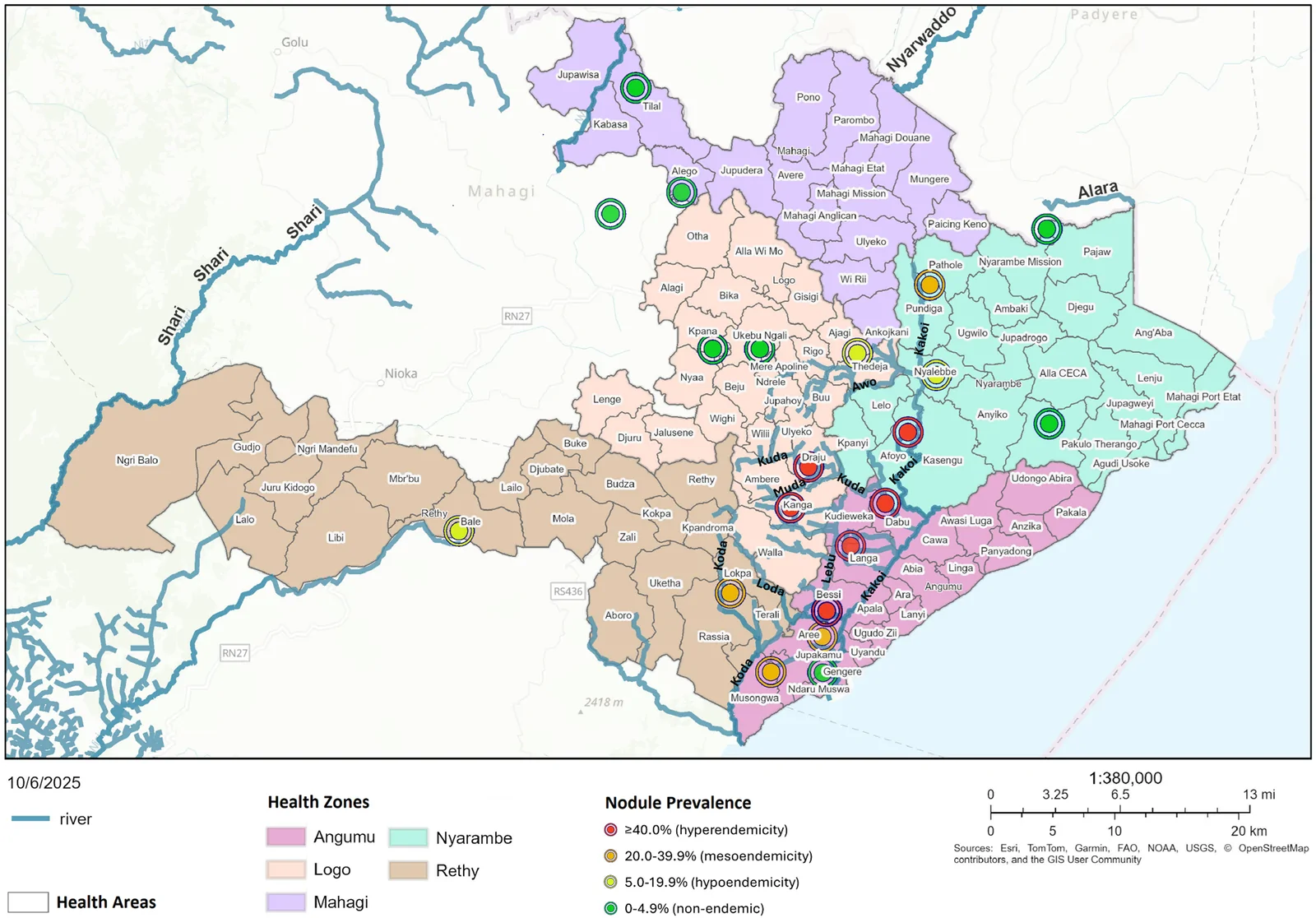
Nodule prevalence in the Kakoi–Koda onchocerciasis focus assessed in 2003 by Rapid Epidemiological Mapping of Onchocerciasis (REMO)/Rapid Epidemiological Assessment (REA) [23]. Clusters of ≥20% nodule prevalence delineate the focus in orange (mesoendemicity) and red (hyperendemicity). Areas with <20% nodule prevalence are shown as yellow (hypoendemicity) and green (non-endemic/sporadic endemicity). Only river basins pertinent to delineating the focus and major rivers are displayed (blue/teal, with names in black bold). Health zones comprising the focus are colour-shaded as follows: Angumu (pink), Logo (light pink/peach), Mahagi (purple/lilac), Nyarambe (light blue), Rethy (brown), with health areas outlined (thin black contours) with their name labels (haloed in white). The map was produced in ArcGIS Pro (Esri, Redlands, CA, USA) using publicly available spatial datasets (GRID3 COD – Health Areas v5.0 https://doi.org/10.7916/nnew-da26 [25] and GRID3 COD – Health Zones v5.0 https://doi.org/10.7916/d8rn-6e24 [26]). River and stream networks were derived from OpenStreetMap (https://www.openstreetmap.org) [43] and adjusted using ground-truth waypoints.

The demographics of the Kakoi-Koda focus are presented according to the current territorial and administrative division of health, namely territories, health zones (HZs) and health areas (HAs). The study area is located in the Ituri Province of the DRC (Mahagi and Djugu territories), in the five HZs comprising the Kakoi-Koda onchocerciasis focus, namely Angumu, Logo, Mahagi, Nyarambe and Rethy (Fig 1). The focus occupies approximately 70,000 hectares along the northeastern Ituri Highlands escarpment from the Blue Mountains near Mount Aboro down towards Lake Albert [18]. Elevation ranges from roughly 620–2,450 metres above sea level. The focus lies largely between the Kakoi and Koda rivers, with the Kuda, Lebu and Awo rivers crossing it (Fig 2) [18].

Current land cover on the highlands is predominantly savannah with fragmented primary and secondary forest blocks/patches, with forest more extensive to the west and a forest-savannah mosaic extending eastward [27]. The climate is humid tropical with weakly bimodal seasons (with some variation consisting of the main rains around July–October, main dry season November–March, minor rains in April–May and minor dry season in June) [18,24] and approximately 1,400 millimetres of mean annual precipitation [28].

### 2.3 Evidence synthesis

We conducted a scoping evidence synthesis of onchocerciasis and *Simulium* vectors in the Kakoi-Koda focus from 1980 to 31 July 2025, informed by PRISMA-ScR guidance (PRISMA extension for scoping reviews, as detailed by Tricco *et al.* 2018 [29]). We searched PubMed, Google (Scholar) and the World Health Organization’s Institutional Repository for Information Sharing (WHO IRIS) using combinations of: “onchocerciasis”, “*Simulium”*, “blackfly”, “Kakoi”, “Koda” and “Ituri” and their French equivalents (“onchocercose”, “simulie” and “mouche noire”) in titles, abstracts or mentions in the text. The use of “Ituri” as a required term ensured the retrieval of studies from both before and after the area’s official designation as Ituri Province (formerly known as Ituri District within Orientale Province). In addition to electronic searches, we reviewed: (1) programme documents from the DRC National Onchocerciasis Elimination Programme, (2) data from the Expanded Special Project for Elimination of Neglected Tropical Diseases (ESPEN) portal for DRC [21], (3) reports and protocols generated within the NSETHIO research programme [30–32], and (4) reference lists from relevant review articles for additional sources. This approach allowed us to capture both peer-reviewed publications and grey literature (reports) relevant to the context of the Kakoi-Koda focus.

The inclusion criteria included studies or reports with information on: (1) programmatic data (i.e., CDTI distribution and coverage), (2) onchocerciasis epidemiology (i.e., nodule palpation, anti-Ov16 serological and skin-snip surveys), and/or (3) entomology (i.e., human landing catches, blackfly testing for *O. volvulus* infection/infectivity, and breeding site prospections) from at least one of the five HZs of the Kakoi-Koda focus (as illustrated in Fig 1). Exclusion criteria comprised studies and documents without geographical relevance to the focus or lacking extractable outcomes. Titles, abstracts and study reports were screened, and relevant data items were extracted by LJA (location, year, population, numbers examined, diagnostic method, key epidemiological, entomological and programmatic indicators, and vector species), summarised in Results subsection 3.1, and the full extraction is provided in the (Supplementary) S1 Data. Discrepancies were resolved by discussion with the remaining co-authors.

### 2.4 Clinical trial contexts and repeated screening comparison

Four trials delivered moxidectin or ivermectin to recruited participants in the focus: the Phase III moxidectin trial 3110A1-3000 (2010–2011 [33,34]), a proof-of-concept ivermectin trial in persons with epilepsy (2017–2018 [35,36]), and the Phase IIIb moxidectin trials MDGH-MOX-3002 (2021–2023 [22,37]) and MDGH-MOX-3001 (2021–2026, ongoing, [22,38]). The three moxidectin trials recruited community residents aged ≥12 years and used four iliac-crest skin snips per person during screening [22,33,39,40]. Although the denominator for microfilarial prevalence comprised all screened residents with valid microscopy results, trial enrolment required participants to meet a specified infection threshold. The two later moxidectin trials expanded screening and relaxed enrolment criterion to positivity in at least one skin snip, because few residents met the earlier 2010–2011 criterion of ≥10 microfilariae per milligram of skin. Our repeated cross-sectional comparison is restricted to pretreatment screening outcomes, all measured using the same four-skin-snip protocol. The individual-level, village-adjusted logistic regression comparing the two screening periods is reported in the dedicated 2026 article [22]. Therefore, the present synthesis reports HA-level estimates descriptively and does not duplicate that analysis. As the ivermectin trial recruited only persons with epilepsy and used two skin snips per participant, its screening data were excluded from the repeated cross-sectional comparison.

### 2.5 Geospatial analysis of tree cover and deforestation

We quantified forest cover change using the Global Forest Change dataset by Hansen *et al.* (30-metre resolution of per cent tree cover in 2000 and annual tree loss from 2001 to 2024) [41,42]. Administrative boundaries for HZs and HAs in the focus were obtained from GRID3 COD – Health Areas v5.0 (https://doi.org/10.7916/nnew-da26) [25] and GRID3 COD – Health Zones v5.0 (https://doi.org/10.7916/d8rn-6e24) [26], respectively. Rivers and streams networks were extracted from OpenStreetMap to visualise waterways in the study area (https://www.openstreetmap.org) [43] and corrected based on ground-truth waypoints.

For the present geospatial analyses, the Hansen rasters were spatially restricted to the five HZs of the Kakoi-Koda focus and all layers were re-projected to a common coordinate system (WGS 1984 UTM Zone 36N). Forest in 2000 was defined as pixels with tree cover ≥30% (as used by Hansen *et al*. [41] and the definition followed by the DRC [44]), and forest loss as pixels that were forest in 2000 and had a non-zero loss year (2001–2024). Using zonal tabulations, we calculated for each HA the area of forest in 2000 and the area of that forest that was subsequently lost, and expressed deforestation as the percentage of 2000 forest cover lost by 2024. These HA-level estimates were then mapped, as well as summarised by HZ. We repeated this analysis with the definition of “dense forests” as pixels with tree cover ≥70% [45] to better capture closed-canopy habitats likely to be relevant for *Simulium neavei* [46]. Spatial analyses and map visualisations were performed in ArcGIS Pro (Esri, Redlands, CA, USA) and ArcGIS Online.

### 2.6 Interactive web map and data availability

To complement the static figures, we assembled an interactive web map (ArcGIS Online) that overlays epidemiological and entomological observations with geospatial context by survey year and data type. In parallel, all epidemiological, entomological, programmatic and geospatial inputs (including NSETHIO survey data not previously reported) were harmonised for the synthesis into a single master dataset accompanying this article (S1 Data). The dataset includes standardised variables, harmonised geographical identifiers, and documentation of data provenance and processing steps.

The web map includes: 1) REMO nodule surveys; 2) anti-Ov16 serological surveys (children; adults; combined); 3) skin-snip microscopy surveys (population; adolescents only; adolescents and adults); 4) freshwater-crab trapping data for *S. neavei*-associated breeding; 5) breeding-site inspections for blackfly larvae; and 6) human-biting blackfly data using human landing catch (HLC). Additionally, map layers include health-zone/health-area boundaries, rivers/streams, and annual tree-cover loss since 2000. Each layer has metadata describing source, dates, methods and any harmonisation applied. The ArcGIS interactive item is publicly accessible online (https://arcg.is/1PDyO0), and a README file is available in (Supplementary) Text A in S1 Appendix. To protect privacy, all data are aggregated at the village level and contain no personal identifiers.

### 2.7 Statistical analysis

We analysed data on programmatic treatment coverage, *O. volvulus* epidemiology (REMO nodule prevalence categories, anti-Ov16 seropositivity and skin snip (microfilarial) prevalence) and entomology (simuliid larval breeding site prospections, and HLC data). Non-normally distributed (non-Gaussian) continuous variables were summarised as medians with interquartile ranges (IQRs, 1st quartile to 3rd quartile). Categorical data are presented as counts and percentages, and, where relevant, 95% confidence intervals (95% CIs) were computed using the Wilson score method with continuity correction [47,48].

Because study designs, diagnostics, age groups and protocols varied across data sources, temporal comparisons across heterogeneous contextual sources were descriptive, and formal analyses were limited to the repeated trial-screening comparison and the within-dataset analyses described below. We harmonised metrics by data type and protocol, year data were collected, GPS coordinates (i.e., by HA and HZ for epidemiology data; river basins for entomology; village level for the interactive web map) and age strata (epidemiology: all ages ≥3 years for population prevalence data; children and adolescents as indicators of recent transmission). GPS coordinates were converted into decimal degrees and mapped in ArcGIS.

To characterise age- and sex-specific cumulative exposure to *O. volvulus*, we used data from the cross-sectional community anti-Ov16 rapid diagnostic test (RDT) survey in Logo HZ in 2016, identified in the evidence synthesis, restricted to residents aged ≥3 years with valid serology (n = 921). We focused on this community-based dataset because the remaining serology datasets were not population-representative. Ages were grouped into 3–6, 7–10, 11–20, 21–30, 31–40, 41–50 and ≥51 years to ensure sufficient sample sizes per band. For each age group and sex, we estimated anti-Ov16 seroprevalence with 95% CIs using the Wilson score method with continuity correction and displayed these estimates in an age- and sex-specific seroprevalence plot. We fitted a logistic regression model with age, sex and their interaction, adjusting for health area, to assess age and sex differences in cumulative exposure.

For person-level paired comparisons of skin-snip microscopy and field anti-Ov16 RDT among children aged 3–10 years (indicative of ongoing transmission), we reported per cent agreement and Cohen’s kappa (κ) with asymptotic 95% CIs. Marginal homogeneity was assessed using McNemar’s test with mid-p two-sided p-values due to the small number of discordant pairs [49]. Skin-snip results were taken as a reference to estimate the sensitivity and specificity of anti-Ov16 RDT in the field, with exact (Clopper-Pearson) 95% CIs given the small sample size and sparse discordant data. Analyses were performed in R (version 4.4.1) using base functions and the packages DescTools (κ, CIs) [50] and epiR (diagnostic accuracy) [51].

## 3. Results

### 3.1 Evidence base and data coverage

We assembled epidemiological, entomological and control programme data for the Kakoi-Koda focus from 1980 to 2025. Table 1 summarises designs, populations, procedures, metrics, years, areas and key limitations for each data stream. Epidemiological sources included REMO nodule palpation, anti-Ov16 serology from community, case-control and cohort surveys investigating OAE, and community and study-based skin-snip surveys (including screening datasets from the 2010–2011 and 2021–2023 moxidectin clinical trials). Entomology encompassed targeted inspections of breeding sites and freshwater crabs, (informal) “spot checks” of human-biting blackfly species by HLC and use of genus-specific mitochondrial 16S rDNA gene and species-specific NADH dehydrogenase subunit 5 (ND5) gene polymerase chain reaction (PCR) for the detection of *O. volvulus* DNA in flies’ heads (infectivity prevalence) or heads and bodies (infection prevalence) in blackfly samples. Programme documents provided contextual information on ivermectin distribution history (i.e., geographical and therapeutic coverage) by HZ and calendar year. The next subsections detail (3.1.1) CDTI delivery histories by HZ and HA, (3.1.2) temporal patterns in infection indicators, and (3.1.3) vector species and transmission ecology. HA-level estimates with denominators and 95% confidence intervals for every data stream are tabulated in Table A–E in S1 Appendix, and the individual-level records underlying them are provided in S1 Data.

**Table 1:** Epidemiological and entomological data sources and methods in the Kakoi–Koda onchocerciasis focus, Ituri Province, DRC.

Data design	Purpose / Indicator	Target population / Sampling	Methods	Metrics reported	Coverage sources (years: area [source of data])	Key limitations / Sources of bias
**CDTI programme coverage**	Geographical and therapeutic coverage of ivermectin distribution; rounds per year	Coverage of the total population Data aggregated by community, HA or HZ	Community-directed treatment registers were collated at the HZ level Community-based and case-control surveys were used as supplementary checks where available	Geographical coverage: proportion of targeted communities treated by HA or HZ Therapeutic coverage: number treated in the total population (all ages) of targeted area Delivery pattern: number of ivermectin rounds per year; programme platform	1) 2014–2023 ESPEN HZ-level coverage (ivermectin for onchocerciasis and/or lymphatic filariasis) [21]; 2) & 3) August and October 2015 epilepsy case-control (persons with epilepsy and community controls) coverages: Logo HZ (Draju HA) and Rethy HZ (Lokpa HA) reported ivermectin intake used to confirm occurrence/level of ivermectin delivery in 2014 [52–54]; 4) 2021 children (aged 5–9 years) community survey: Nyarambe HZ (Kpanyi HA) 2020 coverage check (published with this manuscript^a^); 5) 2021–2023 moxidectin trial: Logo HZ (Draju, Kanga, Thedeja HAs) and Nyarambe HZ (Kpanyi HA)	Administrative denominators uncertain (migration, insecurity, underestimated census); incomplete or delayed reporting Short distribution windows; limited independent verification (lack of coverage survey data) Interruptions to treatment due to insecurity and the COVID-19 pandemic
**Nodule palpation surveys**	REMO/REA: rapid endemicity classification for programme control decisions Epilepsy cohort nodule palpation: presence of palpable nodules within a defined epilepsy cohort; contextualises onchocerciasis exposure in persons with epilepsy	REMO/REA: 30–50 adults, typically men aged ≥20 years with ≥10 years residence per village or sentinel site [20] Epilepsy cohort: all consenting persons with epilepsy (both sexes, all ages) enrolled in the cohort [55]	Standardised palpation of common body sites (e.g., bony prominences) for onchocercal subcutaneous nodules by trained examiners [20]	REMO/REA: nodules per person and prevalence (%) in adults; used to classify endemicity (e.g., < 20% hypoendemic, 20–39.9% mesoendemic, ≥ 40% hyperendemic) [17] Epilepsy cohort: nodule prevalence (%) within the cohort [55]	REMO/REA: 1) 2003: Angumu HZ (Aree, Bessi, Dabu, Langa, Musongwa HAs), Logo HZ (Draju, Kanga, Thedeja HAs), Mahagi HZ, Nyarambe HZ (Afoyo, Nyalebbe, Pundiga HAs), and Rethy HZ (Rassia HA) [23]; 2009: Logo HZ (Otha HA) [56]; 3) September 2015: Logo HZ (Draju HA) and Rethy HZ (Lokpa, Rassia HAs) [52]; 4) November 2017: Logo HZ (Draju, Kanga, Walla HAs) (nodule findings published with this manuscript, collected during study [57]) Epilepsy cohort: 5) October 2017: Logo HZ (Draju HA) [55]	Palpable nodules are used to indicate the presence of adult worms; however, their diagnostic value is poorer in low transmission (hypoendemic) areas [19] Focal transmission may be missed; limited sensitivity under changes in transmission conditions [58] REMO/REA: standardised adult-only, male-predominant samples improve comparability with (and conversion into) microfilarial prevalence but may not be population-representative Epilepsy cohort: findings apply to persons with epilepsy only
**Anti-Ov16 serology**	All-age surveys: exposure to *O. volvulus* infection (cannot distinguish between past and current) Child surveys (3–10 years): Indicator of ongoing transmission	All-age surveys: Resident population aged ≥3 years (community or study-specific samples) Child surveys: community samples of children aged 3–10 years	SD Bioline Onchocerciasis Ov16 IgG4 rapid diagnostic test (RDT) conducted in the field on capillary blood (finger-prick) samples [54]; results read after 20 minutes [59] Anti-Ov16 IgG4 ELISA in the laboratory [22]	Anti-Ov16 seroprevalence (%) in the community (all-age) or among children aged 3–10 years	All-age surveys: 1) 2015 community survey (ESPEN): Angumu, Mahagi, Nyarambe and Rethy HZs [22]; 2) October 2015 epilepsy case-control study: Logo HZ (Draju HA) and Rethy (Lokpa, Kpandroma, Rassia HAs) [52,53]; 3) August 2016 community survey: Logo HZ (Draju, Kanga, Ulyeko HAs) [54]; 4) October 2017 epilepsy cohort study: Logo HZ (Draju HA) [55] Child surveys: 5) 2018 community survey: Logo HZ (Kanga, Walla HAs) (published with this manuscript^a^); 6) February 2021 community survey: Nyarambe HZ (Kpanyi HA) (published with this manuscript^a^)	RDT and ELISA have limited accuracy (sensitivity/specificity); cannot time infection or separate past from current [58]; potential reader variability (e.g., faint bands) in field RDTs [60] Some sampling frames are not fully representative of the population (e.g., epilepsy case-control/cohort design), but they still inform on onchocerciasis exposure and recent transmission, especially in young children
**Skin-snip microscopy surveys**	Current *O. volvulus* infection (prevalence and intensity)	Moxidectin trials: residents aged ≥12 years; 4 skin snips per person [22] Other surveys: residents aged ≥5 years; 2 skin snips per person (community-based and epilepsy studies) [55]	Iliac-crest snips with a 2-millimetre Holth punch; 8–24 hours incubation in isotonic saline solution in flat-bottom microtitre plates; emerged microfilariae counted by inverted microscopy [22,55]; results expressed as microfilariae presence and number per skin snip	Microfilaria-positive prevalence (%) and average microfilariae counts per person to calculate infection intensity in the population (moxidectin trials: geometric mean number of microfilariae per milligram of skin; other surveys: geometric mean number of microfilariae per skin snip)	Moxidectin trials: 1) 2010–11: Logo HZ (Ambere, Buu, Draju, Jupahoy, Kanga, Ndrele, Ulyeko, Walla HAs) and Nyarambe HZ (Kpanyi HA) [39]; 2) 2021–2023: Logo HZ (Draju, Kanga, Thedeja HAs) and Nyarambe HZ (Kpanyi HA) [22] Other surveys: 3) 2015 community survey (ESPEN): Angumu, Mahagi, Nyarambe and Rethy HZs [22]; 4) August 2015 epilepsy case-control study: Logo HZ (Draju HA) and Rethy HZ (Lokpa HA) [54]; 5) October 2015 epilepsy case-control study: Logo HZ (Draju HA) and Rethy HZ (Lokpa, Rassia HAs) [52,53]; 6) October 2017 epilepsy cohort study: Logo HZ (Draju HA) [55]	Slightly invasive and acceptance bias; limited infection detection after CDTI delivery and in low-transmission areas [58] 2 versus 4 snips impacts sensitivity [61] Selection criteria in trials (preference for higher microfilarial loads) and in epilepsy studies (onchocerciasis exposure associated with epilepsy) limit population representativeness [22,39,55]
**Blackfly breeding site surveys**	Vector breeding/presence prospection and pre-imaginal (larval and pupal) habitats	Primarily river sites resembling *S. damnosum* s.l. habitats Freshwater crab habitats for *S. neavei*	Hand collection of *Simulium* spp. larvae and pupae from rocks/vegetation Freshwater crabs captured by hand or baited basket traps; blackfly preservation in 70% ethanol	Presence/absence of simuliid immature stages; *Simulium* species identification, including non-anthropophagic species Presence/absence of crabs infested *with S. neavei* immature stages.	*Simulium* breeding sites: 1) August-October 2015, August 2016, April and August 2017: Awo River, Kakoi River basin (Logo HZ: Thedeja HA; Nyarambe HZ: Nyalebbe HA), downstream Kakoi River basin (Nyarambe HZ: Afoyo HA), Kuda River basin (Angumu HZ: Kudieweka HA; Logo HZ: Draju HA; Nyarambe HZ: Kpanyi HA) and Koda River basin (Rethy HZ: Lokpa, Rassia HAs) [18] Crab prospections (for *S. neavei*): 2) October 2009: Awo River, Kakoi River basin (Logo HZ: Alla Wi Mo HA; Nyarambe HZ: Nyalebbe HA) [56]; 3) September 2015, June and August 2016: Awo River, Kakoi River basin (Nyarambe HZ: Lelo, Nyalebbe HAs), Kakoi River basin (Logo HZ: Draju HA) and Koda River basin (Rethy HZ: Lokpa, Rassia HAs) [18]	Targeted prospection (no prior breeding-site mapping); incomplete spatial and seasonal coverage [18]
**Adult blackfly collections**	Identify anthropophilic (human-biting) *Simulium* species present in the area; when tested, detect ongoing transmission via *O. volvulus*-positive flies	Informal human landing catches (HLC) for adult (imaginal) blackfly collection at riverbank prospection sites	HLC “spot checks” (for 30–60 minutes) per site; captured flies preserved in 70% ethanol; blackfly morphological identification to species; heads and bodies separated; qualitative real-time triplex PCR (16S, ND5) for *O. volvulus* positivity per fly.	Presence and species identification of anthropophagic blackflies *O. volvulus* infection rate (bodies) and infective rate (heads)	Informal HLCs Prospections: August, October and November 2017, February–April 2018: Kuda River basin (Logo HZ: Draju HA; Nyarambe HZ: Afoyo, Kpanyi HAs; Rethy HZ: Rassia HA) [18]	No parity dissections; no formal collector rotation or fixed timetable to allow estimation of biting rates; seasonal and diurnal gaps; ethical concerns inherent to HLCs; incomplete spatial coverage of the focus [18]

**Abbreviations**: CDTI = community-directed treatment with ivermectin; ESPEN = Expanded Special Project for Elimination of Neglected Tropical Diseases; HA = health area; HZ = health zone; REMO/REA = Rapid epidemiological mapping of onchocerciasis/Rapid Epidemiological Assessment.

^a^Community survey data generated within the NSETHIO project [30] (see Methods [31]). Individual-level records are provided in S1 Data and summarised in Table C in S1 Appendix.

#### 3.1.1 CDTI delivery.

Ivermectin delivery in the Kakoi-Koda focus differed between HZs and by target disease. For onchocerciasis control, CDTI began in Angumu HZ in 2009 (ESPEN repository-derived therapeutic coverages of 80–82% of the total population) and in Rethy HZ in 2012 (68–81% coverage). In contrast, in Nyarambe HZ, mass drug administration of ivermectin and albendazole for lymphatic filariasis commenced in 2016 (80–84% coverage) [21]. Logo and Mahagi HZs had not implemented CDTI as of 2025 [21]. Ivermectin delivery was temporarily interrupted in Ituri in 2020 due to the COVID-19 pandemic [21].

Beyond routine CDTI, the trials described in subsection 2.4 treated only their recruited participants. The 2010–2011 moxidectin trial administered single doses to 315 (8 milligrams of moxidectin) and 157 (150 micrograms of ivermectin/kilogram of body weight) participants in Draju and Kanga HAs (Logo HZ) [33,34]. Secondly, the 2017–2018 ivermectin proof-of-concept study gave one to three ivermectin doses to 197 persons living with epilepsy in Draju, Kanga, Walla, Ulyeko, Thedeja HAs (Logo HZ) [35,36]. Only 1.5% (3/197) of these participants self-reported prior ivermectin use [35]. Thirdly, the 2021–2023 moxidectin trial enrolled additional participants on moxidectin or ivermectin (trial results pending) [22,37], and fourthly, the ongoing 2021–2026 trial delivers one to three annual or five biannual doses (results are pending for both trials) [22,38]. At the 2021–2023 screening, self-reported ivermectin use in the preceding five years was 0.4% (29/7,547) in Draju, Kanga and Thedeja HAs (Logo HZ) and 0.9% (9/1,047) in Kpanyi HA (Nyarambe HZ) [40].

In addition to the clinical trials, two epilepsy case-control surveys provided local verification of ivermectin intake. The first survey was conducted in August 2015, with a population sampled aged 3–75 years (median: 22 years, IQR: 14–30 years) and 52.4% females. The second survey was conducted in October 2015, with a population sampled aged 4–35 years (median: 17 years, IQR: 12–24 years) and 53.6% females. The former survey (August) found a self-reported ivermectin intake in 2014 of 1.5% (1/69) in Logo HZ (Draju HA) and of 75.4% (43/58) in Rethy HZ (Kpandroma, Lokpa HAs) [52,53]. The latter survey (October) found a self-reported ivermectin intake in 2014 of 9.1% (4/45) in Logo HZ (Draju HA) and of 73.3% (11/15) in Rethy HZ (Lokpa HA) [52,53]. Among respondents who reported taking ivermectin in the latter survey, the median cumulative number of ivermectin rounds taken was 3 (IQR: 3–3; range: 0–3 rounds).

A self-reported community survey conducted in 2021 in Nyarambe HZ (Kpanyi HA) among ivermectin-eligible children aged 5–9 years, reported here for the first time, recorded 2020 ivermectin coverage of 91.6% (87/95) (S1 Data). Among treated children, the median cumulative number of ivermectin rounds received by 2020 was 2 (IQR: 2–2; range: 1–3 rounds). Participants had a median age of 7 years (IQR: 7–9 years), and 56.4% were female.

#### 3.1.2 Longitudinal trends in *O. volvulus* infection indicators.

Across data sources, indicators of *O. volvulus* infection declined between early observations (REMO 2003; skin‐snip screening data in 2010–2011) and more recent surveys (in 2015–2023). To document this pattern, we first summarise baseline endemicity from nodule palpation surveys, then present serological evidence (all-age exposure and child anti-Ov16 as an indicator of recent transmission), and finally describe skin-snip prevalence and intensity between the 2010–2011 and 2021–2023 moxidectin trial and OAE studies (2015–2017).

**Nodule palpation prevalence to establish baseline endemicity**

A proportion of adult *O. volvulus* worms reside in subcutaneous nodules or onchocercomata [62], making nodule palpation a useful REA method for the evaluation of prevalence in areas of moderate to high endemicity. In 2003, REMO/REA nodule palpation identified several areas as meso- or hyperendemic (≥20% nodule prevalence) for onchocerciasis, permitting an initial delineation of the Kakoi-Koda focus (Fig 2) [23]. A median of 30 individuals (IQR: 30–40) aged ≥20 years per HA (one village) were examined, of which 82.7% (617/746) were male.

Hyperendemicity (nodule prevalence ≥40%) was concentrated between the Awo River (northeast Kuda branch) and the Kakoi, Kuda and Lebu River basins, in Bessi, Dabu and Langa HAs (Angumu HZ; 70–90%), Draju and Kanga HAs (Logo HZ; 98% and 70%) and Afoyo HA (Nyarambe HZ; 52%). Mesoendemicity (nodule prevalence ≥20% but <40%) extended upstream in the Kakoi and the Koda and Loda River basins, in Aree and Musongwa HAs (Angumu HZ; 36% and 35%), Pundiga HA (Nyarambe HZ; 30%) and Rassia HA (Rethy HZ; 37%). Hypoendemic areas (nodule prevalence ≥5% but <20%) were recorded around the Awo River in Thedeja HA (Logo HZ; 7%) and Nyalebbe HA (Nyarambe HZ; 16%). An additional survey in 2009 in Otha HA classified it as non-endemic (Logo HZ; 4%) [56]. (Areas with <10% microfilarial prevalence are considered non-endemic/sporadic endemicity, corresponding to a nodule prevalence <5% [16,63,64].)

Using similar methods, a 2015 REA study was conducted in several villages within three HAs that reported high OAE prevalence [52]. Nodule prevalence was 34.6% in Draju (Logo HZ), and 29.6% in Rassia and 17.2% in Lokpa (Rethy HZ). Among those with nodules, the median number was one (IQR: 1–1; range: 1–6). In November 2017, a further REA conducted alongside a study of urinary N-acetyltyramine-O,β-glucuronide [57], and whose nodule findings are reported here for the first time, palpated 94 men aged ≥20 years in villages of Logo HZ reporting a high OAE prevalence. Nodule prevalence was 44.4% in Draju, 56.8% in Kanga and 20.0% in Walla. Among those with nodules, the median number was one (IQR: 1–1; range: 1–4).

A last nodule palpation survey in October 2017 examined 38 adults aged ≥20 years living with epilepsy in Draju HA (Logo HZ), including the same villages as in the 2003 and 2015 surveys, and recorded a nodule prevalence of 21.1% (95% CI: 10.1–37.8) [55]. Denominators, villages sampled, endemicity classifications and 95% confidence intervals for all nodule surveys are given in Table A in S1 Appendix.

**Anti-Ov16 serological surveys to assess *O. volvulus* cumulative exposure**

Anti-Ov16 serology was measured (with whole blood) using the SD Bioline anti-Ov16 RDT (Onchocerciasis IgG4 rapid test, Abbott Standard Diagnostics, Inc., Yongin, Korea; method detailed in Table 1). Anti-Ov16 serosurveys conducted during 2015–2017, among residents aged ≥3 years and, in one survey, among men aged ≥20 years, indicate high cumulative exposure in both Logo and Rethy HZs (Table B in S1 Appendix), in agreement with 2003 REMO meso- to hyperendemic classifications in Fig 2. Seropositivity was generally higher in Draju HA (Logo HZ).

Community sampling (among 921 residents aged ≥3 years) in Logo in 2016 found that Draju seroprevalence was elevated at 36.1% (95% CI: 31.6–40.8) compared with 25.8% (95% CI: 22.0–30.1) in Kanga HA (Table B in S1 Appendix) [54]. The estimate for Ulyeko HA is based on a very small sample (2/7 seropositive), providing limited information beyond indicating some *O. volvulus* exposure in that HA. In 2017, the same 94 men aged ≥20 years palpated for nodules in Draju, Kanga and Walla HAs were also tested by anti-Ov16 RDT, and cumulative lifetime exposure was likewise high, at 40.7% (95% CI: 23.0–61.0), 64.9% (95% CI: 47.4–79.3) and 26.7% (95% CI: 13.0–46.2) respectively [57].

Epilepsy case-control and cohort samples from 2015–2017 showed substantial cumulative exposure in parts of Logo and Rethy but were not population-representative (Table B in S1 Appendix). In the 2015 epilepsy case-control, seroprevalence in Draju was 41.2% (95% CI: 32.2–50.8), closely followed by Rassia HA (Rethy HZ) at 37.8% (95% CI: 22.9–55.2), and lower in Lokpa HA (Rethy HZ) at 29.4% (95% CI: 17.9–44.0) [52,53]. These patterns are consistent with the 2015 REA described above, with moderate nodule prevalence in Draju and Rassia (30–35%) and lower prevalence in Lokpa (17%). In the 2017 epilepsy cohort, a seroprevalence of 64.4% (95% CI: 52.2–75.0) was documented in Draju, statistically significantly higher than the 28.0% (95% CI: 22.9–33.6) seroprevalence from the pooled Kanga/Ulyeko/Thedeja/Walla HAs (Logo HZ) cohort [55].

**Fig 3:**
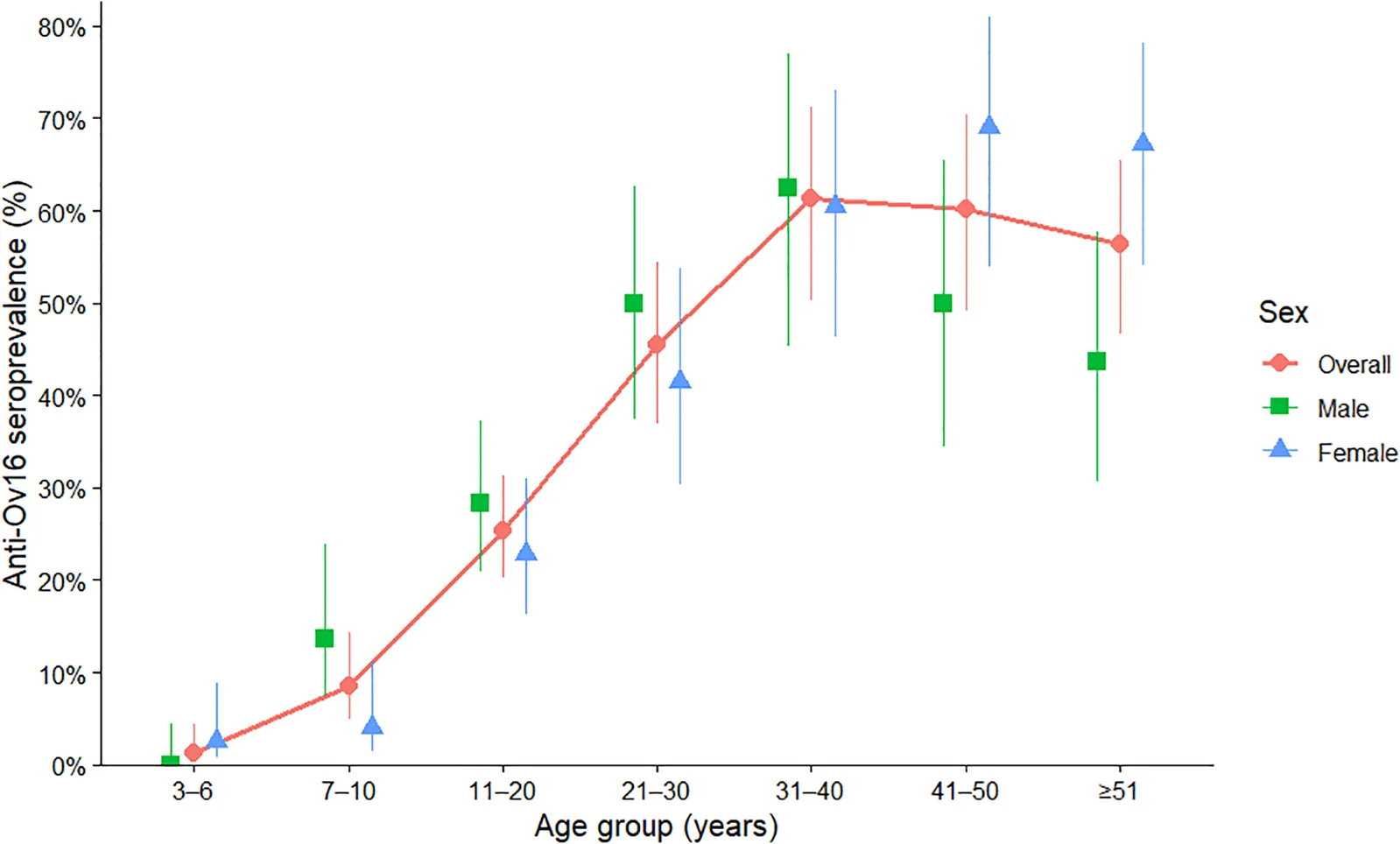
Age-specific anti-Ov16 seroprevalence by sex in the 2016 community survey in Logo Health Zone (Draju, Kanga and Ulyeko Health Areas) [54]. Red circles (joined by red solid line) show seroprevalence estimates with 95% Wilson confidence intervals (error bars) by age group overall, with green squares indicating males and blue triangles indicating females (male and female estimates are slightly offset horizontally for better visualisation).

Age- and sex-specific anti-Ov16 seroprevalence from the 2016 community survey in Logo HZ (Table B in S1 Appendix) is shown in Fig 3. Seroprevalence rose steeply from childhood to early adulthood and then plateaued around 60–70% in older adults, consistent with the hyperendemic status indicated by the 2003 REMO survey. Seroprevalence tended to be higher in males in the younger age groups, but higher in females in the older age groups. In a logistic regression including a sex-by-age-group interaction and adjusting for HA, there was no strong evidence of sex differences up to 40 years of age (odds ratio female versus male (OR_f/m_): 0.77, p = 0.19 for ages 3–30 years; OR_f/m_: 0.92, p = 0.85 for 31–40 years), although point estimates suggested slightly higher exposure in men. In contrast, among adults aged >40 years, women had markedly higher seroprevalence than men (OR_f/m_: 2.46, p = 0.004).

An ESPEN repository-derived anti-Ov16 ELISA dataset from 2015 targeted non-endemic villages surrounding the Kakoi-Koda focus, and therefore is presented Text B in S1 Appendix [22].

**Child anti-Ov16 RDT serological surveys to indicate *O. volvulus* ongoing transmission**

In Logo HZ (Draju HA), the 2015 epilepsy case-control survey identified anti-Ov16 RDT seropositivity in children aged 3–6 (1/7) and 7–10 (2/19) years (Table C in S1 Appendix) [52,53]. In the subsequent 2016 community survey conducted in the same locality, no child aged 3–6-years was seropositive (0/70), whereas 10.3% of 7–10-year-olds were (7/68) [54]. A similar pattern was observed in Kanga HA (Logo HZ) in 2016, with seroprevalence of 2.2% among 3–6-year-olds (2/90) and 9.6% among 7–10-year-olds (7/73). No seropositive children were identified in the 2017 epilepsy cohort across Draju, Kanga, Ulyeko, Thedeja, and Walla HAs (Logo HZ; 0/56) [55]. In 2018, a smaller community survey in Kanga and Walla, reported here for the first time, detected no seropositive 3–6-year-olds (0/4 in Kanga, 0/14 in Walla) and one seropositive 7–10-year-old (1/21 in Kanga, 0/21 in Walla; Table C in S1 Appendix).

In Rethy HZ, one of 17 children tested in Lokpa and Rassia HAs was seropositive in the 2015 case-control (Table C in S1 Appendix) [52,53]. In Nyarambe HZ (Kpanyi HA), none of the 95 children aged 7–9 years tested in 2021 was seropositive in a community survey reported here for the first time (Table C in S1 Appendix).

**Skin-snip microscopy surveys for moxidectin trials**

Skin-snip microscopy data were assembled from sources using two different protocols: 1) moxidectin trial screenings (2010–2011 and 2021–2023), which obtained four snips per participant from community-recruited residents aged ≥12 years; and 2) other surveys (2015–2017), which obtained two snips per participant in OAE case-control and cohort designs (methods detailed in Table 1). Because snip number and study design differ, prevalence and intensity are only compared within (but not between) these two groups.

In the 2010–2011 moxidectin trial screening, large numbers of residents were screened in Logo HZ, mostly in Draju (number = 1,149) and Kanga (n = 161) HAs (Table 2) [22]. Additionally, a smaller number of residents were screened in Nyarambe HZ (Kpanyi HA; n = 36). Village-level sample sizes were small and heterogeneous (median: 7, IQR: 3–25, range: 1–324 residents per village). *O. volvulus* microfilarial prevalence reached 79% in Draju (908/1,149) and 72% in Nyarambe, consistent with the abovementioned REMO (2003) hyperendemic classifications and all-age anti-Ov16 findings (2015–2017; Table B in S1 Appendix).

**Table 2:** Skin‐snip results from cross-sectional moxidectin trial screenings in 2010–2011 and 2021–2023, by age group, health zone and health area [22]. Community-recruited participants aged ≥12 years provided four skin snips each. *Onchocerca volvulus* infection prevalence is shown with Wilson score 95% confidence intervals, and infection intensity (microfilariae per milligram of skin). The proportion of females and males surveyed is not available.

Health Area	Study number^c^	Years	Number examined aged ≥18; 12–17 years	Skin snip microfilaria-positive/Total examined (prevalence %, 95% CI)		Mean infection intensity overall (mean mf+ intensity; range) mf/mg skin
**All ages,** aged 12–93 years	**Aged 12–17 years**
**Logo Health Zone**						
Draju	1	2010-11	1,057; 92	908/1,149 (79.0, 76.5–81.3)	70/92 (76.1, 65.9–84.1)	26.2 (33.1; 0.1–299.4)
	2	2021-23	2,803; 1,212	363/4,015 (9.0, 8.2–10.0)	38/1,212 (3.1, 2.3–4.3)	1.2 (13.0; 0.1–177.8)
Kanga	1	2010-11	151; 10	111/161 (68.9, 61.1–75.9)	4/10 (40.0, 13.7–72.6)^d^	17.1; (24.8; 0.1–200.8)
	2	2021-23	1,967; 706	231/2,673 (8.6, 7.6–9.8)	26/706 (3.7, 2.5–5.4)	1.1; (13.2; 0.1–99.2)
Thedeja	2	2021-23	747; 112	32/859 (3.7, 2.6–5.3)	0/112 (0.0, 0.0–4.1)	NA; (NA; 0.3–154.8)
Ambere/Buu/Jupahoy/Ndrele/Ulyeko/Walla^a^	1	2010-11	57; 6	30/63 (47.6, 35.1–60.5)	5/6 (–^e^)	NA; (NA; 0.2–91.1)
**Nyarambe Health Zone**						
Kpanyi	1^b^	2010-11	36; 0	26/36 (72.2, 54.6–85.2)	0	10.6; (14.6; 0.1–135.6)
	2	2021-23	690; 357	30/1,047 (2.9, 2.0–4.1)	0/357 (0.0, 0.0–1.3)	0.4; (13.2; 0.2–53.8)

**Abbreviations**: CI = confidence interval; IQR = interquartile range (1^st^ quartile to 3^rd^ quartile); mf = microfilariae; mf + intensity = microfilarial load among those with microfilaria-positive skin snips; mg = milligram; NA = not available, as the data were only available aggregated.

a Contiguous health areas in Logo Health Zone: Ambere (12/14 positive), Buu (3/4), Jupahoy (4/8), Ndrele (0/10), Ulyeko (4/17), Walla (7/10).

b Includes five adults who were microfilaria-positive by skin-snip microscopy from the Lelo Health Area in Nyarambe Health Zone, bordering Kpanyi.

^c^Study number is the numerical label (1–2) grouping rows belonging to the same data collection round; identical study numbers indicate the same study conducted in multiple health areas.

^d^Small sample size (number = 10–30).

^e^Prevalence and 95% confidence interval are not reported for very small samples (number < 10).

Age-stratified skin snip results in Logo in 2010–2011 showed no clear signs of reduced infection in adolescents (Table 2). In particular, in Draju, overall prevalence was 79.0% (95% CI: 76.5–81.3), similar to the prevalence in 12–17-year-olds of 76.1% (95% CI: 65.9–84.1); in Kanga, overall prevalence was 68.9% (95% CI: 61.1–75.9) and prevalence among 12–17-year-olds was 40.0% (95% CI: 13.7–72.6), with the wide adolescent CI overlapping the adult estimate.

In the 2021–2023 moxidectin trial screening, sampling effort increased across the same HAs of Draju (n = 4,015), Kanga (n = 2,673) and Kpanyi (n = 1,047; Table 2), with larger village-level samples (median: 50, IQR: 21–218, range: 3–950 residents per village). Prevalence was markedly reduced in all three HAs (approximately 3–9% overall prevalence). Declines were most pronounced in younger age groups. For example, in Draju, the prevalence among 12–17-year-olds fell from 76.1% (95% CI: 65.9–84.1) in 2010–2011 to 3.1% (95% CI: 2.3–4.3) in 2021–2023. In Kpanyi, although the 2010–2011 sample was limited, repeat adult sampling across the same villages showed a reduction from 72.7% (16/22) to 2.0% (1/49) (Fisher’s exact p < 0.001). Thedeja HA (in Logo HZ) was also screened in 2021–2023 (but not in 2010–2011) as the trial expanded sampling to identify additional *O. volvulus*-infected residents for enrolment, and it showed the lowest microfilarial prevalence among the four HAs (3.7%, 95% CI: 2.6–5.3), consistent with its hypoendemic status according to the 2003 REMO surveys.

Infection intensity mirrored these prevalence declines (Table 2). Community infection intensity (i.e., microfilariae per milligram of skin) was substantially lower in 2021–2023 than in 2010–2011. Illustratively, Draju decreased from 26.2 (range: 0.1–299.4) to 1.2 (range: 0.1–177.8), Kanga from 17.1 (range: 0.1–200.8) to 1.1 (range: 0.1–99.2) and Kpanyi from 10.6 (range: 0.1–135.6) to 0.4 (range: 0.2–53.8). This decrease in microfilarial load was also observed when only considering those with microfilaria-positive skin snips (i.e., mf+ intensity in Table 2).

In the smaller 2010–2011 screening sites, microfilaria-positive skin snips were frequent in Ambere (12/14) and Walla (7/10) HAs, which border the Draju and Kanga HAs classified as hyperendemic by REMO in 2003, but absent in the northernmost HA tested (Ndrele, 0/10), which borders the non-endemic Ukebu Ngali HA (Table 2).

**Skin-snip microscopy surveys from other studies**

Across the 2015–2017 epilepsy case-control and cohort surveys in Logo (e.g., Draju HA) and Rethy (e.g., Lokpa and Rassia HA) HZ, the highest all-age skin-snip prevalence values were consistently observed in Draju (range: 46–63%) compared to more moderate microfilaria-positive skin-snip prevalence values in other HAs (range: 20–47%; Table D in S1 Appendix), concordant with the all-age anti-Ov16 seroprevalence for these areas (Table B in S1 Appendix) [52–55]. Infection intensity was relatively high in most surveyed HAs (range: 13–93 microfilariae per skin snip), excluding Lokpa (range: 2–8 microfilariae per skin snip; Table D in S1 Appendix), consistent with Lokpa’s hypo- to mesoendemicity status by nodule prevalence (2003–2015) compared to meso- to hyperendemicity in Rassia and Draju.

Lower proportions of microfilaria-positive skin snips were detected among children aged 3–11 years in Logo HZ (14–33% in Draju HA; Table D in S1 Appendix) compared with adolescents (48–60% in Draju HA among 12–17-year-olds), in agreement with the low anti-Ov16 seroprevalence (by RDT) in Logo among 3–10-year-olds (0–19% in 2015–2018; Table C in S1 Appendix). In contrast, child infection patterns in Rethy HZ did not show a similarly marked decline (33% in Rassia HA; Table D in S1 Appendix) relative to adolescents (30% in Rassia among 12–17-year-olds).

An ESPEN repository-derived skin-snip dataset from 2015 sampled non-endemic villages surrounding the Kakoi-Koda focus, and therefore is presented Text B in S1 Appendix [22].

#### 3.1.3 Vector species and transmission ecology.

To characterise potential vectors and where transmission may persist, we combined information from targeted larval/pupae breeding site prospections in both crab-associated and non-crab river habitats, together with informal “spot-check” HLCs to identify anthropophagic *Simulium* species and individual PCR testing of a subset for *O. volvulus* 16S and ND5 genes across the main basins of the focus (methods detailed in Table 1).

**Breeding sites prospection**

Crab-associated prospections for the *S. neavei* group conducted in October 2009 detected *S. neavei* larvae at one site within the focus, specifically at the Kakoi River basin (larvae in 11/12 crabs in Lelo HA, Nyarambe HZ) [56]. Two crabs from the south-eastern Awo River (Nyalebbe HA of Nyarambe HZ) were not infested with larvae, and no crabs were found further north-west at the limit of the focus (Alla Wi Mo HA, Logo HZ). In 2015–2016, no infested crabs were detected despite targeted sampling along the Kuda River basin (Logo HZ, Draju HA: 0/35 crabs from nine sites), the Koda River basin (Rethy HZ, Lokpa HA: 0/2 crabs from three sites; Rassia HA: 0/4 crabs from one site), and the Loda River basin (Lokpa HA: 0/30 crabs from three sites) [18]. No crabs were found in 2015–2016 along the south-eastern Awo River near one of the 2009 capture points (Nyalebbe HA). Overall, no active crab-associated *S. neavei* breeding was detected in 2015–2016 at the prospected locations.

Regarding non-crab *Simulium* larval breeding habitats, between 2015 and 2017, larval and pupal prospections documented breeding of *S. dentulosum* and *S. vorax* at several basins: 1) Awo (Kakoi River basin) in Logo HZ, Thedeja HA*: S. dentulosum*, *S. vorax*; and Nyarambe HZ, Nyalebbe HA: *S. vorax*; 2) downstream Kakoi River basin in Nyarambe HZ, Afoyo HA: *S. vorax*; 3) Kuda River basin in Angumu HZ, Kudieweka HA: *S. vorax*; Logo HZ, Draju HA: *S. dentulosum*, *S. vorax*; and Nyarambe HZ, Kpanyi HA: *S. vorax*; and 4) Koda River basin in Rethy HZ, Lokpa HA: *S. dentulosum*; and Rassia HA: *S. dentulosum* [18]. Breeding of several non-anthropophagic *Simulium* species was also recorded [18].

**Adult female blackfly collections by informal “spot-check” HLCs**

Informal HLCs spot checks undertaken in August–November 2017 and February–April 2018 detected *Simulium (Anasolen) dentulosum* [65] and *S. vorax* Pomeroy [18,56,66] biting humans along the Kuda River basin across multiple HZs (HA): Angumu (*S. dentulosum* and *S. vorax* in Dabu), Logo (*S. vorax* in Draju), Nyarambe (*S. dentulosum* and *S. vorax* in Afoyo; and *S. dentulosum* in Kpanyi) and Rethy (*S. dentulosum* in Rassia).

A subset of the collected blackflies (155/161 *S. dentulosum* and 4/23 *S. vorax*) was processed individually for triplex real-time PCR (heads/bodies separated) to detect *O. volvulus* DNA. Among *S. dentulosum*, 30.3% were body-positive for *O. volvulus* (47/155; 95% CI: 23.3–38.3) and 11.0% were head-positive (infective; 17/155; 95% CI: 6.7–17.2). Four *S. dentulosum* flies were positive in both compartments. For *S. vorax*, 1/4 (25%) bodies and 0/4 (0%) heads were positive.

### 3.2 Geospatial analysis

An interactive ArcGIS web map aggregating epidemiological, entomological and land-cover layers (REMO/REA 2003–2015; skin-snip screening 2010–2011, 2015–2017, 2021–2023; anti-Ov16 surveys 2015–2021; informal HLC and breeding site prospections; forest loss 2001–2024; administrative boundaries; rivers/streams) is publicly available at https://arcg.is/1PDyO0. A README file is available in S1 Appendix Text A.

#### 3.2.1 Individual and spatial concordance of recent transmission indicators.

Paired anti-Ov16 RDT and skin-snip microscopy results were available for children aged 3–10 years from the 2015 epilepsy case-control [53] and 2017 epilepsy cohort [55] studies (n = 83). Agreement between tests was high by simple proportion (92.8%), while chance-corrected agreement was moderate (κ = 0.47, 95% CI: 0.11–0.82; −1 to 1 scale), consistent with low prevalence. There was no evidence that the tests differed in overall positivity rates (mid-p McNemar p = 0.125). Using skin-snip microscopy as reference, anti-Ov16 RDT in the field had 37.5% sensitivity (95% CI: 8.5–75.5) and 98.7% specificity (95% CI: 92.8–100.0).

Anti-Ov16 seroprevalence in children aged 3–10 years sampled from the community (2016–2021; including previously unreported data from the NSETHIO programme [30,31] provided in Table C in S1 Appendix and S1 Data) and adolescent (12–17 years) microfilaria-positive skin-snip prevalence in 2021–2023 (second moxidectin trial screening) showed a coherent, geographically structured pattern (see interactive map https://arcg.is/nb45e1). Positive surveys were clustered along north-eastern Muda River tributaries of the Kuda River basin, and north-western tributaries of the Lebu River basin in Draju and Kanga HAs (Logo HZ), with 6/110 children seropositive for anti-Ov16 IgG4 and 40/1,010 adolescents testing as microfilaria-positive by skin-snip microscopy. Adjacent Kuda River tributaries upstream of the Muda River showed additional low-positive signals, with children's serology yielding mixed results across nearby villages (4/127 seropositive), and adolescent microfilarial prevalence mirroring this pattern (27/908), producing a mosaic of zero to very low positivity in surveys conducted in Draju and along the borders with Ulyeko and Buu HA (Logo HZ).

In contrast, the north-eastern Kuda River branch (Kpanyi HA, Nyarambe HZ) showed no evidence of recent transmission, with 0/82 children seropositive and 0/357 adolescents skin-snip microfilaria-positive. Similarly, no evidence of recent transmission was detected in the surveyed locations in the Awo River basin or in the Kakoi River tributaries (including the sections of Suu and Ryeda rivers north of the Muda/Kuda), where only skin snips were undertaken (0/112; in Thedeja HA, Logo HZ).

Findings from the epilepsy case-control (2015) and the epilepsy cohort (2017) studies were consistent with this spatial pattern. Among 3–10-year-olds in the Kuda River basin (Draju), 6/35 skin snips and 3/30 anti-Ov16 tests were positive. Also, between the Koda and Loda River basins (Lokpa and Rassia HAs, Rethy HZ), 6/29 skin snips and 1/17 anti-Ov16 tests were positive. Together, these data indicate that field anti-Ov16 RDT results in children were broadly concordant with the spatial gradient observed for adolescent skin-snips.

#### 3.2.2 Landscape change and tree canopy loss.

Remote-sensing and field observations indicate substantial but heterogeneous tree-cover change across the Kakoi–Koda focus [67]. Agriculture (e.g., coffee, rice, tobacco and cotton), logging and conflict-related population movements have contributed to progressive deforestation, already documented in western South Ituri by the late 2000s [67]. Across the five HZs, 14,480 hectares met the dense forest definition (≥70% tree cover) in 2000, of which 95.7% lay in Rethy HZ (Table E in S1 Appendix). From 2001 to 2024, cumulative loss of all forest (≥30% tree cover) at the HZ level ranged from 3.4% to 7.1%, whereas loss of dense forest was 39.3% in Angumu, 62.6% in Logo, 72.5% in Mahagi, 84.9% in Nyarambe and 12.8% in Rethy. Dense forest loss was concentrated in 2001–2012 across Angumu, Logo, Mahagi and Nyarambe, with additional loss in 2017–2019 in Logo and in 2019 in Mahagi (Fig A in S1 Appendix). Rethy, which holds almost all the remaining dense forest of the focus, shows instead a progressive pattern of dense forest clearance that has accelerated since 2017.

At the HA level, dense forest loss was greatest in the HAs classified as hyperendemic by REMO (2003) or the 2010–2011 trial skin-snip population screening. Particularly, in Logo HZ, Draju and Kanga lost 89.6% and 74.8% of their 2000 dense forest by 2024, respectively. In Nyarambe HZ, Kpanyi lost 83.8%. The much smaller dense-forest remnants of Dabu (Angumu HZ), Afoyo (Nyarambe HZ) and the mesoendemic Pundiga HA (Nyarambe HZ) were cleared entirely. Losses were more modest in the other meso- and hyperendemic HAs, ranging from 1.9% in Aree to 39.4% in Langa (Angumu HZ) and 34.4% in Rassia (Rethy HZ). Health-area estimates for dense forest loss, with the 2000 baseline areas on which they are calculated, are given in Table E in S1 Appendix.

**Fig 4:**
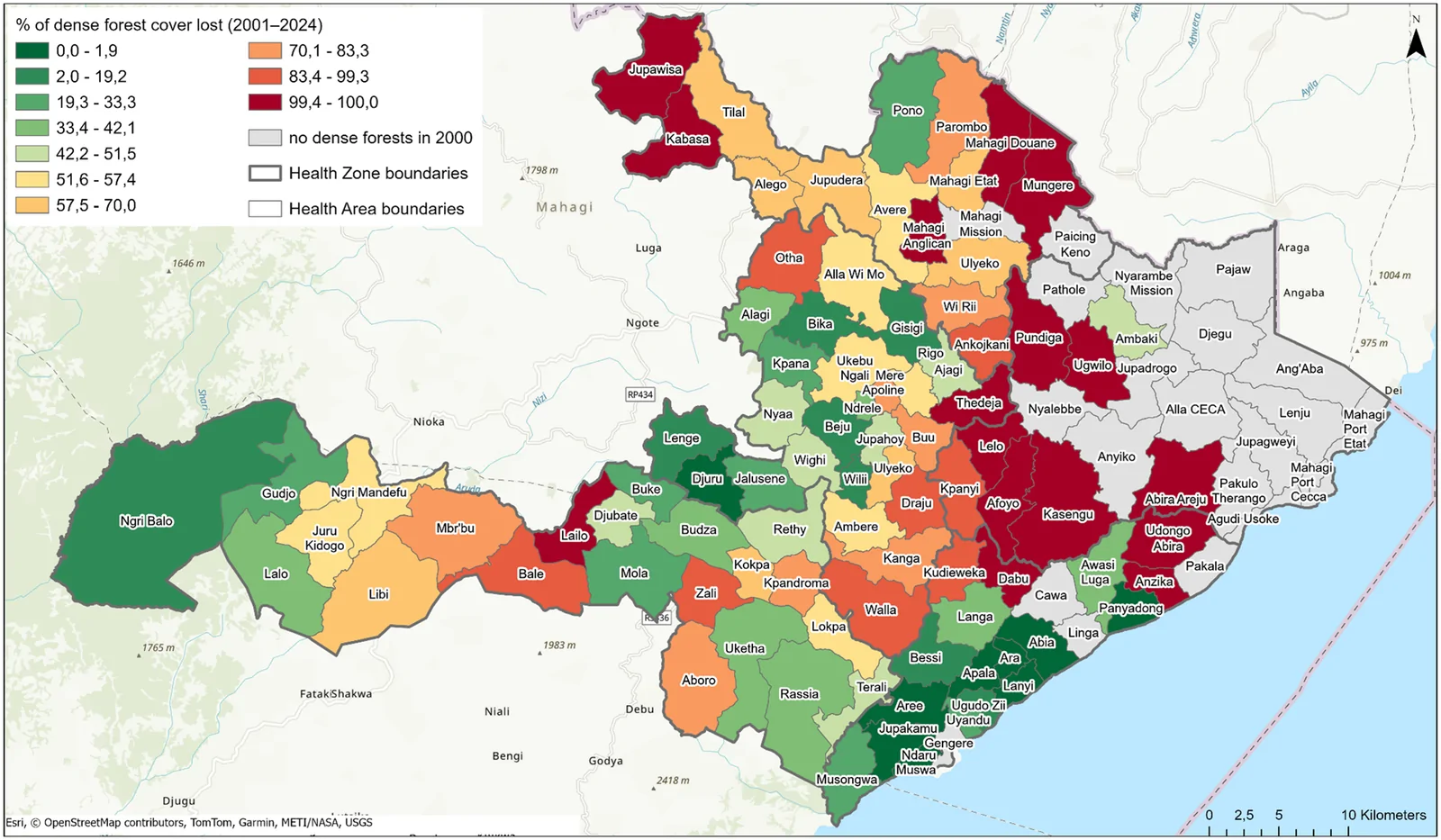
Percentage of dense forest cover lost between 2001 and 2024 in the Kakoi-Koda onchocerciasis focus. Health areas (black thin boundaries) are shaded by the percentage of dense forest (≥70% tree cover in 2000) that was lost by 2024, grouped into 10 quantile classes (from 0 to 100% loss). Health zone boundaries for Angumu, Logo, Mahagi, Nyarambe and Rethy are shown as bold grey lines. The map was produced in ArcGIS Pro (Esri, Redlands, CA, USA) using publicly available GRID3 COD – Health Areas v5.0 https://doi.org/10.7916/nnew-da26 [25] and GRID3 COD – Health Zones v5.0 https://doi.org/10.7916/d8rn-6e24 [26] boundaries and the Hansen Global Forest Change dataset (30-metre resolution; per cent tree cover in 2000 and annual tree loss, 2001–2024) [41,42].

At the river basin level, canopy loss was spatially heterogeneous across the focus (Table 3, Fig 4 and Fig B in S1 Appendix). The highest proportional losses were concentrated in the hyperendemic belt between Angumu, Logo and Nyarambe HZs (Fig 4), where dense forest has been extensively cleared (Fig B–F in S1 Appendix). This includes the Awo River basin (74% deforested; Table 3), the Muda River tributaries in the Kuda River basin (78% deforested), the northern Lebu River basin (92% deforested), and the northern Koda and Loda River basins (81% deforested). Small remnants of dense forest are located along the north-western Kakoi River tributaries (55% deforested; Fig G in S1 Appendix). Larger mosaics of dense forest persist in the mid- and downstream Koda and Loda River basins and around Mount Aboro (Rethy HZ), but together these have also lost about a third of their 2000 dense forest cover, particularly in riparian (river-bank) zones (Fig H in S1 Appendix).

**Table 3:** Dense forest (≥70% tree cover in 2000) and its cumulative loss by 2024, by river basin, in the Kakoi–Koda onchocerciasis focus. Figure references are to the corresponding maps in S1 Appendix.

River basin	Main health areas	Dense forest		
		In 2000 (hectares)	Lost 2001–2024 (hectares)	Lost (%)
**Awo** (Fig C)	Buu, Leo, Thedeja	35	26	74
**Muda tributaries, Kuda basin** (Fig D)	Draju, and borders with Ambere, Kanga, Kpanyi	69	54	78
**Northern Lebu** (Fig E)	Kudieweka, adjacent to Kanga	39	36	92
**Northern Koda and Loda** (Fig F)	Kpandroma–Walla–Lokpa boundaries	32	26	81
**North-western Kakoi tributaries** (Fig G)	Langa	11	6	55
**Mid- and downstream Koda** (Fig H)	Rassia	495	162	33
**Southern Loda** (Fig H)	Lokpa	19	10	53
**Mount Aboro reserve** (Fig H)	Uketha	21	3	14

**Note**: Hectare = 10,000 square meters.

## 4. Discussion

The Kakoi–Koda onchocerciasis focus in the DRC spans five HZs. Baseline nodule prevalence mapping (REMO, 2003) indicated hyperendemicity in parts of Logo, Nyarambe and Angumu HZs, with mesoendemicity in Rethy and possibly Mahagi HZs. Across independent data streams spanning two decades, onchocerciasis infection indicators in the focus have fallen sharply since the 2010–2011 (skin-snip) community screening for the Phase III moxidectin clinical trial [33,39], which at the time confirmed hyperendemicity in Logo and Nyarambe. Age (and sex) profiles of all-age anti-Ov16 surveys in 2015–2017 confirmed substantial cumulative exposure in parts of Logo and Rethy HZs, consistent with the baseline mapping.

By contrast, children-only (aged 3–10 years) anti-Ov16 seroprevalence from 2016 onward was uniformly low or zero in most surveyed HAs of Logo, Nyarambe and Rethy HZs that had been hyperendemic at baseline, with some positives mostly in older children (7–10 years), consistent with a reduction in the force of infection. Skin-snip screening corroborated this trajectory, with hyperendemic infection prevalence and intensity in 2010–2011 declining to hypoendemic levels by 2021–2023 in Draju and Kanga (Logo HZ), with the steepest relative declines in adolescents (the youngest group sampled using skin-snip microscopy). In Nyarambe (Kpanyi HA), where early sample sizes were small, prevalence in adults significantly fell from 72% to 3% in villages surveyed at both time points. In Rethy HZ, REMO categories changed from borderline hyperendemic (2003) to what would have been considered mesoendemic (2015), the latter result mirrored by microfilarial prevalence and intensity in studies among persons with epilepsy (2015). Spatially, residual transmission signals clustered along limited stretches of the Muda/Kuda and Lebu River basins (and possibly between Koda and Loda River basins). Other river basins, such as sections of Awo and Kakoi, showed no evidence of recent transmission.

The exact onset of the endemicity decline cannot be empirically dated due to the absence of earlier microfilarial data. However, none of the children aged 3–6 years tested in Draju (Logo HZ) in 2016 were anti-Ov16 seropositive (0/70). As these children were born approximately between 2010 and 2013, this finding is consistent with very limited transmission from around 2010 onwards. By 2021–2023, infection prevalence and intensity had dropped to low levels in previously hyperendemic HAs in Logo and Nyarambe HZs. In Logo, the evidence is consistent with sustained reductions in the force of infection for more than a decade.

The age-specific community anti-Ov16 seroprevalence profile observed in Logo HZ in 2016, with very low seroprevalence in young children, followed by a steep rise between 7 and 30 years of age and a plateau thereafter, is qualitatively similar to trajectories predicted for settings in which transmission has been substantially suppressed for one to two decades [68]. Although Logo HZ has not been under routine CDTI, analogous age patterns could result from a prolonged reduction in the force of infection driven by ecological change and vector species shifts and density declines. The marked decline in microfilarial prevalence and community infection intensity between the 2010–2011 and 2021–2023 moxidectin trial screenings are also broadly consistent with modelled trajectories for settings where transmission has declined over 15 years or more by 2021–2023 [68]. Transmission-modelling analyses using the microfilarial and anti-Ov16 seroprevalence data compiled here would be needed to test this quantitatively.

Vector ecology provides a plausible mechanism for changing transmission patterns [18]. Baseline meso- and hyperendemic onchocerciasis clusters at the Kakoi-Koda focus lay predominantly along escarpment slopes between higher and lower elevations, consistent with rapids suitable for *Simulium* breeding (see Fig 2 and relief model of the focus in [18]). The *Simulium neavei* group is phoretically associated with freshwater crabs and breeds in shaded streams, where its immature stages attach to crabs for transport and feeding [69]. While *S. neavei* is sensitive to canopy opening and habitat disturbance [46,70], *S. dentulosum* has been collected biting humans in the Ituri highlands and may act as a vector in more open habitats, with *S. vorax* possibly also contributing to transmission [18].

Historical information suggests that the *S. neavei* group occurred locally or in nearby Ituri rivers [71]. However, recent entomological work identified *Simulium dentulosum* and *S. vorax* as the anthropophagic species currently biting within the focus, with *O. volvulus* ND5-positive bodies (and for *S. dentulosum*, also heads), indicating parasite circulation along the Kuda River system in 2017–2018 [18]. In contrast, surveys since 2015 did not detect any active crab-associated *S. neavei* breeding in the prospected reaches (only one site with infested crabs was documented in Kakoi River in 2009) [18,56], consistent with its apparent disappearance from the focus. This pattern, together with progressive deforestation and canopy opening, is compatible with reduced shaded-stream habitat and disruption of crab-associated breeding, similar to the disappearance of *S. neavei* observed after bush-clearing in a hypoendemic focus in Nyanza Province, Kenya [72]. In several Ugandan onchocerciasis foci, the disappearance of *S. neavei* has likewise been attributed to deforestation [73]. These observations are consistent with the hypothesis proposed by Post *et al.* [18] of a shift in the local vector community from *S. neavei* to more open-habitat species (*S. dentulosum* and *S. vorax*), with lower overall vectorial density and/or capacity.

CDTI histories were heterogeneous across the focus. Angumu and Rethy HZs had long-running CDTI programmes for onchocerciasis since, respectively, 2009 and 2012, and Nyarambe HZ implemented mass drug administration of ivermectin and albendazole from 2016 for lymphatic filariasis, all programmes reporting high therapeutic coverage. In contrast, no CDTI was delivered in Logo or Mahagi HZs. Beyond routine delivery, microfilaricidal treatment had been provided to a small subset of residents recruited through moxidectin–ivermectin clinical trials (2010–2011; 2021–2026) [22,33] and an ivermectin trial among persons with epilepsy (2017–2018) [35,36]. Although the extent of these treatments was circumscribed rather than population-wide, enrolment often focused on individuals with higher microfilarial loads (to assess drug effects on infection intensity or seizure outcomes). Therefore, these targeted treatments may have contributed, to a limited extent, to the area-wide decline observed.

The marked onchocerciasis prevalence decline in Logo despite the absence of routine CDTI is compatible with a potential contribution from ecological drivers, particularly canopy loss along streams, which could have reduced suitable breeding habitat for *S. neavei* and constrained transmission to short river segments where anthropophagic *Simulium* species remain. Their vector competence and ability to maintain transmission are uncertain. The concordance between very low-to-absent anti-Ov16 seroprevalence in children, decreasing adolescent and adult infection prevalence and intensity, and the spatial contraction of transmission suggested by the evidence to the Muda/Kuda–Lebu rivers corridor (where CDTI has not been delivered) supports prioritising this corridor for further investigation. Future transmission modelling could account for both programmatic exposure (including the targeted trial doses) and ecological change when attributing the observed declines.

Two practical surveillance points emerge from our study. Firstly, anti-Ov16 RDT findings among children aged 3–10 years broadly reproduced the spatial gradient seen in skin snips, despite diagnostic limitations (limited sensitivity and RDT inability to distinguish ongoing from past infection) [58]. In our paired analysis of skin-snip microscopy and anti-Ov16 RDT in children, the field RDT showed low sensitivity (38%, 95% CI: 9–76) and high specificity (99%, 95% CI: 93–100) against skin-snip microscopy. A similar diagnostic performance range has been reported for the same field-based RDT in Burkina Faso among 400 children aged 3–9 years (sensitivity = 60% and specificity = 94%, when compared to skin-snip microscopy) [74] and for all ages in an ivermectin-naïve setting in Gabon (sensitivity = 52% and specificity = 95%, when compared to skin-snip microscopy for 4,257 participants aged ≥5 years) [75]. These estimates do not reach the WHO diagnostic Target Product Profile (TPP), which calls for very high specificity (≥99.8%) and more moderate sensitivity thresholds (≥60% for elimination mapping and ≥89% for stopping CDTI decisions) [76].

Secondly, REMO/REA nodule palpation remains helpful for delineating baseline endemicity, particularly in areas of moderate and high transmission, but is less sensitive in areas of low transmission or to indicate transmission declines than microscopy or serology [19]. For example, in Logo HZ, which never received CDTI, nodule prevalence in adult men remained in the meso- to hyperendemic range through 2017, whereas microfilarial prevalence in the same HAs (including the same villages) fell from 79.0% in 2010–2011 to 9.0% in 2021–2023. As adult worms survive for a decade or more [3,7] and the men examined in 2017 had median ages of 28–46 years, persistent nodules are compatible with worms acquired before the decline.

### 4.1 Limitations

We integrated programme histories, REMO/REA, study-based and community serological and skin-snip data, entomological observations and geospatial layers, enabling triangulation across designs and periods. This approach provides a comprehensive picture of the Kakoi–Koda focus but has several constraints. Firstly, several contributing studies were not designed to yield a population-representative sample of the focus. Most used pragmatic or study-specific enrolment criteria. For example, trial skin-snip screenings included participants aged ≥12 years, while case-control and epilepsy cohort studies used purposive sampling to investigate OAE rather than estimate population prevalence. Consequently, these studies may provide higher prevalence estimates than community-based surveys [55] and are interpreted as indicating where transmission may persist, rather than as estimates of population prevalence. Community serology surveys among children in Logo and Nyarambe HZs conducted in 2018 and 2021, respectively, provided complementary evidence of recent/ongoing *O. volvulus* transmission. We therefore emphasised patterns consistent across independent sources rather than individual study estimates.

Secondly, heterogeneity in study designs (trial screening, cross-sectional, case-control, cohort) and protocols (two versus four skin snips; microfilariae counted per snip or milligram of skin; differing age groups) limits precision and hampers direct comparisons of microfilarial intensity. The sensitivity of skin-snip microscopy increases with the number of snips and may miss light infections (e.g., in low-transmission or settings under CDTI), as suggested by modelling work [61]. Similarly, the Ov16 IgG4 RDT performed on whole blood has low and setting-dependent sensitivity [58,77]. To minimise bias, we restricted formal comparisons within study design groups, reported 95% CIs and ranges, and used exact tests (Fisher/Clopper-Pearson) when data were sparse. The trial-screening rounds also differed in sampling intensity and village composition. An individual-level, village-adjusted analysis of the trial-screening data has been published separately [22].

Thirdly, entomological findings are constrained by seasonal gaps, informal HLC procedures, limited PCR testing of *S. vorax* for *O. volvulus* larvae, and incomplete spatial coverage (notably across parts of Kakoi, Awo, Kuda/Muda and Lebu rivers), which limit the understanding of the capacity of these simuliid species to sustain *O. volvulus* transmission. Nevertheless, *S. dentulosum* samples were confirmed to indicate infectivity (*O. volvulus* DNA in heads) within the focus [18], and *S. vorax* has demonstrated competence for *O. volvulus* in laboratory settings [66] and can transmit *Onchocerca dukei* in cattle [18,78], indicating that both species remain epidemiologically relevant. Fourthly, tree-cover change was assessed from 30-metre imagery at a ≥70% canopy threshold. This measure captures the loss of forest blocks well, but only partially resolves the narrow riparian gallery forest most relevant to *S. neavei.*

Lastly, self-reported ivermectin intake in Kpanyi (very low among the moxidectin trial participants in 2021–2023) appears inconsistent with high CDTI coverage reports available in ESPEN for Nyarambe (80–84%) [21] and with the 2021 coverage among ivermectin-eligible children in Kpanyi (92%). While possible explanations include social desirability or reporting bias during screening and different sampling frames, this discrepancy warrants a targeted coverage verification survey.

### 4.2 Recommendations

Operationally, and in line with the WHO 2030 onchocerciasis elimination goal [1], risk-based surveillance should prioritise the Muda/Kuda and Lebu River basins and adjacent communities to determine whether CDTI should be initiated in Logo HZ and continued in the neighbouring HZs. Where ongoing transmission is verified, entomological monitoring should be considered to further characterise breeding sites, vector species, seasonal biting patterns, and infection/infectivity rates to guide interventions. Independent qualitative interviews with communities in Draju HA also identified the Kuda and Lebu lowland valleys as areas of highest current blackfly biting nuisance [31], independently highlighting these basins as priority areas for surveillance.

The assembled, standardised dataset accompanying this study may support transmission modelling to simulate prevalence changes in the focus [79]. In addition, blood-meal analysis could clarify host preferences of the current *Simulium* species found biting on humans and whether habitat change has altered human-vector contact, particularly in areas where flies may feed away from humans.

Although not population-representative, the case-control and cohort studies were informative for tracking epidemiological trends and, together with the recent community child surveys and second moxidectin skin-snip trial, helped identify areas where transmission may be reduced but not fully suppressed. Leveraging such local studies and other health programmes, for example, integrating anti-Ov16 RDTs or opportunistic skin-snip surveys into ongoing research or service platforms, may offer a pragmatic, interdisciplinary approach to strengthen NTD surveillance and optimise the limited logistical and financial resources available to tackle them, especially as external funding declines [80,81]. To maximise impact, datasets generated through such local studies and related health programmes should be shared openly to support planning and evaluation, as demonstrated in the depth of our analysis, which was possible only because previous data were accessible.

### 4.3 Conclusion

Epidemiological, entomological and geospatial evidence consistently indicate that *O. volvulus* infection indicators in the Kakoi–Koda focus have declined substantially since 2010, a pattern compatible with reduced transmission. The observations are compatible with reduced habitat suitability for *S. neavei* and possible shifts towards *S. dentulosum* and *S. vorax*. Programmatic effects (CDTI and small-scale trial treatments) may also have contributed to the observed declines when and where delivered. Whether the current vector species can maintain stable, endemic transmission remains uncertain, as current evidence suggests that any recent transmission may be limited and spatially fragmented. Targeted, integrated surveillance focused on the identified sub-basins should be prioritised before decisions on initiating, continuing or stopping CDTI and to support post-treatment surveillance. The standardised dataset assembled here provides a basis for future onchocerciasis transmission modelling. In operational terms, anti-Ov16 RDTs performed in the field broadly mirrored spatial patterns derived from skin-snip assessments, supporting cautious use as a complementary exploratory tool where the deployment of better diagnostics may not be feasible.

## Supporting information

S1 Data Supplementary dataset containing the epidemiological data used in the analyses for the Kakoi–Koda onchocerciasis focus. Published trial-screening, entomological and programme data are cited in the manuscript instead.
(CSV)

S1 Appendix Text A in S1 Appendix: ArcGIS README file for the interactive map of epidemiological, entomological and landscape data for the Kakoi–Koda onchocerciasis focus. **Text B in S1 Appendix:** Anti-Ov16 ELISA and concurrent skin-snip microscopy shared by ESPEN (context surrounding the Kakoi–Koda focus). F**ig A–H in S1 Appendix:** Annual dense forest loss (2001–2024) by health zone and basin-level maps of dense forest in 2000 and dense forest loss (2001–2024) across the Kakoi–Koda focus (Awo basin; Muda tributaries of the Kuda basin; northern Lebu basin; northern Koda and Loda basins; north-western Kakoi basins; southern Koda and Loda basins and Mount Aboro). **Table A in S1 Appendix:** Nodule palpation prevalence by health zone and health area, Kakoi–Koda focus (2003–2017). **Table B–D in S1 Appendix:** detailed all-age and child anti-Ov16 serology and two-skin-snip results retained as contextual evidence. **Table E in S1 Appendix:** Dense forest cover in 2000 and cumulative loss (2001–2024) by health area in the five health zones of the Kakoi–Koda focus.
(DOCX)
