# Supplementary material for: Declining Onchocerca volvulus transmission despite limited ivermectin delivery in the Kakoi–Koda focus, Ituri, Democratic Republic of the Congo: An epidemiological, entomological and landscape evidence synthesis
Source: PLoS Negl Trop Dis. 2026 Aug 31;20(8):e0014164. doi: 10.1371/journal.pntd.0014164 (PMC13557497; doi:10.1371/journal.pntd.0014164)
Supplement: Supplementary file 1 [file pntd.0014164.s002.docx]

Supporting information for: Declining *Onchocerca volvulus* transmission despite limited ivermectin delivery in the Kakoi–Koda focus, Ituri, Democratic Republic of the Congo: an epidemiological, entomological and landscape evidence synthesis

Luís-Jorge Amaral^1,2*^, Tony Ukety^3,4^, Jules Upenjirwoth^4^, Deogratias U Wonya’Rossi^4,5^, Michel Mandro^5^, Francoise Nyisi^4^, Pascal Adroba^4^, Wilma A. Stolk^6^, Joseph N. Siewe Fodjo^1^, María-Gloria Basáñez^2**^, Anne Laudisoit^7,8**^, Robert Colebunders^1**^

1. Global Health Institute, Faculty of Medicine and Health Sciences, University of Antwerp, Antwerpen, Belgium
2. UK Medical Research Council Centre for Global Infectious Disease Analysis, Department of Infectious Disease Epidemiology, School of Public Health, Imperial College London, London, United Kingdom
3. World Health Organization, Geneva, Switzerland
4. Centre de Recherche en Maladies Tropicales (CRMT), Rethy, Democratic Republic of Congo
5. Provincial Health Division Ituri, Ministry of Health, Bunia, Democratic Republic of Congo
6. Department of Public Health, Erasmus University Rotterdam, Rotterdam, The Netherlands
7. Evolutionary Ecology Group, Faculty of Sciences, University of Antwerp, Antwerpen, Belgium
8. Nature.Health.Global, New York, United States of America

* corresponding author: [luis-jorge.telesdemenesesdoamaral@uantwerpen.be](mailto:luis-jorge.telesdemenesesdoamaral@uantwerpen.be), [luis.amaral20@imperial.ac.uk](mailto:luis.amaral20@imperial.ac.uk) or [luisjtmamaral@gmail.com](mailto:luisjtmamaral@gmail.com)

^**^ contributed equally

**Table of Contents**

[Text A. ArcGIS README file for the interactive map of epidemiological, entomological and landscape data for the Kakoi-Koda onchocerciasis focus	3](#_Toc236650678)

[Table A. Nodule palpation prevalence by health zone and health area, Kakoi–Koda focus (2003–2017).	4](#_Toc236650679)

[Table B. Anti-Ov16 seroprevalence by health zone and health area (2015–2017), among residents aged ≥3 years and, in one survey, among men aged ≥20 years.	7](#_Toc236650680)

[Text B. Anti-Ov16 ELISA and concurrent skin-snip microscopy shared by ESPEN (context surrounding the Kakoi-Koda focus)	9](#_Toc236650681)

[Table C. Child (aged 3–10 years) anti-Ov16 seroprevalence by health zone and health area (2015–2021).	10](#_Toc236650682)

[Table D. Skin-snip results from other studies conducted in 2015–2017 by age group, health zone and health area.	12](#_Toc236650683)

[Figure A. Annual dense forest loss (2001–2024) across the five health zones of the Kakoi-Koda onchocerciasis focus.	14](#_Toc236650684)

[Figure B. Dense forest in 2000 and dense forest loss (2001–2024) across the Kakoi-Koda onchocerciasis focus, Ituri Province, DRC	15](#_Toc236650685)

[Figure C. Dense forest in 2000 and dense forest loss (2001–2024) in the Awo river basin (Kakoi-Koda focus)	16](#_Toc236650686)

[Figure D. Dense forest in 2000 and dense forest loss (2001–2024) in the Muda tributaries of the Kuda river basin (Kakoi-Koda focus)	17](#_Toc236650687)

[Figure E. Dense forest in 2000 and dense forest loss (2001–2024) in the northern Lebu river basin (Kakoi-Koda focus)	18](#_Toc236650688)

[Figure F. Dense forest in 2000 and dense forest loss (2001–2024) in the northern Koda and Loda river basins (Kakoi-Koda focus)	19](#_Toc236650689)

[Figure G. Dense forest in 2000 and dense forest loss (2001–2024) in the north-western Kakoi river basins (Kakoi-Koda focus)	20](#_Toc236650690)

[Figure H. Dense forest in 2000 and dense forest loss (2001–2024) in the southern Koda and Loda river basins and Mount Aboro (Kakoi-Koda focus)	21](#_Toc236650691)

[Table E. Dense forest cover in 2000 and cumulative loss (2001–2024) by health area in the 5 health zones of the Kakoi-Koda focus	22](#_Toc236650692)

[Table E. Continued	23](#_Toc236650693)

[Table E. Continued	24](#_Toc236650694)

[Table E. Continued	25](#_Toc236650695)

[References	26](#_Toc236650696)

# Text A. ArcGIS README file for the interactive map of epidemiological, entomological and landscape data for the Kakoi-Koda onchocerciasis focus

The map (available at <https://arcg.is/1PDyO0>) integrates epidemiological, entomological and landscape data used to analyse *Onchocerca volvulus* transmission dynamics in the Kakoi-Koda onchocerciasis focus, Ituri Province, Democratic Republic of the Congo. Layers are organised by survey year and data type to support exploration of spatial and temporal patterns and to enhance transparency of the analyses presented in the main article.

**The map displays:**

- Anti-Ov16 serological surveys (children, adolescents and adults)
- Skin-snip microscopy results (primarily adolescents and adults)
- REMO nodule prevalence surveys
- Blackfly human landing catches
- Blackfly larval and pupal breeding-site inspections, including freshwater crab prospections for *Simulium neavei*

Contextual layers include rivers and basins relevant to onchocerciasis transmission, Mount Aboro and surrounding topography, health-zone and health-area boundaries (GRID3 COD), historical land-use/land-cover (Landsat, 1980), and annual forest and dense-forest loss from the Global Forest Change dataset (2001–2024).

**Data sources and processing:** All datasets shown in the map were extracted from the epidemiological, entomological and programme sources listed in Table 1 of the main article, including published studies and data generated within the NSETHIO research programme reported here for the first time (available on S1 Dataset). Data were harmonised into a single master dataset with standardised variables, geographic identifiers and metadata prior to visualisation, published together with the main article.

**Privacy and access:** All data are aggregated at the village level and contain no personal identifiers. The map is publicly accessible and should not require specialised GIS software or institutional login credentials.

**Intended use:** The interactive map supports data exploration, interpretation of spatial concordance across data streams, and reproducibility of the findings presented in the accompanying publication.

# Table A. Nodule palpation prevalence by health zone and health area, Kakoi–Koda focus (2003–2017).

| **Health area** | **Study number, village^a^**  **[source of data]** | **Year** | **Study design; population** | **Number examined** | **Number with nodules** | **Nodule prevalence % (95% CI)** | **Endemicity class** |
| --- | --- | --- | --- | --- | --- | --- | --- |
| **Angumu Health Zone** | | | | | | | |
| Aree | 1, Jupanyarucel [1] | 2003 | REMO/REA; adults aged ≥20 years | 50 | 18 | 36.0 (23.3–50.9) | Mesoendemic |
| Bessi | 1, Bessi [1] | 2003 | REMO/REA; adults aged ≥20 years | 30 | 25 | 83.3 (64.5–93.7) | Hyperendemic |
| Dabu | 1, Dabu-Pajuma [1] | 2003 | REMO/REA; adults aged ≥20 years | 30 | 27 | 90.0 (72.3–97.4) | Hyperendemic |
| Langa | 1, Jupugworo [1] | 2003 | REMO/REA; adults aged ≥20 years | 30 | 21 | 70.0 (50.4–84.6) | Hyperendemic |
| Musongwa | 1, Musongwa-Mulio [1] | 2003 | REMO/REA; adults aged ≥20 years | 49 | 17 | 34.7 (22.1–49.7) | Mesoendemic |
| Ndaru Muswa | 1, Ndaru Muswa [1] | 2003 | REMO/REA; adults aged ≥20 years | 50 | 2 | 4.0 (0.7–14.9) | Non-endemic/sporadic |
| **Logo Health Zone** | | | | | | | |
| Draju | 1, Ndroy [1] | 2003 | REMO/REA; adults aged ≥20 years | 41 | 40 | 97.6 (85.6–99.9) | Hyperendemic |
|  | 2, Djupacora, Ndruli, Ruju [2] | 2015 | REA; men aged ≥20 years | 26 | 9 | 34.6 (17.9–55.6) | Mesoendemic^b^ |
|  | 3, Ndroy, Kpana [3] | 2017 | REA; men aged ≥20 years | 27 | 12 | 44.4 (26.0–64.4) | Hyperendemic^b^ |
|  | 4, 17 villages [4] | 2017 | Epilepsy cohort; adults aged ≥20 years living with epilepsy | 38 | 8 | 21.1 (10.1–37.8) | Not assigned^c^ |
| Kanga | 1, Kanga [1] | 2003 | REMO/REA; adults aged ≥20 years | 30 | 21 | 70.0 (50.4–84.6) | Hyperendemic |
|  | 3, Kanga, Jabi, Wi-Moo, Jupuleko, Nguu [3] | 2017 | REA; men aged ≥20 years | 37 | 21 | 56.8 (39.7–72.5) | Hyperendemic^b^ |
| Kpana | 1, Ndama [1] | 2003 | REMO/REA; adults aged ≥20 years | 30 | 0 | 0.0 (0.0–14.1) | Non-endemic/sporadic |
| Otha | 5 [5] | 2009 | REA; adults aged ≥20 years | 25 | 1 | 4.0 (0.2–22.3) | Non-endemic/sporadic |
| Thedeja | 1, Thedeja [1] | 2003 | REMO/REA; adults aged ≥20 years | 30 | 2 | 6.7 (1.2–23.5) | Hypoendemic |
| Ukebu Ngali | 1, Logo [1] | 2003 | REMO/REA; adults aged ≥20 years | 30 | 0 | 0.0 (0.0–14.1) | Non-endemic/sporadic |
| Walla | 3, Goma [3] | 2017 | REA; men aged ≥20 years | 30 | 6 | 20.0 (8.4–39.1) | Mesoendemic^b^ |
| **Mahagi Health Zone** | | | | | | | |
| Alego | 1, Luga [1] | 2003 | REMO/REA; adults aged ≥20 years | 30 | 0 | 0.0 (0.0–14.1) | Non-endemic/sporadic |
| Tilal | 1, Tilal [1] | 2003 | REMO/REA; adults aged ≥20 years | 35 | 1 | 2.9 (0.2–16.6) | Non-endemic/sporadic |
| **Nyarambe Health Zone** | | | | | | | |
| Abira Areju | 1, Panyadong [1] | 2003 | REMO/REA; adults aged ≥20 years | 30 | 0 | 0.0 (0.0–14.1) | Non-endemic/sporadic |
| Afoyo | 1, Jupa Lungu [1] | 2003 | REMO/REA; adults aged ≥20 years | 50 | 26 | 52.0 (37.6–66.1) | Hyperendemic |
| Nyalebbe | 1, Ukeru-Banda [1] | 2003 | REMO/REA; adults aged ≥20 years | 50 | 8 | 16.0 (7.6–29.7) | Hypoendemic |
| Nyarambe Mission | 1, Panyona-Angolyero [1] | 2003 | REMO/REA; adults aged ≥20 years | 30 | 1 | 3.3 (0.2–19.1) | Non-endemic/sporadic |
| Pundiga | 1, Pogi Jagi [1] | 2003 | REMO/REA; adults aged ≥20 years | 30 | 9 | 30.0 (15.4–49.6) | Mesoendemic |
| **Rethy Health Zone** | | | | | | | |
| Bale | 1, Rubu [1] | 2003 | REMO/REA; adults aged ≥20 years | 31 | 3 | 9.7 (2.5–26.9) | Hypoendemic |
| Lokpa | 2, Bala, Nyoka [2] | 2015 | REA; men aged ≥20 years | 29 | 5 | 17.2 (6.5–36.5) | Not assigned^c^ |
| Rassia | 1, Kpagboma [1] | 2003 | REMO/REA; adults aged ≥20 years | 30 | 11 | 36.7 (20.5–56.1) | Mesoendemic |
|  | 2, Gono 1, Ndeke 2, Upenji [2] | 2015 | REA; men aged ≥20 years | 54 | 16 | 29.6 (18.4–43.8) | Not assigned^c^ |
| **Rimba Health Zone** | | | | | | | |
| Uwilo | 1, Luga [1] | 2003 | REMO/REA; adults aged ≥20 years | 30 | 0 | 0.0 (0.0–14.1) | Non-endemic/sporadic |

**Note**: Nodule prevalence is shown with continuity-corrected Wilson score 95% confidence intervals. Endemicity classes follow the thresholds applied in the main text: hyperendemic ≥40%; mesoendemic ≥20% to <40%; hypoendemic ≥5% to <20%; non-endemic/sporadic <5%, the last corresponding to <10% microfilarial prevalence [6-8]. The 2003 REMO/REA surveys examined a median of 30 individuals (IQR: 30–40) aged ≥20 years per health area in a single village, of whom 82.7% (617/746) were male, with women examined where needed to reach the target sample size or higher confidence in the prevalence estimate. The 2015 and 2017 REAs examined men only, in line with the standard REA convention. Among participants with nodules in the 2015 REA, the median number of nodules was one (IQR: 1–1; range: 1–6). Among those with nodules in the 2017 REA, the median number was one (IQR: 1–1; range: 1–4).

**Abbreviations**: CI = confidence interval; OAE = onchocerciasis-associated epilepsy; REA = Rapid Epidemiological Assessment; REMO = Rapid Epidemiological Mapping of Onchocerciasis.

^a^ Study number is the numerical label (1–5) grouping rows belonging to the same data collection round; identical numbers indicate the same study conducted in multiple health areas. Study numbers are table-specific.

^b^ The 2015 and 2017 REAs sampled purposively in villages reporting a high prevalence of onchocerciasis-associated epilepsy, whereas the standardised REMO/REA surveys selected first-line villages by proximity to blackfly breeding sites. Both criteria are intended to identify the communities at highest risk within an area, but endemicity in villages with high epilepsy prevalence may yield higher estimates than standard REA site selection.

^c^ Endemicity class not assigned. The 2017 epilepsy cohort sampled persons with epilepsy rather than a community-based sample of adults, and the 2015 surveys in Rethy Health Zone are after the start of community-directed treatment with ivermectin (CDTI) there in 2012, whereas the REMO/REA endemicity thresholds were calibrated for pre-control settings.

# Table B. Anti-Ov16 seroprevalence by health zone and health area (2015–2017), among residents aged ≥3 years and, in one survey, among men aged ≥20 years.

| **Health Area** | **Study number [source of data]** | **Year** | **Study design; population** | **Median age (IQR; range)**  years | **Proportion of females** % | **Anti-Ov16 seropositive/total tested** | **Anti-Ov16 seroprevalence**  % **(95% CI)** |
| --- | --- | --- | --- | --- | --- | --- | --- |
| **Logo Health Zone** | | | | | | | |
| Draju | 1 [2, 9] | 2015 | Case-control; 56 epilepsy cases and 58 controls | 17 (12–25; 4–60) | 50.9 | 47/114 | 41.2 (32.2–50.8) |
|  | 2 [10] | 2016 | Cross-sectional; 438 community samples | 16 (9–35; 3–88) | 55.0 | 158/438 | 36.1 (31.6–40.8) |
|  | 3 [3] | 2017 | Cross-sectional; 27 men aged ≥20 years | 32 (24–47; 20–75) | 0.0 | 11/27 | 40.7 (23.0–61.0) |
|  | 4 [4] | 2017 | Epilepsy cohort; 73 samples | 21 (16–28; 3–59) | 53.4 | 47/73 | 64.4 (52.2–75.0) |
| Kanga | 2 [10] | 2016 | Cross-sectional; 476 community samples | 16 (8–34; 3–85) | 51.1 | 123/476 | 25.8 (22.0–30.1) |
|  | 3 [3] | 2017 | Cross-sectional; 37 men aged ≥20 years | 46 (33–58; 20–67) | 0.0 | 24/37 | 64.9 (47.4–79.3) |
| Ulyeko | 2 [10] | 2016 | Cross-sectional; 7 community samples | 28 (13–46; 3–48) | 28.6 | 2/7 | –^c^ |
| Walla | 3 [3] | 2017 | Cross-sectional; 30 men aged ≥20 years | 28 (23–37; 20–73) | 0.0 | 8/30 | 26.7 (13.0–46.2) |
| Mixed ^a^ | 4 [4] | 2017 | Epilepsy cohort; 286 samples | 20 (14–30; 3–72) | 48.3 | 80/286 | 28.0 (22.9–33.6) |
| **Rethy Health Zone** | | | | | | | |
| Lokpa | 1 [2, 9] | 2015 | Case-control; 24 epilepsy cases and 27 controls | 17 (12–28; 5–64) | 66.7 | 15/51 | 29.4 (17.9–44.0) |
| Rassia | 1 [2, 9] | 2015 | Case-control; 21 epilepsy cases and 16 controls | 15 (12–21; 7–55) | 31.6 | 14/37 | 37.8 (22.9–55.2) |

**Note:** Measured by field rapid diagnostic test. Direct statistical comparisons between studies should be avoided because study designs, sampling frames and age structures differ.

**Abbreviations**: CI = confidence interval; IQR = Interquartile range (1^st^ quartile to 3^rd^ quartile).

^a^ Most participants in the 2017 epilepsy cohort in Logo Health Zone (Kanga, Ulyeko, Thedeja and Walla Health Areas) lacked village information, so individual records could not be assigned to a specific health area.

^b^ Study number is the numerical label (1–4) grouping rows belonging to the same data collection round; identical numbers indicate the same study conducted in multiple health areas. Study numbers are table-specific.

^c^ Proportion and 95% confidence interval not reported for very small samples (number < 10).

# Text B. Anti-Ov16 ELISA and concurrent skin-snip microscopy shared by ESPEN (context surrounding the Kakoi-Koda focus)

The serological survey (anti-Ov16 ELISA) shared by ESPEN (Expanded Special Project for Elimination of Neglected Tropical Diseases) was conducted in 2015 in one village per health zone (HZ) across four HZs. The seroprevalence (95% confidence interval, CI) was: 1) Angumu HZ, Udongo Abira Health Area (HA): 5.2% (11/210; 95% CI: 2.8–8.4%) consistent with the nearby REMO classification of Abira Areju HA as non-endemic; 2) Mahagi HZ, Tilal HA: 0.3% (1/311; 95% CI: 0.0–2.1%), aligning with the REMO of Tilal as non-endemic; 3) Nyarambe HZ, Ang’Aba HA: 0.3% (1/302; 95% CI: 0.0–2.1%), confirmed to be beyond the Kakoi-Koda onchocerciasis focus limits according to REMO non-endemic results in Ang’Aba and Nyarambe Mission HAs; Rethy HZ, Libi HA: 27.1% (32/118; 95% CI: 19.5–36.2%) within the broader Ituri Sid CDTI programme, outside the Kakoi-Koda focus, and compatible with REMO hypoendemic classification in nearby Bale HA [1, 11, 12].

Across all sites, skin-snip microscopy was negative for *Onchocerca volvulus* (0/941). These findings support non-endemic status in the surveyed localities of Angumu, Mahagi and Nyarambe HZs. In Libi HA (Rethy HZ), serology indicates historical exposure of about a quarter of the tested population to *O. volvulus*, and the non-detectable patent infection at the time of survey (negative skin snips) likely reflects the impact of ongoing CDTI in that HZ.

# Table C. Child (aged 3–10 years) anti-Ov16 seroprevalence by health zone and health area (2015–2021).

| **Health Area** | **Study number^b^**  **[source of data]** | **Year** | **Study design; population** | **Median age**  **(IQR; range)** years | **Proportion of females** % | **Anti-Ov16 seropositive/total tested among 3–10-year-olds (3–6-year-olds subset in parentheses)** | **Anti-Ov16 seroprevalence among 3–10-year-olds**  % **(95% CI)** |
| --- | --- | --- | --- | --- | --- | --- | --- |
| **Logo Health Zone** | | | | | | | |
| Draju | 1 [2, 9] | 2015 | Case-control; 6 epilepsy cases and 20 controls | 8 (7–9; 4–10) | 43.8 | 3/26 (1/7) | 11.5 (3.0–31.3)^c^ |
|  | 2 [10] | 2016 | Cross-sectional; 138 community samples | 6 (4–8; 3–10) | 54.3 | 7/138 (0/70) | 5.1 (2.2–10.6) |
|  | 3 [4] | 2017 | Epilepsy cohort; 4 samples | 7 (5–8; 3–9) | 25 | 0/4 (0/2) | –^d^ |
| Kanga | 2 [10] | 2016 | Cross-sectional; 163 community samples | 6 (5–8; 3–10) | 47.2 | 7/163 (2/90) | 4.3 (1.9–9.9) |
|  | 4 [SD] | 2018 | Cross-sectional; 25 community samples | 8 (7–9; 5–10) | NA | 1/25 (0/4) | 4.0 (0.2–22.3)^c^ |
| Ulyeko | 2 [10] | 2016 | Cross-sectional; 2 community samples | 4 (3–5) | 50 | 0/2 (0/2) | –^d^ |
| Walla | 4 [SD] | 2018 | Cross-sectional; 35 community samples | 7 (5–9; 5–10) | NA | 0/35 (0/14) | 0.0 (0.0–16.6) |
| Mixed^a^ | 3 [4] | 2017 | Epilepsy cohort; 52 samples | 6 (4–8; 3–10) | 40.4 | 0/52 (0/28) | 0.0 (0.0–8.6) |
| **Nyarambe Health Zone** | | | | | | | |
| Kpanyi | 5 [SD] | 2021 | Cross-sectional; 95 community samples^e^ | 7 (7–9; 5–9) | 56.4 | 0/95 (0/1) | 0.0 (0.0–4.8) |
| **Rethy Health Zone** | | | | | | | |
| Lokpa | 1 [2, 9] | 2015 | Case-control; 3 epilepsy cases and 5 controls | 8 (7–8; 5–9) | 50 | 0/8 (0/2) | –^d^ |
| Rassia | 1 [2, 9] | 2015 | Case-control; 3 epilepsy cases and 6 controls | 7 (7–9; 7–9) | NA | 1/9 (–) | –^d^ |

**Note:** Measured by field rapid diagnostic test. Anti-Ov16 seroprevalence is shown with Wilson score 95% confidence intervals. Direct statistical comparisons between studies are not done, because study designs, sampling frames and age structures differ.

**Abbreviations**: CI = confidence interval; IQR = Interquartile range (1^st^ quartile to 3^rd^ quartile); NA = not available; SD = Survey data generated within the NSETHIO project and reported here for the first time [13] (see Methods [14] and data on S1 Dataset).

^a^ Most participants in the 2017 epilepsy cohort in Logo Health Zone (Kanga, Ulyeko, Thedeja and Walla Health Areas) lacked village information, preventing assignment to a specific health area.

^b^ Study number is the numerical label (1–5) grouping rows belonging to the same data collection round; identical study numbers indicate the same study conducted in multiple health areas. Study numbers are table-specific.

^c^ Small sample size (number = 10–30).

^d^ Proportion and 95% confidence interval not reported for very small samples (number < 10).

^e^ The 2021 community survey also tested two participants older than 10 years, who are not included in the 95 reported. Their ages were 14 and 18 years and both were seropositive.

# Table D. Skin-snip results from other studies conducted in 2015–2017 by age group, health zone and health area.

| **Health Area** | **Study number^b^; design (proportion females in %) [source of data]** | **Year** | **Number examined aged ≥18; 12–17; 7–11; 3–6 years** | **Skin snip microfilaria-positive/Total examined (prevalence %, 95% CI)** | | | | **Infection intensity overall (mf+ intensity; range)**  mf/skin snip |
| --- | --- | --- | --- | --- | --- | --- | --- | --- |
|  |  |  |  | **All ages** | **Aged 12–17 years** | **Aged 7–11 years** | **Aged 3–6 years** |  |
| **Logo Health Zone** | | | | | | | | |
| Draju | 1; case-control, 55 cases, 58 controls (51.1%^c^) [10] | 2015 | 43; 42; 21; 7 | 52/113 (46.0, 36.7–55.6) | 20/42 (47.6, 32.3–63.4) | 3/21 (14.3, 3.8–37.4^e^) | 1/7 (–^d^) | 18.3 (38.8; 0.5-352.0) |
|  | 2; case-control, 37 cases and 7 controls (54.5%) [2, 9] | 2015 | 28; 10; 3; 3 | 26/44 (59.1, 43.3–73.3) | 6/10 (60.0, 27.4–86.3^e^) | 1/3 (-^d^) | 1/3 (–^d^) | 92.6 (156.7; 1.0-1080.0) |
|  | 3; epilepsy cohort; 73 samples (53.4%) [4] | 2017 | 46; 21; 4; 2 | 46/73 (63.0, 50.9–73.8) | 11/21 (52.4, 30.3–73.6^e^) | 2/4 (-^d^) | 0/2 (–^d^) | 45.0 (71.4; 1.0-299.0) |
| Mixed^a^ | 3; epilepsy cohort; 322 samples (47.5%^c^) [4] | 2017 | 201; 74; 31; 16 | 98/322 (30.4, 25.5–35.8) | 20/74 (27.0, 17.7–38.8) | 2/31 (6.5, 1.1–22.8) | 0/16 (0.0, 0.0–24.1^e^) | 18.2 (59.9; 0.5-384.5) |
| **Rethy Health Zone** | | | | | | | | |
| Kpandroma | 2; case-control, 3 cases and 1 control (25%) [2, 9] | 2015 | 4; 0; 0; 0 | 0/4 (–^d^) | 0 | 0 | 0 | 0 |
| Lokpa | 1; case-control, 24 cases, 27 controls (66.7%^c^) [10] | 2015 | 24; 18; 7; 2 | 10/51 (19.6, 10.3–33.6) | 2/18 (11.1, 2.0–36.1^e^) | 1/7 (-^d^) | 0/2 (–^d^) | 2.4; (12.2; 0.5-59.5) |
|  | 2; case-control, 48 cases and 5 controls (52.8%) [2, 9] | 2015 | 34; 6; 9; 4 | 25/53 (47.2, 33.5–61.2) | 2/6 (–^d^) | 3/9 (-^d^) | 1/4 (–^d^) | 8.3; (17.5; 1.0-122.0) |
| Rassia | 1; case-control, 22 cases and 16 controls (35.0%^c^) [10] | 2015 | 16; 10; 12; 0 | 14/38 (36.8, 22.3–54.0) | 3/10 (30.0, 8.1–64.6^e^) | 4/12 (33.3, 11.3–64.6^e^) | 0 | 13.1; (35.4; 1.0-220.0) |

**Note:** Participants provided two skin snips each. *Onchocerca volvulus* microfilarial prevalence is shown with Wilson score 95% confidence intervals, and infection intensity as microfilariae per skin snip. Because these datasets are not community-based and paediatric strata were small, results are presented descriptively and should be interpreted cautiously within the broader epidemiological and entomological evidence from the Kakoi-Koda focus.

**Abbreviations**: CI = confidence interval; IQR = interquartile range (1^st^ quartile to 3^rd^ quartile); mf = microfilariae; mf + intensity = microfilarial load among those with microfilaria-positive skin snips (microfilariae per skin snip).

^a^ Most participants in the 2017 epilepsy cohort in Logo Health Zone (Kanga, Ulyeko, Thedeja and Walla Health Areas) lacked village information, preventing assignment to a specific health area.

^b^ Study number is the numerical label (1–3) grouping rows belonging to the same data collection round; identical study numbers indicate the same study conducted in multiple health areas. Study numbers are table-specific.

^c^ Many controls and some cases lacked sex information (21 controls and 2 cases in Draju, 20 controls and 1 case in Lokpa, and 16 controls and 2 cases in Rassia Health Areas).

^d^ Prevalence and 95% confidence interval not reported for very small samples (number < 10).

^e^ Small sample size (number = 10–30).

# Figure A. Annual dense forest loss (2001–2024) across the five health zones of the Kakoi-Koda onchocerciasis focus.

**Figure A. Annual dense forest loss (2001–2024) across the five health zones of the Kakoi–Koda onchocerciasis focus.** Annual loss corresponds to areas originally classified as dense forest in 2000 (≥70% tree cover) that were lost in each subsequent year according to the Hansen Global Forest Change dataset. Bar heights represent the area of dense forest lost per year (km²) within each health zone. Values are shown on free y-scales to highlight distinct temporal patterns across Angumu, Logo, Mahagi, Nyarambe and Rethy.

# Figure B. Dense forest in 2000 and dense forest loss (2001–2024) across the Kakoi-Koda onchocerciasis focus, Ituri Province, DRC

**Figure B. Dense forest in 2000 and dense forest loss (2001–2024) across the Kakoi-Koda onchocerciasis focus, Ituri Province, DRC.** Dense forest (≥70% tree cover) present in 2000 and still remaining to 2024 is shown in green, while dense forest lost between 2001 and 2024 is shown in red. Rivers and their names are indicated in blue. Health zone boundaries for Angumu, Logo, Mahagi, Nyarambe and Rethy are shown in bold grey. Mount Aboro is represented by a triangle. The location of the focus within the DRC is indicated by a black dot in the inset map in the top left corner. The map was produced in ArcGIS Pro (Esri, Redlands, CA, USA) using publicly available spatial datasets (GRID3 COD – Health Areas v5.0 <https://doi.org/10.7916/nnew-da26> [15] and GRID3 COD – Health Zones v5.0 <https://doi.org/10.7916/d8rn-6e24> [16]). River and stream networks were derived from OpenStreetMap (<https://www.openstreetmap.org>) [17] and adjusted using ground-truth waypoints.

# Figure C. Dense forest in 2000 and dense forest loss (2001–2024) in the Awo river basin (Kakoi-Koda focus)

**Figure C. Dense forest in 2000 and dense forest loss (2001–2024) in the Awo river basin (Kakoi-Koda focus).** Dense forest (≥70% tree cover) present in 2000 and still remaining to 2024 is shown in green (0.09 km^2^), while dense forest lost between 2001 and 2024 is shown in red (0.26 km^2^). Rivers and their names are indicated in blue. Health zone boundaries for Logo, Mahagi and Nyarambe are shown in bold grey. The map was produced in ArcGIS Pro (Esri, Redlands, CA, USA) using publicly available spatial datasets (GRID3 COD – Health Areas v5.0 <https://doi.org/10.7916/nnew-da26> [15] and GRID3 COD – Health Zones v5.0 <https://doi.org/10.7916/d8rn-6e24> [16]). River and stream networks were derived from OpenStreetMap (<https://www.openstreetmap.org>) [17] and adjusted using ground-truth waypoints.

# Figure D. Dense forest in 2000 and dense forest loss (2001–2024) in the Muda tributaries of the Kuda river basin (Kakoi-Koda focus)

**Figure D. Dense forest in 2000 and dense forest loss (2001–2024) in the Muda tributaries of the Kuda river basin (Kakoi-Koda focus).** Dense forest (≥70% tree cover) present in 2000 and still remaining to 2024 is shown in green (0.15 km^2^), while dense forest lost between 2001 and 2024 is shown in red (0.54 km^2^). Rivers and their names are indicated in blue. Health zone boundaries for Angumu, Logo, Nyarambe and Rethy are shown in bold grey. The map was produced in ArcGIS Pro (Esri, Redlands, CA, USA) using publicly available spatial datasets (GRID3 COD – Health Areas v5.0 <https://doi.org/10.7916/nnew-da26> [15] and GRID3 COD – Health Zones v5.0 <https://doi.org/10.7916/d8rn-6e24> [16]). River and stream networks were derived from OpenStreetMap (<https://www.openstreetmap.org>) [17] and adjusted using ground-truth waypoints.

# Figure E. Dense forest in 2000 and dense forest loss (2001–2024) in the northern Lebu river basin (Kakoi-Koda focus)

**Figure E. Dense forest in 2000 and dense forest loss (2001–2024) in the northern Lebu river basin (Kakoi-Koda focus).** Dense forest (≥70% tree cover) present in 2000 and still remaining to 2024 is shown in green (0.03 km^2^), while dense forest lost between 2001 and 2024 is shown in red (0.36 km^2^). Rivers and their names are indicated in blue. Health zone boundaries for Angumu and Logo are shown in bold grey. The map was produced in ArcGIS Pro (Esri, Redlands, CA, USA) using publicly available spatial datasets (GRID3 COD – Health Areas v5.0 <https://doi.org/10.7916/nnew-da26> [15] and GRID3 COD – Health Zones v5.0 <https://doi.org/10.7916/d8rn-6e24> [16]). River and stream networks were derived from OpenStreetMap (<https://www.openstreetmap.org>) [17] and adjusted using ground-truth waypoints.

# Figure F. Dense forest in 2000 and dense forest loss (2001–2024) in the northern Koda and Loda river basins (Kakoi-Koda focus)

**Figure F. Dense forest in 2000 and dense forest loss (2001–2024) in the northern Koda and Loda river basins (Kakoi-Koda focus).** Dense forest (≥70% tree cover) present in 2000 and still remaining to 2024 is shown in green (0.06 km^2^), while dense forest lost between 2001 and 2024 is shown in red (0.26 km^2^). Rivers and their names are indicated in blue. Health zone boundaries for Logo and Rethy are shown in bold grey. The map was produced in ArcGIS Pro (Esri, Redlands, CA, USA) using publicly available spatial datasets (GRID3 COD – Health Areas v5.0 <https://doi.org/10.7916/nnew-da26> [15] and GRID3 COD – Health Zones v5.0 <https://doi.org/10.7916/d8rn-6e24> [16]). River and stream networks were derived from OpenStreetMap (<https://www.openstreetmap.org>) [17] and adjusted using ground-truth waypoints.

# Figure G. Dense forest in 2000 and dense forest loss (2001–2024) in the north-western Kakoi river basins (Kakoi-Koda focus)

**Figure G. Dense forest in 2000 and dense forest loss (2001–2024) in the north-western Kakoi river basin (Kakoi-Koda focus).** Dense forest (≥70% tree cover) present in 2000 and still remaining to 2024 is shown in green (0.05 km^2^), while dense forest lost between 2001 and 2024 is shown in red (0.06 km^2^). Rivers and their names are indicated in blue. No health zone boundaries are shown as the map lies entirely within Angumu Health Zone. The map was produced in ArcGIS Pro (Esri, Redlands, CA, USA) using publicly available spatial datasets (GRID3 COD – Health Areas v5.0 <https://doi.org/10.7916/nnew-da26> [15] and GRID3 COD – Health Zones v5.0 <https://doi.org/10.7916/d8rn-6e24> [16]). River and stream networks were derived from OpenStreetMap (<https://www.openstreetmap.org>) [17] and adjusted using ground-truth waypoints.

# Figure H. Dense forest in 2000 and dense forest loss (2001–2024) in the southern Koda and Loda river basins and Mount Aboro (Kakoi-Koda focus)

**Figure H. Dense forest in 2000 and dense forest loss (2001–2024) in the Southern Koda and Loda river basins and Mount Aboro (Kakoi-Koda focus).** Dense forest (≥70% tree cover) present in 2000 and still remaining to 2024 is shown in green (3.33 km^2^ in southern Koda, 0.09 km^2^ in southern Loda and 0.18 km^2^ in Mount Aboro), while dense forest lost between 2001 and 2024 is shown in red (1.62 km^2^ in southern Koda, 0.10 km^2^ in southern Loda and 0.03 km^2^ in Mount Aboro). Rivers and their names are indicated in blue. Health zone boundaries for Angumu, Logo and Rethy are shown in bold grey. Mount Aboro is represented by a triangle. The map was produced in ArcGIS Pro (Esri, Redlands, CA, USA) using publicly available spatial datasets (GRID3 COD – Health Areas v5.0 <https://doi.org/10.7916/nnew-da26> [15] and GRID3 COD – Health Zones v5.0 <https://doi.org/10.7916/d8rn-6e24> [16]). River and stream networks were derived from OpenStreetMap (<https://www.openstreetmap.org>) [17] and adjusted using ground-truth waypoints.

# Table E. Dense forest cover in 2000 and cumulative loss (2001–2024) by health area in the 5 health zones of the Kakoi-Koda focus

| **Health Zone** | **Health Area** | **Dense forest in 2000 (m^2^)** | **Dense forest loss (m^2^)** | | | | | |
| --- | --- | --- | --- | --- | --- | --- | --- | --- |
|  |  |  | **2001-2005** | **2006-2010** | **2011-2015** | **2016-2020** | **2021-2024** | **Total** |
| Angumu | Abia | 11,576.4 | 0 | 0 | 0 | 0 | 0 | 0 |
| Angumu | Anzika | 2,315.4 | 2,310.0 | 0 | 0 | 0 | 0 | 2,310.0 |
| Angumu | Apala | 7,717.5 | 0 | 0 | 0 | 0 | 0 | 0 |
| Angumu | Ara | 6,945.8 | 0 | 0 | 0 | 0 | 0 | 0 |
| Angumu | Aree | 40,131.2 | 0 | 0 | 0 | 0 | 770.0 | 770.0 |
| Angumu | Awasi Luga | 12,348.1 | 2,310.0 | 1,540.0 | 0 | 770.0 | 0 | 4,620.0 |
| Angumu | Bessi | 63,283.8 | 5,390.0 | 770.0 | 1,540.0 | 0 | 0 | 7,700.0 |
| Angumu | Dabu | 6,945.8 | 2,310.0 | 4,620.0 | 0 | 0 | 0 | 6,930.0 |
| Angumu | Jupakamu | 120,393.5 | 0 | 0 | 0 | 0 | 0 | 0 |
| Angumu | Kudieweka | 337,256.2 | 148,609.0 | 95,479.4 | 76,229.5 | 0 | 0 | 320,317.8 |
| Angumu | Langa | 87,979.9 | 26,949.8 | 3,850.0 | 3,850.0 | 0 | 0 | 34,649.8 |
| Angumu | Lanyi | 1,543.5 | 0 | 0 | 0 | 0 | 0 | 0 |
| Angumu | Musongwa | 536,368.6 | 10,779.9 | 49,279.7 | 13,859.9 | 13,859.9 | 21,559.9 | 109,339.3 |
| Angumu | Ndaru Muswa | 1,543.5 | 0 | 0 | 0 | 0 | 0 | 0 |
| Angumu | Panyadong | 771.8 | 0 | 0 | 0 | 0 | 0 | 0 |
| Angumu | Udongo Abira | 10,804.6 | 10,009.9 | 770.0 | 0 | 0 | 0 | 10,779.9 |
| Angumu | Ugudo Zii | 28,554.9 | 0 | 0 | 1,540.0 | 2,310.0 | 1,540.0 | 5,390.0 |
| Angumu | Uyandu | 6,174.0 | 0 | 1,540.0 | 0 | 0 | 0 | 1,540.0 |
| **Angumu** | **Total** | 1,282,655 | 208,669 | 157,849 | 97,019 | 16,940 | 23,870 | 504,347 |
| Logo | Ajagi | 36,272.4 | 3,850.0 | 5,390.0 | 1,539.9 | 2,310.0 | 6,160.0 | 19,249.9 |
| Logo | Alagi | 35,500.7 | 6,930.0 | 4,620.0 | 2,310.0 | 0 | 0 | 13,859.9 |
| Logo | Alla Wi Mo | 856,646.2 | 97,019.3 | 66,989.5 | 45,429.7 | 264,108.2 | 10,780.0 | 484,326.7 |
| Logo | Ambere | 77,947.1 | 25,409.8 | 11,549.9 | 6,160.0 | 0 | 0 | 43,119.7 |
| Logo | Beju | 18,522.1 | 1,540.0 | 770.0 | 0 | 0 | 0 | 2,310.0 |
| Logo | Bika | 80,262.4 | 1,540.0 | 7,699.9 | 2,310.0 | 0 | 3,850.0 | 15,399.9 |
| Logo | Buu | 172,100.0 | 119,349.2 | 13,860.0 | 7,700.0 | 0 | 770.0 | 141,679.0 |
| Logo | Djuru | 26,239.6 | 0 | 0 | 0 | 0 | 0 | 0 |
| Logo | Draju | 319,505.9 | 217,138.5 | 30,029.8 | 29,259.8 | 6,930.0 | 2,310.0 | 285,668.1 |

# Table E. Continued

| **Health Zone** | **Health Area** | **Dense forest in 2000 (m^2^)** | **Dense forest loss (m^2^)** | | | | | |
| --- | --- | --- | --- | --- | --- | --- | --- | --- |
|  |  |  | **2001-2005** | **2006-2010** | **2011-2015** | **2016-2020** | **2021-2024** | **Total** |
| Logo | Gisigi | 104,186.7 | 3,850.0 | 5,390.0 | 0 | 770.0 | 0 | 10,009.9 |
| Logo | Jalusene | 20,065.6 | 770.0 | 1,540.0 | 3,850.0 | 0 | 0 | 6,160.0 |
| Logo | Jupahoy | 65,599.0 | 26,179.8 | 5,390.0 | 0 | 0 | 0 | 31,569.8 |
| Logo | Kanga | 64,827.3 | 27,719.8 | 7,700.0 | 8,469.9 | 1,540.0 | 3,080.0 | 48,509.7 |
| Logo | Kpana | 57,881.5 | 4,620.0 | 5,390.0 | 0 | 6,930.0 | 1,540.0 | 18,480.0 |
| Logo | Lenge | 86,436.4 | 1,540.0 | 770.0 | 0 | 1,540.0 | 0 | 3,850.0 |
| Logo | Mere Apoline | 192,938.3 | 20,019.9 | 25,409.8 | 13,859.9 | 91,629.4 | 1,540.0 | 152,459.0 |
| Logo | Ndrele | 47,076.0 | 3,850.0 | 1,540.0 | 2,310.0 | 9,239.9 | 1,540.0 | 18,479.9 |
| Logo | Nyaa | 154,350.7 | 0 | 16,169.9 | 0 | 61,599.6 | 1,540.0 | 79,309.5 |
| Logo | Otha | 572,640.0 | 378,837.4 | 80,079.5 | 14,629.9 | 56,979.6 | 0 | 530,526.4 |
| Logo | Rigo | 121,937.0 | 21,559.9 | 28,489.8 | 1,539.9 | 8,470.0 | 0 | 60,059.6 |
| Logo | Thedeja | 18,479.9 | 12,319.9 | 2,310.0 | 3,850.0 | 0 | 0 | 18,479.9 |
| Logo | Ukebu Ngali | 531,738.0 | 50,049.7 | 80,079.5 | 22,329.9 | 113,189.2 | 7,700.0 | 273,348.1 |
| Logo | Ulyeko | 92,610.4 | 50,049.7 | 7,700.0 | 6,930.0 | 770.0 | 0 | 65,449.7 |
| Logo | Walla | 210,688.7 | 63,909.6 | 22,329.9 | 90,859.4 | 3,080.0 | 0 | 180,178.8 |
| Logo | Wighi | 34,728.9 | 6,930.0 | 3,080.0 | 1,540.0 | 770.0 | 3,080.0 | 15,399.9 |
| Logo | Wilii | 30,098.4 | 0 | 2,310.0 | 0 | 0 | 0 | 2,310.0 |
| **Logo** | **Total** | 4,028,550 | 1,144,982 | 436,587 | 264,878 | 629,856 | 43,890 | 2,520,193 |
| Mahagi | Alego | 10,032.8 | 1,540.0 | 3,850.0 | 770.0 | 0 | 0 | 6,160.0 |
| Mahagi | Ankojkani | 104,958.5 | 26,949.8 | 64,679.6 | 11,549.9 | 0 | 770.0 | 103,949.3 |
| Mahagi | Avere | 52,479.2 | 5,390.0 | 3,080.0 | 5,390.0 | 16,169.9 | 0 | 30,029.8 |
| Mahagi | Jupawisa | 60,968.5 | 19,249.9 | 40,809.7 | 770.0 | 0 | 0 | 60,829.6 |
| Mahagi | Jupudera | 152,807.2 | 3,080.0 | 12,319.9 | 770.0 | 67,759.5 | 2,310.0 | 86,239.4 |
| Mahagi | Kabasa | 42,446.4 | 16,939.9 | 22,329.9 | 3,080.0 | 0 | 0 | 42,349.7 |
| Mahagi | Mahagi Anglican | 168,242.2 | 34,649.8 | 117,039.2 | 6,930.0 | 8,470.0 | 770.0 | 167,858.9 |
| Mahagi | Mahagi Douane | 771.8 | 0 | 770.0 | 0 | 0 | 0 | 770.0 |
| Mahagi | Mahagi Etat | 4,630.5 | 0 | 3,080.0 | 770.0 | 0 | 0 | 3,850.0 |
| Mahagi | Mungere | 771.8 | 770.0 | 0 | 0 | 0 | 0 | 770.0 |

# Table E. Continued

| **Health Zone** | **Health Area** | **Dense forest in 2000 (m^2^)** | **Dense forest loss (m^2^)** | | | | | |
| --- | --- | --- | --- | --- | --- | --- | --- | --- |
|  |  |  | **2001-2005** | **2006-2010** | **2011-2015** | **2016-2020** | **2021-2024** | **Total** |
| Mahagi | Parombo | 4,630.5 | 0 | 3,080.0 | 0 | 0 | 0 | 3,080.0 |
| Mahagi | Pono | 5,402.4 | 0 | 1,540.0 | 0 | 0 | 0 | 1,540.0 |
| Mahagi | Tilal | 9,261.0 | 0 | 6,159.0 | 0 | 0 | 0 | 6,159.0 |
| Mahagi | Ulyeko | 92,610.4 | 770.0 | 0 | 0 | 0 | 0 | 770.0 |
| Mahagi | Wi Rii | 3,858.8 | 2,310.0 | 770.0 | 0 | 0 | 0 | 3,080.0 |
| **Mahagi** | **Total** | 713,872 | 111,649 | 279,508 | 30,030 | 92,399 | 3,850 | 517,436 |
| Nyarambe | Abira Areju | 2,315.3 | 2,310.0 | 0 | 0 | 0 | 0 | 2,310.0 |
| Nyarambe | Afoyo | 1,543.5 | 770.0 | 770.0 | 0 | 0 | 0 | 1,540.0 |
| Nyarambe | Ambaki | 1,543.5 | 770.0 | 0 | 0 | 0 | 0 | 770.0 |
| Nyarambe | Kasengu | 2,315.3 | 770.0 | 1,540.0 | 0 | 0 | 0 | 2,310.0 |
| Nyarambe | Kpanyi | 213,775.7 | 84,699.4 | 28,489.8 | 47,739.7 | 14,629.9 | 3,080.0 | 178,638.8 |
| Nyarambe | Lelo | 2,315.3 | 2,310.0 | 0 | 0 | 0 | 0 | 2,310.0 |
| Nyarambe | Pundiga | 3,087.0 | 0 | 3,080.0 | 0 | 0 | 0 | 3,080.0 |
| Nyarambe | Ugwilo | 11,576.3 | 5,390.0 | 6,160.0 | 0 | 0 | 0 | 11,549.9 |
| **Nyarambe** | **Total** | 238,472 | 97,019 | 40,040 | 47,740 | 14,630 | 3,080 | 202,509 |
| Rethy | Aboro | 443,758.2 | 6,160.0 | 3,850.0 | 58,519.6 | 278,738.1 | 8,470.0 | 355,737.6 |
| Rethy | Bale | 12,348.1 | 5,390.0 | 2,310.0 | 3,850.0 | 0 | 0 | 11,549.9 |
| Rethy | Budza | 131,969.8 | 2,310.0 | 8,470.0 | 19,249.9 | 22,329.9 | 3,080.0 | 55,439.6 |
| Rethy | Buke | 4,630.5 | 0 | 770.0 | 0 | 770.0 | 0 | 1,540.0 |
| Rethy | Djubate | 1,543.5 | 770.0 | 0 | 0 | 0 | 0 | 770.0 |
| Rethy | Gudjo | 4,519,387.5 | 114,729.2 | 44,659.7 | 180,948.8 | 717,635.1 | 214,828.5 | 1,272,801.3 |
| Rethy | Juru Kidogo | 2,660,233.7 | 358,047.6 | 153,998.9 | 179,408.8 | 519,746.5 | 235,618.4 | 1,446,820.1 |
| Rethy | Kokpa | 8,489.3 | 3,080.0 | 2,310.0 | 0 | 0 | 0 | 5,390.0 |
| Rethy | Kpandroma | 102,643.2 | 30,029.8 | 42,349.7 | 7,700.0 | 3,080.0 | 0 | 83,159.4 |
| Rethy | Lailo | 10,804.6 | 5,390.0 | 5,390.0 | 0 | 0 | 0 | 10,779.9 |
| Rethy | Lalo | 12,565,687 | 229,458.4 | 236,388.4 | 382,687.4 | 1,896,497.0 | 1,532,290.0 | 4,277,320.8 |
| Rethy | Libi | 1,039,551.7 | 109,339.3 | 245,628.3 | 82,389.4 | 108,569.3 | 96,249.3 | 642,175.6 |
| Rethy | Lokpa | 262,396.1 | 31,569.8 | 15,399.9 | 80,849.5 | 11,549.9 | 770.0 | 140,139.0 |
| Rethy | Mbr'bu | 42,446.4 | 6,930.0 | 11,549.9 | 16,169.9 | 0 | 0 | 34,649.8 |

# Table E. Continued

| **Health Zone** | **Health Area** | **Dense forest in 2000 (m^2^)** | **Dense forest loss (m^2^)** | | | | | |
| --- | --- | --- | --- | --- | --- | --- | --- | --- |
|  |  |  | **2001-2005** | **2006-2010** | **2011-2015** | **2016-2020** | **2021-2024** | **Total** |
| Rethy | Mola | 16,978.6 | 3,080.0 | 0 | 770.0 | 0 | 0 | 3,850.0 |
| Rethy | Ngri Balo | 108,794,066 | 957,873.5 | 301,067.9 | 713,785.1 | 3,050,718.9 | 1,081,843.0 | 6,105,288.4 |
| Rethy | Ngri Mandefu | 2,232,682.4 | 84,699.4 | 87,009.4 | 564,406.2 | 344,187.7 | 147,069.0 | 1,227,371.6 |
| Rethy | Rassia | 4,538,681.3 | 332,637.7 | 174,018.8 | 165,548.9 | 800,794.5 | 89,319.4 | 1,562,319.3 |
| Rethy | Rethy | 226,123.7 | 46,969.7 | 10,779.9 | 13,089.9 | 21,559.9 | 11,549.9 | 103,949.3 |
| Rethy | Terali | 366,582.8 | 66,219.6 | 53,129.6 | 25,409.8 | 21,559.9 | 5,390.0 | 171,708.8 |
| Rethy | Uketha | 480,802.3 | 36,189.8 | 67,759.5 | 14,629.9 | 54,669.6 | 10,009.9 | 183,258.8 |
| Rethy | Zali | 71,773.1 | 54,669.6 | 4,620.0 | 4,620.0 | 770.0 | 0 | 64,679.6 |
| **Rethy** | **Total** | 138,533,580 | 2,485,543 | 1,471,460 | 2,514,033 | 7,853,176 | 3,436,487 | 17,760,699 |
| **Total** | | 144,797,129 | 4,047,862 | 2,385,444 | 2,953,700 | 8,607,001 | 3,511,176 | 21,505,184 |

**Note**: Health areas without dense forest in 2000 were not represented in this table: Mahagi Mission, Paicing Keno, Pathole, Nyarambe Mission, Pajaw, Djegu, Ang’Aba, Jupadrogo, Nyalebbe, Alla Ceca, Anyiko, Lenju, Jupagweyi, Mahagi Port Etat, Mahagi Port Cecca, Pakulo Theranog, Agudi Usoke, Pakala, Cawa, Linga and Gengere.

# References

1.	Ukety T, Ministère de la Santé Publique of République Démocratique du Congo, Centre Medical Evangélique. Projet d'ophtalmologie Aru: la cartographie de l'onchocercose dans les 7 zones de santé rurales au nord de district de l'Ituri. Aru: 2003.

2.	Levick B, Laudisoit A, Tepage F, Ensoy-Musoro C, Mandro M, Bonareri Osoro C, et al. High prevalence of epilepsy in onchocerciasis endemic regions in the Democratic Republic of the Congo. PLoS Negl Trop Dis. 2017;11(7):e0005732. Epub 20170714. doi: 10.1371/journal.pntd.0005732. PubMed PMID: 28708828; PubMed Central PMCID: PMCPMC5529017.

3.	Hotterbeekx A, Dusabimana A, Mandro M, Abhafule GM, Deogratias W, Siewe Fodjo JN, et al. Urinary N-acetyltyramine-O,β-glucuronide in persons with onchocerciasis-associated epilepsy. Pathogens. 2020;9(3). Epub 20200305. doi: 10.3390/pathogens9030191. PubMed PMID: 32151038; PubMed Central PMCID: PMCPMC7157704.

4.	Siewe Fodjo JN, Mandro M, Mukendi D, Tepage F, Menon S, Nakato S, et al. Onchocerciasis-associated epilepsy in the Democratic Republic of Congo: Clinical description and relationship with microfilarial density. PLoS Negl Trop Dis. 2019;13(7):e0007300. Epub 20190717. doi: 10.1371/journal.pntd.0007300. PubMed PMID: 31314757; PubMed Central PMCID: PMCPMC6663032.

5.	Lakwo T, Ukety T, Bakajika D, Tukahebwa E, Awaca P, Amazigo U. "Cross-border collaboration in onchocerciasis elimination in Uganda: progress, challenges and opportunities from 2008 to 2013". Global Health. 2018;14(1):16. Epub 20180206. doi: 10.1186/s12992-018-0333-1. PubMed PMID: 29409509; PubMed Central PMCID: PMCPMC5801695.

6.	World Health Organization, African Programme for Onchocerciasis Control. Guidelines for revising ivermectin treatment boundaries within the context of onchocerciasis elimination 2015 [cited 2026 9th of March]. Available from: <https://iris.who.int/handle/10665/343029>.

7.	Zouré HG, Noma M, Tekle AH, Amazigo UV, Diggle PJ, Giorgi E, et al. The geographic distribution of onchocerciasis in the 20 participating countries of the African Programme for Onchocerciasis Control: (2) pre-control endemicity levels and estimated number infected. Parasit Vectors. 2014;7:326. Epub 20140722. doi: 10.1186/1756-3305-7-326. PubMed PMID: 25053392; PubMed Central PMCID: PMCPMC4222889.

8.	O'Hanlon SJ, Slater HC, Cheke RA, Boatin BA, Coffeng LE, Pion SD, et al. Model-based geostatistical mapping of the prevalence of *Onchocerca volvulus* in West Africa. PLoS Negl Trop Dis. 2016;10(1):e0004328. Epub 20160115. doi: 10.1371/journal.pntd.0004328. PubMed PMID: 26771545; PubMed Central PMCID: PMCPMC4714852.

9.	Mandro M, Suykerbuyk P, Tepage F, Rossy D, Ngave F, Hasan MN, et al. *Onchocerca volvulus* as a risk factor for developing epilepsy in onchocerciasis endemic regions in the Democratic Republic of Congo: a case control study. Infectious Diseases of Poverty. 2018;7(1):79. doi: 10.1186/s40249-018-0465-9.

10.	Lenaerts E, Mandro M, Mukendi D, Suykerbuyk P, Dolo H, Wonya'Rossi D, et al. High prevalence of epilepsy in onchocerciasis endemic health areas in Democratic Republic of the Congo. Infect Dis Poverty. 2018;7(1):68. Epub 20180801. doi: 10.1186/s40249-018-0452-1. PubMed PMID: 30064504; PubMed Central PMCID: PMCPMC6069757.

11.	Ngave FN, Wonyarossi DU, Abhafule GM, Mande JL, Nyathirombo A, Uvon CB, et al. *Onchocerca volvulus* infection prevalence and intensity in Logo and Nyarambe Health Zones in Ituri, Democratic Republic of the Congo in 2010 and in 2021-2023: results of screening for clinical trials of moxidectin versus ivermectin. Parasit Vectors. 2026;19(1). Epub 20260316. doi: 10.1186/s13071-025-07199-8.

12.	World Health Organization. ESPEN: Democratic Republic of the Congo ESPEN: WHO; 2025 [cited 2026 9th of March]. Available from: <https://espen.afro.who.int/maps-data/countries/democratic-republic-congo#onc--data>.

13.	Geelhand de Merxem D, Siewe Fodjo JN, Menon S, Hotterbeekx A, Colebunders R. Nodding syndrome research, lessons learned from the NSETHIO project. Glob Ment Health (Camb). 2019;6:e26. Epub 20191018. doi: 10.1017/gmh.2019.24. PubMed PMID: 31807310; PubMed Central PMCID: PMCPMC6880249.

14.	Laudisoit A, Mandro Ndahura M, Kipute D, Tepage F, Ukety T, Post R, et al. The *Onchocerca* vectors of Ituri: an old mystery to solve. Report. 2018.

15.	Center for Integrated Earth System Information Columbia University, Ministère de la Santé Publique of Democratic Republic of the Congo. GRID3 COD - Health Areas v5.0 New York: Columbia University2025 [cited 2026 9th of March]. Available from: <https://doi.org/10.7916/8e06-tn49>.

16.	Center for Integrated Earth System Information Columbia University, Ministère de la Santé Publique of Democratic Republic of the Congo. GRID3 COD - Health Zones v5.0 New York: Columbia University2025 [cited 2026 9th of March]. Available from: <https://doi.org/10.7916/7se1-py22>.

17.	OpenStreetMap contributors. OpenStreeetMap African waterways 2021 [cited 2026 9th of March]. Available from: <https://www.openstreetmap.org>.
