# Supplementary material for: Declining Onchocerca volvulus transmission despite limited ivermectin delivery in the Kakoi–Koda focus, Ituri, Democratic Republic of the Congo: An epidemiological, entomological and landscape evidence synthesis
Source: PLoS Negl Trop Dis. 2026 Aug 31;20(8):e0014164. doi: 10.1371/journal.pntd.0014164 (PMC13557497; doi:10.1371/journal.pntd.0014164)
Supplement: Supplementary file 2 [file pntd.0014164.s003.docx]

Dear Editor,

As part of the peer review process, I have reviewed the manuscript entitled "Epidemiological, vectorial and landscape changes in the context of declining *Onchocerca* *volvulus* transmission across the Kakoi-Koda focus, Ituri, Democratic Republic of the Congo" (Manuscript PNTD-D-26-00524). It is a synthesis work, bringing together epidemiological, entomological, programme-related and geospatial data from a historical onchocerciasis focus in the DRC over two decades. The triangulation of evidence and the public release of data are notable strengths.

However, the manuscript has several methodological and stylistic weaknesses that, in my opinion, prevent it from being accepted in its current form. I therefore recommend major revisions. Below is a summary of the critical points that must be addressed section by section:

**Title**

The key discovery is the transmission decline without CDTI, and the title does not highlight this. The authors should rephrase this title to make it more informative

**Abstract section**

The background does not state the specific knowledge gap for Kakoi-Koda. Why do you conduct this study.

Line 31: In the methodology, “we synthesized” is too vague. State clearly that this is a scoping review

Line 45-46: The authors stated an overly confident causal statement: “Progressive deforestation…” change to “Progressive deforestation and canopy opening are consistent with a shift from crab-associated S. neavei to more open-habitat vectors, providing a plausible ecological mechanism."

**Author summary**

Line 62-64: "This decline appears linked to..." is again too causal. Change to: "This decline appears to be associated with..."

**Introduction section**

Line 111-113 and line 118-126: The authors should move the detailed trial descriptions to a new subsection in methods (“Clinical trial contexts").

Line 96-103: The authors describe CDTI expansion in DRC, which is only tangentially relevant to the Kakoi-Koda focus. They should condense this part to one sentence.

In the last paragraph the authors present a descriptive aim (“we synthesize… and relate”), not testable hypothesis. The authors should reframe this last paragraph and state specifics hypotheses.

**Methods section**

The authors should state in this section that most data sources are not population-representative and that results should be interpreted as descriptive of individuals sample, not necessarily in the general population. The authors should add a strong caveat like “due to the small number of skin-snip positive children, the sensitivity estimate is imprecise and should be interpreted with extreme caution.

Standardized HLC protocols have existed for decades. "Informal spot-checks" are non-reproducible and highly biased. Entomologic data should be removed or heavily caveated. Furthermore, opportunistic collections with tiny sample sizes cannot support quantitative conclusions about vector species or infection rates.

Line 601: Why were only 4/23 *S. vorax* tested? This needs justification. The prevalence estimate (1/4 = 25%) is statistically meaningless.

Considering the different studies for skin snip in this article, two vs four skin snips is a major issue for comparing intensities. The authors mention this in Discussion, but it should be stated as a limitation in methods and handled more rigorously (e.g., only compare prevalences, not intensities, between the two protocols).

Line 651-669: The paragraph with multiple percentages is indigestible. The authors should create supplementary table with all HA-level forest cover and loss percentages and summarize key patterns only in the text.

**Statistical analysis section**

Comparison of prevalence estimates between 2009-2011 and 2021-2023 by looking at whether confidence intervals overlap is a weak descriptive approach. A mixed-effects logistic regression with village as random effect and survey period as fixed effect would quantify the decline while accounting for clustering.

The authors compare prevalence estimates between Table 4 (4 snips, mf/mg) and Table 5 (2 snips, mf/snip). This is invalid due to protocol heterogeneity.

**Discussion**:

Line 764-765: The hypothesis “sociopolitical instability and immigration of relatively wealthier households who are less engaged in…” is not supported by any data presented in the manuscript. The authors should remove it.

Line 781-785: "The marked onchocerciasis prevalence decline in Logo... points towards a substantial role for ecological drivers…" This is correlation, not causation. The authors do not have no historical entomological data on S. neavei abundance before deforestation. They should revise causal language.

**Conclusion section**

In this section, the authors claim the RDT is useful and supports its use. However, with 43% sensitivity, the RDT would miss more than half of truly infected children. The authors should revise this conclusion about anti-Ov16 RDTs

**Limitations**:

There is no mention of the "informal spot-check" HLCs as a limitation and potential recall bias in self-reported ivermectin intake.
