# Supplementary material for: Declining Onchocerca volvulus transmission despite limited ivermectin delivery in the Kakoi–Koda focus, Ituri, Democratic Republic of the Congo: An epidemiological, entomological and landscape evidence synthesis
Source: PLoS Negl Trop Dis. 2026 Aug 31;20(8):e0014164. doi: 10.1371/journal.pntd.0014164 (PMC13557497; doi:10.1371/journal.pntd.0014164)
Supplement: Supplementary file 3 [file pntd.0014164.s004.docx]

**Review report of the manuscript entitled: Epidemiological, vectorial and landscape changes in the context of declining Onchocerca volvulus transmission across the Kakoi-Koda focus, Ituri, Democratic Republic of the Congo**

This article presents a well-conceived and valuable synthesis of epidemiological, entomological and geospatial evidence documenting the marked decline of Onchocerca volvulus transmission in the Kakoi-Koda focus in DRC. The study fills an important gap in knowledge about onchocerciasis transmission dynamics in Central Africa and has clear operational significance for elimination programming in the DRC. However, several issues require attention before the manuscript can be accepted.

**Major comments:** About the entomological indicators

Given the infection high infection rates, the most consequential operational question posed by this manuscript is whether current vector species found by the team can sustain transmission above elimination thresholds, is left largely unanswered, and the analysis presented does not fully exploit the available entomological data. Considering the following point:

1. The PCR head-positivity rate in S. dentulosum of 11.0% is very high and represents the best available proxy for vectorial infectivity in the focus. However, the authors do not contextualize this rate relative to published estimates of Annual Transmission Potential (ATP) thresholds for elimination. Without biting rate data, the ATP cannot be estimated, and the authors rightly acknowledge this limitation. However, the Discussion should explicitly state what head positivity rates and biting densities would be needed to reach or exceed the ATP threshold, so the reader can appreciate the magnitude of the uncertainty. A brief quantitative illustration (even a range based on published biting rate data from comparable highland foci in East Africa) would substantially strengthen the interpretation.
2. The HLC data were collected as informal 30 to 60 minutes spot checks, with no standardized collection protocol, no parity dissections, no daily or seasonal replication, and no formal biting rate estimation. The entomological findings should therefore be described explicitly as qualitative indicators of vector presence and infection status, rather than as quantitative transmission estimates. The current framing in the discussion (lines 762 to 764, "lower overall vectorial density and/or capacity") implies quantitative conclusions that the data cannot support.

**Major comment 2:** Confounding in the cross-zone comparison (Logo vs Nyarambe)

The comparison between CDTI-naïve Logo HZ (mf prevalence declining from 74% to 9%) and Nyarambe HZ (receiving CDTI from 2016 where mf declined from 72% to 3%) is the pivotal evidence for ecological rather than programmatic drivers of transmission decline, but the manuscript presents it without adequately addressing the major sources of confounding.

1. The two health zones are geographically distinct, occupy different positions along the river systems, and may differ in baseline vector density, vector species composition, and landscape context. The differences forest canopy loss between Logo and Nyarambe HZs ( 62.4% compared 85.1% dense forest loss respectively) suggest that Nyarambe may have experienced even greater habitat disruption. It is therefore plausible that ecological change alone (without CDTI) could account for part of the decline in Nyarambe as well, undermining the contrast with Logo.
2. The parallel decline in Logo (CDTI-naïve) and Nyarambe (CDTI from 2016) should be presented as evidence that ecological change, rather than CDTI, is the dominant driver of transmission decline. However, given that skin-snip data were collected in 2021–2023 (five years after CDTI began in Nyarambe), the CDTI contribution in Nyarambe cannot be dismissed. The Discussion acknowledges this (in line 780) but should be more explicit about what the data can and cannot establish causally.

**Minor comments**

**Abstract**

Lines 38 to 39. The Abstract states microfilarial prevalence in CDTI-naïve Logo fell from 69-79% to 9%. The range 69-79% refers to different HAs (Draju 79% and Kanga 69%), which is not immediately clear to a reader of the Abstract without reference to the main text. Consider specifying Logo villages or presenting a pooled prevalence to improve clarity.

**Introduction**

In line 90, the text lists "nodding syndrome" as an example of neurological sequelae. While the association between nodding syndrome and onchocerciasis remains debated, the current framing presents it as established epidemiological fact. Please add appropriate qualifying language (for example “including nodding syndrome, for which an association with onchocerciasis has been proposed”) or cite a reference that addresses this ongoing scientific debate.

**Methods**

Evidence synthesis (lines 190 to 196): The PRISMA-ScR framework was used for the scoping review, but the manuscript does not include the PRISMA-ScR checklist as a supplementary item. Additionally, please clarify whether double data extraction was performed. The methods state that discrepancies were resolved by discussion with co-authors, but do not indicate whether data were initially extracted independently by two reviewers.

Lines 293-295 state that Nyarambe received CDTI for LF from 2016, not for onchocerciasis. This is a crucial distinction: LF-directed MDA with ivermectin + albendazole may have had collateral effects on onchocerciasis, but community-level coverage and frequency may differ from onchocerciasis-directed CDTI. Please more explicitly discuss whether the LF MDA in Nyarambe is expected to have had similar effects on O. volvulus as onchocerciasis-directed CDTI, particularly given similar ivermectin doses and coverage levels.

Line 342: onchocercomata is the correct technical term but may benefit from a brief parenthetical definition for non-specialist readers.

In lines 469 – 475 (Comparison of intensity metrics). The text correctly states that mf intensity is expressed as mf/mg skin in the moxidectin trial data and as mf/skin snip in the OAE studies, and that these are not compared. However, a brief explanatory sentence clarifying why conversion between metrics is not straightforward (for example, snip weights were not recorded in the OAE studies or the Holth punch used yields snips of variable weight) would help readers unfamiliar with this technical distinction.

Lines 601 to 602 about the Subset of blackflies tested by PCR: 155/161 S. dentulosum and 4/23 S. vorax were processed for PCR. Please explain in the Methods or Results why 6 S. dentulosum and 19 S. vorax were not tested (insufficient material, damaged specimens, selective sampling). If testing was selective, please clarify whether this could have introduced bias into the reported PCR positivity rates.
