# Supplementary material for: Declining Onchocerca volvulus transmission despite limited ivermectin delivery in the Kakoi–Koda focus, Ituri, Democratic Republic of the Congo: An epidemiological, entomological and landscape evidence synthesis
Source: PLoS Negl Trop Dis. 2026 Aug 31;20(8):e0014164. doi: 10.1371/journal.pntd.0014164 (PMC13557497; doi:10.1371/journal.pntd.0014164)
Supplement: Supplementary file 4 [file pntd.0014164.s005.docx]

**Response to the Editor and Reviewers**

**“Declining *Onchocerca volvulus* transmission despite limited ivermectin delivery in the Kakoi–Koda focus, Ituri, Democratic Republic of the Congo: an epidemiological, entomological and landscape evidence synthesis”**

Dear Editor and Reviewers,

We are grateful for the constructive and detailed reviews. The comments have substantially improved the clarity and structure of the manuscript, and we have revised it accordingly. Each comment is reproduced below in italics and followed by our response.

We are submitting a version with tracked changes alongside a clean version.

**Reviewer 1:**

**Comment 1**

*While the authors do a good job of avoiding inappropriate statistical comparisons, the net result is a very lengthy paper that is hard to read and extract the key findings. There aren't a lot of analyses in this paper, it is really a synthesis of existing data.*

1. **Response:**

We are grateful for this assessment. The manuscript has been reorganised around the directly comparable repeated cross-sectional four-skin-snip trial-screening datasets, which now constitute the main formal comparison, against the REMO baseline. Serology, two-skin-snip studies, entomological observations and geospatial analyses are explicitly designated as further contextual evidence and are presented far more concisely. Table 1 is retained in the main article as the methodological overview of the evidence base, while the detailed heterogeneous tables have been moved to S1 Appendix as Tables A to D.

**Comment 2**

*The ArcGIS interactive map is a useful feature and a nice product to come from this paper. I don't find Table 3 very useful because of the drastically different sampling approaches (e.g. case-control vs. cross-sectional vs. epilepsy cohort) and differing sample sizes (ranging from 4 - 163).*

2. **Response**

We thank the reviewer for the encouraging remark about the interactive map, and we agree regarding the former Table 3. The heterogeneous designs and very small samples in some strata make the former Table 3 less useful to keep in the main article. We therefore moved it to S1 Appendix as Table B and retained only the principal descriptive pattern in the main text.

**Comment 3**

*The article is written very clearly - the prose is excellent - it is just too long and lacks cohesion.*

3. **Response**

We appreciate this distinction and have tried to preserve the prose while addressing the structural problem. Repetitive descriptions have been shortened, and the Introduction, Methods, Results and Discussion now follow a consistent order across data streams (programme delivery 🡪 infection indicators 🡪 vectors 🡪 landscape). The final Introduction paragraph states the knowledge gap, the three specific objectives and the exploratory nature of the synthesis.

**Comment 4**

*This study seeks to synthesize data on onchocerciasis (both in humans and vectors) in the Kakoi-Koda focus in DRC. While such an effort is laudable and the authors clearly did a lot of work to compile all the information, it is difficult for the reader to digest as presented. The data include a mix of geographic locations, time points, sampling strategies and diagnostic tests, which makes it difficult (and inappropriate) to combine and compare statistically. The authors refrain from statistical comparisons when the sampling methodology or geographic scale are different; however, the net result is the paper lacks cohesion. It is hard to see the "forest through the trees" with the density of information presented, much of which is not directly comparable. The authors' main thesis: that the prevalence is declining over time, likely due to deforestation, doesn't come through clearly from the way the data are presented (and the sheer length of the paper). I strongly encourage the authors to rethink how the informaton is presented and to remove any data that don't enable a comparison over time (e.g. any sites with single time point data) - or at least move this to a supplemental file. Focusing in on the few datasets and locations that tell the story will greatly strengthen this article.*

4. **Response**

We agree with the reviewer's central concern and have adopted the recommended solution in substance. All detailed single-time-point and design-heterogeneous datasets have been moved to S1 Appendix as Tables A to D. S1 Appendix Table A now also includes the health areas that returned non-endemic results in the 2003 mapping, so the appendix documents the boundary of the focus and not only its endemic core.

We have not removed the single-time-point observations altogether, because the manuscript addresses a second question alongside the temporal one, on where residual positive indicators occur, and how they correspond spatially with programmatic, entomological and landscape context. Removing all single-time-point observations would weaken the spatial evidence on which the recommendation to prioritise the Muda/Kuda and Lebu river basins rests. We have, however, made the distinction unambiguous. We label these observations as contextual throughout, do not pool them or compared them statistically, and report them with their sampling frames and limitations where applicable.

We hope the reviewer will find that the revised article now allows the central pattern to be read directly from the Abstract, the final Introduction paragraph, Table 2 and the first two paragraphs of the Discussion.

**Reviewer 2**

**Comment 1**

*This is a very well written paper where the authors combine multiple datasets to provide an assessment of changes in O. volvulus transmission in one district of DRC. Methods clearly described. Only remark, as most of the infection prevalence data is generally difficult to obtain, maybe good to know where the original village-level data was obtained from (besides the scoping review data).*

1. **Response**

We thank the reviewer for these constructive comments and agree that the provenance of the village-level data should be transparent. The four-skin-snip comparison derives from the moxidectin trial screening datasets. The individual-level records underlying the serological, nodule and two-skin-snip summaries, including previously unpublished and re-analysed records generated within the NSETHIO research programme, are supplied in full in S1 Dataset, and the source of every entry is identified there. In the main article, the column of Table 1 titled “Coverage sources (years: area [source of data])” cites the source of each dataset at health-area level, and S1 Appendix Tables A to D cite the source for every row. S1 Appendix Table A now also names the villages sampled in each nodule survey, so the provenance of those estimates can be traced from health area to village and source.

We should also explain why the article reports data at health-area rather than village level. Village names in this setting are fluid, as settlements are renamed, merged, split and are recorded with variant orthographies across surveys conducted two decades apart, so village names are not a reliable key for linking datasets. We therefore harmonised the records on GPS coordinates and on the official health-area and health-zone boundaries, and we present village-level detail spatially in the interactive map rather than in the text. As stated in the Methods and figure legends:

*“The map was produced in ArcGIS Pro (Esri, Redlands, CA, USA) using publicly available spatial datasets (GRID3 COD – Health Areas v5.0 [26] and GRID3 COD – Health Zones v5.0 [27]).”*

**Comment 2**

*Difficult to assess if the eligibility criteria for sampling of population for moxidectin clinical trial are comparable to the nodule prev and serological studies. Sample size and statistical analysis okay.*

2. **Response**

This is an important point, and on reflection we think our earlier framing was insufficiently precise. The sources differ materially in their sampling frames, and we have now stated this explicitly rather than implying broad comparability. Specifically, the revised manuscript distinguishes three categories:

- Community-recruited but age-restricted: the moxidectin trial screenings (2009–2011 and 2021–2023) enumerated all consenting residents aged ≥12 years in the participating villages before any trial treatment. These are population-based within their age range, and they form the main formal comparison because the age criterion and the four-snip protocol were identical between rounds.
- Community-based and all-age or child-based: the 2016 anti-Ov16 community survey in Logo (aged ≥3 years) and the unpublished community child surveys in 2018 (Logo) and 2021 (Nyarambe).
- Programmatic or purposive, and not population-representative: the REMO/REA nodule surveys, which by design examined a standardised sample of approximately 30–50 adults per site, predominantly men aged ≥20 years with ≥10 years of residence. Also, the onchocerciasis-associated epilepsy case-control and cohort studies, which sampled purposively.

The REMO/REA design is broadly standardised and therefore internally comparable across sites and over time, which is what makes it useful for delineating baseline endemicity, but it is not a community-representative sample, and we now say so directly in Table 1, in the Results and in the Limitations. Accordingly, the manuscript does not compare nodule or serological estimates statistically with the trial screening data. Each design is described in the Results and in the corresponding table, and only the two trial-screening rounds are compared with one another.

**Comment 3**

*This study is using adult blackfly collection data obtained during a study in 2017, where Human Landing Catches were performed with ethical concerns for the catchers.*

3. **Response**

We agree that the protection of collectors must be reported explicitly. These human landing catches were conducted and published previously, under approvals and safeguards described at the time. We have added the following to the Ethical statement of the revised manuscript, with the source cited:

*“The blackfly collections reported here were conducted under a previously published protocol [18] and approved by the ethics described above. Blackfly collectors were adults (≥18 years) from local communities who provided written informed consent, and were provided with ivermectin prior to participating in the collections.”*

We further note in Table 1 that human landing catches carry inherent ethical constraints, and the Recommendations propose that any future entomological monitoring in the focus employ standardised protocols with appropriate collector protection.

Reference 18: Post RJ, Laudisoit A, Mandro M, Lakwo T, Laemmer C, Pfarr K, Hoerauf A, Tortosa P, Gomard Y, Ukety T, Mande C, Farovitch L, Amazigo U, Bakajika D, Oguttu DW, Awaca N, Colebunders R. Identification of the onchocerciasis vector in the Kakoi-Koda focus of the Democratic Republic of Congo. PLoS Negl Trop Dis. 2022 Nov 4;16(11):e0010684.

**Comment 4**

*Sources of data described under Results section (lines 275-287 should rather be moved to Methods section)*

4. **Response**

We agree and have made this change. Source identification, inclusion and exclusion criteria, data extraction, provenance, harmonisation and the treatment of heterogeneous sources are now presented in Methods subsections 2.3 to 2.7. The Results retain only a brief overview of the assembled evidence base, that is, what the synthesis yielded before the specific findings are presented.

We agree. The source identification, inclusion and exclusion criteria, data extraction, provenance, harmonisation and treatment of heterogeneous sources are presented in Methods Sections 2.3–2.7. The Results retain only a concise overview of the evidence base before presenting specific findings, providing details obtained after conducting the synthesis.

**Comment 5**

*How were contradictory coverage data been dealt with (between CDTi cov data of different data sets and different age groups/target populations), as by Table 1 (should be explained in Methods section).*

5. **Response**

We deliberately did not reconcile the coverage estimates into a single value, because they refer to different populations, denominators and reporting platforms. ESPEN reports therapeutic coverage of the total population at health-zone level; the epilepsy case-control surveys report self-reported intake in the preceding round among the individuals sampled; and the 2021 child survey reports coverage among ivermectin-eligible children in a single health area. Averaging or reconciling these could create a biased single estimate. The Methods now state that coverage figures are reported separately, with their source, denominator and target population attached, and that discrepancies are described rather than resolved. The discrepancy in Kpanyi is retained explicitly as a limitation and a reason for independent coverage verification, as follows:

*“Lastly, self-reported ivermectin intake in Kpanyi (very low among the moxidectin trial participants in 2021–2023) appears inconsistent with high CDTI coverage reports available in ESPEN for Nyarambe (80–84%) [21] and with the 2021 coverage among ivermectin-eligible children in Kpanyi (92%). While possible explanations include social desirability or reporting bias during screening and different sampling frames, this discrepancy warrants a targeted coverage verification survey.”*

**Comment 6**

*As mentioned, due to the merging of various datasets with different study populations, some data are not representative of actual prevalence of infection. This also mentioned by the authors (lines 405-409), but how to deal with this to correct for the expected overestimation of seroprevalence?*

6. **Response**

We agree with the concern, and we have addressed it by restriction rather than by statistical correction. The non-representative serological samples come from case-control and cohort designs that oversampled persons with epilepsy. As the sampling fractions and the selection mechanism are not recoverable from the published data, any adjustment would rest on assumptions the designs cannot support and would risk conveying false precision. We therefore restricted the age- and sex-specific analysis to the 2016 community survey, did not pool the remaining serological datasets, moved their tables to S1 Appendix as Tables A to D, and interpreted them only as contextual evidence to indicate where transmission may still be occurring. This avoids presenting a biased pooled seroprevalence as a population estimate. The expected direction of the bias is now stated explicitly:

*“Firstly, several contributing studies were not designed to yield a population-representative sample of the focus. Most used pragmatic or study-specific enrolment criteria. For example, trial skin-snip screenings included participants aged ≥12 years, while case-control and epilepsy cohort studies used purposive sampling to investigate OAE rather than estimate population prevalence. Consequently, these studies may provide higher prevalence estimates than community-based surveys [55] and are interpreted as indicating where transmission may persist, rather than as estimates of population prevalence.”*

Reference 55: Siewe Fodjo JN, Mandro M, Mukendi D, Tepage F, Menon S, Nakato S, et al. Onchocerciasis-associated epilepsy in the Democratic Republic of Congo: Clinical description and relationship with microfilarial density. PLoS Negl Trop Dis. 2019;13(7):e0007300.

**Comment 7**

*Table 1, nodule prevalence surveys likely only performed among cases with epilepsy in the epilepsy cohort. If so, these nodule data are not representative for the full population. Difficult to assess as no reference to original epilepsy cohort dataset. If so, then the only representative dataset is the 2003 REMO dataset, which is already 20+ years outdated. How dealt with?*

7. **Response**

The reviewer is correct about the 2017 epilepsy cohort, which examined 38 adults living with epilepsy in Draju HA, is therefore not representative of the general population, and is identified as such in Table 1, in the Results and in S1 Appendix Table A, where no endemicity class is assigned to it.

Since submission we have been able to include a second nodule dataset from 2017 that is community-representative. In November 2017, an REA palpated 94 men aged ≥20 years in Draju, Kanga and Walla HAs (Logo HZ), following the standard REA convention of approximately 30 long-resident men per health area. Nodule prevalence was 44.4% (95% CI: 26.0–64.4) in Draju, 56.8% (95% CI: 39.7–72.5) in Kanga and 20.0% (95% CI: 8.4–39.1) in Walla. There is therefore now a standardised REA nodule series for Logo HZ spanning 2003, 2015 and 2017.

This prompted us to revisit the endemicity classification in S1 Appendix Table A. The REMO/REA thresholds were calibrated for pre-control settings. Logo HZ has never received CDTI, so its 2003, 2015 and 2017 surveys are all pre-control observations and are now assigned endemicity classes. Rethy HZ has been under CDTI since 2012, so its 2015 surveys measure residual burden under treatment and are left unclassified, as is the epilepsy cohort.

Three later epidemiological sources are also community-based, namely the 2016 anti-Ov16 serosurvey in Logo (n = 921 aged ≥3 years), the unpublished community child serosurveys of 2018 (Logo, n = 60 aged 3–10 years) and 2021 (Nyarambe, n = 95 aged 7–10 years), and the moxidectin microfilarial trial screenings (n = 8920 aged ≥12 years). The REMO surveys are used for what they were designed to do, that is, to establish pre-control endemicity and delineate the focus, and the temporal inference rests on the later community-based sources. We agree that a contemporary, representative, all-age survey of the focus is now needed, and this is the first of our Recommendations, as follows:

*“Operationally, and in line with the WHO 2030 onchocerciasis elimination goal [1], risk-based surveillance should prioritise the Muda/Kuda and Lebu River basins and adjacent communities to determine whether CDTI should be initiated in Logo HZ and continued in the neighbouring HZs.”*

**Comment 8**

*The description is complete for the various sections representing Tables 3-5, but as there are many variations across districts and diagnostics, a clear visualisation of these trends per district would be very much appreciated. Likewise, an easy visualisation (besides the online map) guiding the text concerning crab collections and Simulium breeding sites or species, (for instance with a stagged bar chart) would be welcome to guide the reader through the text.*

8. **Response**

We are sympathetic to the reviewer's wish for a visual guide, and we considered both suggestions carefully. Our concern is that a single trend figure combining the former Tables 3–5 would place on common axes estimates derived from different populations, eligibility criteria, diagnostic tests and skin-snip protocols. Readers would inevitably read the resulting lines as a trend, which is precisely the inference the data cannot linearly support and which Reviewer 3 also cautions against. Similarly, a stacked bar chart of crab and *Simulium* collections would imply comparability of abundance across surveys whose sampling effort, duration, season and site coverage were unfortunately not standardised.

We have therefore pursued the reviewer's objective by other means. First, the directly comparable four-snip trial-screening results are summarised in the main text and in Table 2. Second, the source-specific results are tabulated consistently in S1 Appendix Tables A to D. Third, the river-basin deforestation results are now summarised in a new Table 3 in the main text. Fourth and last, the interactive map allows simultaneous inspection of epidemiological, entomological, river, administrative and tree-cover layers by survey year and data type, which is the integrated visualisation the reviewer describes. The reason for not plotting these data together is now stated in the Methods, as follows:

*“Because study designs, diagnostics, age groups and protocols varied across data sources, temporal comparisons across heterogeneous contextual sources were descriptive, and formal analyses were limited to the repeated trial-screening comparison and the within-dataset analyses described below.”*

**Comment 9**

*The dense forest cover loss seems a stand-alone analysis, as no associative or causal analyses were performed between infection declines and forest losses. This is a lost chance. Also the spatial boundaries of the forest cover map has not been overlayed with infection prevalence. Fig 2 and Fig 4 are no separate images whereas it would be interesting to see where (over time) high infection rates would be found, having the forest cover loss directly underneath for clear comparison.*

9. **Response**

We understand the reviewer's point and share the wish that a formal association could be estimated. We did not attempt one because the epidemiological, entomological and remotely sensed observations differ fundamentally in timing, spatial support and sampling design. The infection data are point or health-area estimates from a handful of surveys, whereas the tree-cover data are annual 30-metre rasters. With so few epidemiological units observed repeatedly, and with treatment history confounded with geography, any regression of prevalence change on forest loss would be badly underpowered and could not distinguish ecological from programmatic effects. We concluded that reporting such a model would give the appearance of evidence that the design cannot deliver.

We have instead strengthened the descriptive integration in three ways. First, the interactive map provides exactly the overlay the reviewer requests, with the infection layers displayable over the annual tree-cover loss layer and the river network. Second, the Results now report the absolute area of dense forest present in 2000 and lost by 2024 for each river basin alongside the percentages, so that readers can see the scale on which these percentages are computed (e.g., the health-area figures are tabulated in S1 Appendix Table E, and the river-basin figures are now given as absolute areas alongside percentages in a new Table 3 in the main text). Third, we have added a limitation noting that 30-metre tree-cover data at a ≥70% canopy threshold will only partially capture the narrow riparian gallery forest most relevant to *Simulium neavei*, as follows:

*“Fourthly, tree-cover change was assessed from 30-metre imagery at a ≥70% canopy threshold. This measure captures the loss of forest blocks well, but only partially resolves the narrow riparian gallery forest most relevant to S. neavei.”*

**Comment 10**

*Figures/Tables/Images of succicient quality.*

10. **Response**

Thank you. The figures, tables and images have been retained at submission quality. Legends and placement have been revised where the restructuring required it, and all main figures are being uploaded separately in TIF format in accordance with the journal's figure guidelines.

**Comment 11**

*Yes (the conclusions/limitations are supported by the data presented), as mentioned a causal/associative relationship between canopy and forest cover with infection would help to substantiate these conclusions*

11. **Response**

We agree that such a relationship would substantiate the conclusions, and we regret that the available data do not permit it to be estimated credibly, for the reasons set out in response to Comment 9. We have therefore reframed the finding as descriptive spatial compatibility rather than association, added explicit limitations on timing, sampling and spatial resolution, and ensured that the Conclusion is proportionate to the evidence. The Recommendations identify the study design that would be needed to test the relationship directly.

**Comment 12**

*Lines 733-735, the authors refer several times to modelling studies, but why not compare your data with actual trends using field data? Think of publications from Burkina Faso, Ghana, Togo, Guinea-Bissau?.*

12. **Response**

We appreciate this point and have included a comparison with empirical field data in the Discussion. We would note, however, that the settings the reviewer mentions represent the converse situation to Kakoi–Koda, as they document persistent or recrudescent transmission despite prolonged control, whereas the present focus shows a decline under very limited control. Briefly, in Burkina Faso two biannual ivermectin rounds reduced microfilarial loads by 87–98% among microfilaria-positive participants in recrudescent villages [Reference 1]; in Ghana, focal transmission persisted under long-term ivermectin and historical vector control [Reference 2]; in Togo, long-term intervention reduced infection indicators in most surveyed areas while several river basins retained higher prevalence [Reference 3]; and Guinea-Bissau showed declining microfilarial indicators alongside residual anti-Ov16 and entomological signals [Reference 4].

The closest empirical analogue to our observations may be Uganda, where transmission declined independently of control following the disappearance of the vector, and we compare our findings with those foci, as follows:

*“In several Ugandan onchocerciasis foci, the disappearance of S. neavei has likewise been attributed to deforestation [Reference 5].”*

Reference 1: Nikièma AS, Koala L, Sondo AK, Post RJ, Paré AB, Kafando CM, Kambiré RS, Sow B, Bougouma C, Dabiré RK, Traoré S. The impact of ivermectin on onchocerciasis in villages co-endemic for lymphatic filariasis in an area of onchocerciasis recrudescence in Burkina Faso. PLoS Negl Trop Dis. 2021 Mar 1;15(3):e0009117.

Reference 2: Lamberton PH, Cheke RA, Winskill P, Tirados I, Walker M, Osei-Atweneboana MY, Biritwum NK, Tetteh-Kumah A, Boakye DA, Wilson MD, Post RJ, Basañez MG. Onchocerciasis transmission in Ghana: persistence under different control strategies and the role of the simuliid vectors. PLoS Negl Trop Dis. 2015 Apr 21;9(4):e0003688.

Reference 3: Vinkeles Melchers NVS, Agoro S, Togbey K, Padjoudoum K, Telou IG, Karabou P, Djatho T, Datagni M, Dorkenoo AM, Kassankogno Y, Bronzan R, Stolk WA. Impact of ivermectin and vector control on onchocerciasis transmission in Togo: Assessing the empirical evidence on trends in infection and entomological indicators. PLoS Negl Trop Dis. 2024 Jul 22;18(7):e0012312.

Reference 4: Vinkeles Melchers NVS, Manjuba C, Sodahlon Y, Correia RJ, Aiogalé VM, Assi LA, Cabral F, Cantey PT, Stolk WA. Onchocerciasis status in Guinea-Bissau: An overview of trends in epidemiological and entomological indicators. Int J Infect Dis. 2025 Oct;159:108035.

Reference 5: Katabarwa MN, Katamanywa J, Lakwo T, Habomugisha P, Byamukama E, Oguttu D, et al. The Imaramagambo Onchocerciasis Focus in Southwestern Uganda: Interruption of Transmission After Disappearance of the Vector Simulium neavei and Its Associated Freshwater Crabs. Am J Trop Med Hyg. 2016;95(2):417-25.

**Comment 13**

*yes, relevant study and strong that various ways of measuring transmission are taken into account. This study shows that areas of Muda/Kuda and Lebu River basins of DRC should be prioritised for onchocerciasis interventions (Kakoi-Koda focus). Interesting enough, it seems that – all data combined – the overall/global results are still not very different than the map presented in Fig. 2. In absence of an updated, and integrated map, this a actual lack in addressing the public health relevance.*

13. **Response**

We agree that the public-health interpretation should be made explicit, and we thank the reviewer for identifying the gap. The reviewer is right that the nodule picture has changed relatively little since Fig 2. In the November 2017 REA, nodule prevalence among men aged ≥20 years was still 44.4% in Draju and 56.8% in Kanga, lower but still within the hyperendemic range recorded there in 2003. The parasitological and child serological pictures, however, changed substantially over the same period. Microfilarial prevalence fell from 79.0% to 9.0% in Draju and from 68.9% to 8.6% in Kanga between the two trial screenings, and anti-Ov16 seroprevalence among children aged 3–10 years was 0–5% from 2016 onwards.

Adult worms survive for a decade or more and nodules persist with them, so nodule prevalence in long-resident adults records exposure accumulated before the decline, whereas microfilarial prevalence and child serology record current transmission. A nodule map of this focus will therefore continue to resemble the 2003 map for some years, which is itself programmatically relevant because it indicates which indicator should be used to verify whether transmission has been interrupted. The Discussion now states this:

*“For example, in Logo HZ, which never received CDTI, nodule prevalence in adult men remained in the meso- to hyperendemic range through 2017, whereas microfilarial prevalence in the same HAs (including the same villages) fell from 79.0% in 2010–2011 to 9.0% in 2021–2023. As adult worms survive for a decade or more and the men examined in 2017 had median ages of 28 to 46 years, persistent nodules are compatible with worms acquired before the decline.”*

We also accept the reviewer's underlying point about spatial extent. The residual positive signals we identified lie within, rather than beyond, the historically hyperendemic belt shown in Fig 2, and this is precisely why we recommend prioritising a circumscribed part of that area rather than the focus as a whole:

*“The concordance between very low-to-absent anti-Ov16 seroprevalence in children, decreasing adolescent and adult infection prevalence and intensity, and the spatial contraction of transmission suggested by the evidence to the Muda/Kuda–Lebu rivers corridor (where CDTI has not been delivered) supports prioritising this corridor for further investigation.”*

On the map itself, the interactive ArcGIS product integrates the infection indicators with rivers, vector observations, administrative boundaries and annual tree-cover loss, and is publicly accessible. Independent community interviews in Draju identified the same lowland valleys as the areas of greatest current biting nuisance, as follows:

*“Independent qualitative interviews with communities in Draju HA also identified the Kuda and Lebu lowland valleys as areas of highest current blackfly biting nuisance [31], independently highlighting these basins as priority areas for surveillance.”*

**Comment 14**

*Abstract “We collated anti-Ov16 serological data from epilepsy-related studies”, study population may be different (were OV16 samples collected.*

14. **Response**

We agree. The Abstract now identifies the population-based comparison first and labels the nodule, serological and two-snip sources unambiguously as contextual evidence derived from study-specific or purposive samples. The community, case-control and cohort serological datasets are kept separate throughout and are not pooled.

**Comment 15**

*Abstract, unclear aim of the study. (Yes, methods clear, but this should be better aligned with the abstract (missing an objective)).*

15. **Response**

We agree and have added an explicit objective to the Abstract, aligned with the final paragraph of the Introduction, as follows:

*“We explored whether this decline extended across the focus and how infection indicators corresponded spatially with ivermectin delivery, entomological observations and deforestation.”*

**Comment 16**

*Introduction: ref 2 doesn’t fit the quote (99% oncho in Africa, but ref is about GBD 2016 epilepsy).*

16. **Response:**

We are grateful to the reviewer for catching this error. The citation has been corrected to the Global Burden of Disease Study 2021 (published in 2024, now reference 2), whose supplementary appendix supports the statement that more than 99% of *O. volvulus* infections occur in sub-Saharan Africa.

**Reference**: 2.	GBD 2021 Diseases and Injuries Collaborators. Global incidence, prevalence, years lived with disability (YLDs), disability-adjusted life-years (DALYs), and healthy life expectancy (HALE) for 371 diseases and injuries in 204 countries and territories and 811 subnational locations, 1990-2021: a systematic analysis for the Global Burden of Disease Study 2021. Lancet. 2024;403(10440):2133-61.

**Comment 17**

*Ref 3 is not befit for quote in line 87, rather use studies on vector control from the OCP area. Lines 90-91, I think ref by Chesnais et al would be more appropriate : https://pubmed.ncbi.nlm.nih.gov/30268645/*

17. **Response**

We agree and have revised both citations. The statement on historical vector control is now supported by evidence from the OCP region (References 1 and 2 below). For the epilepsy statement, we have cited Colebunders *et al.* (2021), the Bradford Hill review (Reference 3), which synthesises the relevant evidence and includes both Chesnais *et al.* cohorts. We chose the review because the sentence makes a general epidemiological claim rather than reporting a specific cohort estimate.

Reference 1: Plaisier AP, van Oortmarssen GJ, Remme J, Habbema JD. The reproductive lifespan of Onchocerca volvulus in West African savanna. Acta Trop. 1991;48(4):271-84..

Reference 2: Karam M, Schulz-Key H, Remme J. Population dynamics of Onchocerca volvulus after 7 to 8 years of vector control in West Africa. Acta Trop. 1987;44(4):445-57.

Reference 3: Colebunders R, Njamnshi AK, Menon S, Newton CR, Hotterbeekx A, Preux PM, Hopkins A, Vaillant M, Siewe Fodjo JN. Onchocerca volvulus and epilepsy: A comprehensive review using the Bradford Hill criteria for causation. PLoS Negl Trop Dis. 2021 Jan 7;15(1):e0008965.

**Comment 18**

*- Line 117, CDTi for LF, this can only be ivermectin if Loa loa non-endemic, so LF Tx does not per se means it helps against O. volvulus. Explain more sharply*

18. **Response**

We agree and have revised the text. We now state that any relevance of the lymphatic-filariasis programme to *O. volvulus* arises specifically from the ivermectin component of the ivermectin-plus-albendazole regimen, and we reserve the term “CDTI” for onchocerciasis-directed distribution, using “mass drug administration” for the lymphatic-filariasis platform, for example:

*“Nyarambe later received mass drug administration of ivermectin and albendazole through the lymphatic-filariasis control programme [21].”*

We also would like to note that this regimen is consistent with the loiasis risk classification for the area, as the health zones of the Kakoi–Koda focus are mapped as low-risk for *Loa loa* endemicity in ESPEN (reference below), which is indeed why an ivermectin-containing regimen is used there.

ESPEN loiasis maps for the Democratic Republic of the Congo: [https://espen.afro.who.int/maps-data/diseases/loiasis?combine=&items_per_page =24&country_filter%5BCD%5D=CD#loaby-country--maps](https://espen.afro.who.int/maps-data/diseases/loiasis?combine=&items_per_page%20=24&country_filter%5BCD%5D=CD#loaby-country--maps)

**Comment 19**

*- S1 Database does not seem to have entomological data. There are some mistakes in the LAT/LON. Analyses actually only based on about 2000 records (age and sex stratified)?*

19. **Response**

We thank the reviewer for inspecting the dataset and for identifying these problems. Regarding coordinates, we have corrected and standardised all latitude and longitude fields to decimal degrees, corrected records in which the latitude and longitude fields had been interchanged, and set unavailable coordinates to NA. The corrected dataset and the mapped layers are now consistent.

Regarding entomological data, the reviewer is right that these were absent from S1 Dataset. The entomological observations had been extracted from published sources rather than being available as individual-level records for this synthesis. These are mentioned in the results and available in detail on the publication by Post *et al.* 2022 (Reference 1 below).

Regarding Record counts, S1 Dataset contains the individual human records drawn from the epidemiological source datasets that we were able to obtain at the individual level. Individual analyses used the relevant source-specific subsets rather than the whole file. In particular, the age- and sex-specific seroprevalence analysis used the 921 participants of the 2016 Logo community survey with valid serology, and the paired RDT–skin-snip comparison used the 83 children aged 3–10 years with both results available. The trial-screening data are of a larger order of magnitude (i.e., 8,920 residents screened in Logo and 1,083 in Nyarambe) and are reported at the aggregate level here because the individual-level analysis has been reported elsewhere (Reference 2 below). The aggregate figures are given in Table 2.

Reference 1: Post RJ, Laudisoit A, Mandro M, Lakwo T, Laemmer C, Pfarr K, Hoerauf A, Tortosa P, Gomard Y, Ukety T, Mande C, Farovitch L, Amazigo U, Bakajika D, Oguttu DW, Awaca N, Colebunders R. Identification of the onchocerciasis vector in the Kakoi-Koda focus of the Democratic Republic of Congo. PLoS Negl Trop Dis. 2022 Nov 4;16(11):e0010684.

Reference 2: Ngave FN, Wonyarossi DU, Abhafule GM, Mande JL, Nyathirombo A, Uvon CB, et al. Onchocerca volvulus infection prevalence and intensity in Logo and Nyarambe Health Zones in Ituri, Democratic Republic of the Congo in 2010 and in 2021-2023: results of screening for clinical trials of moxidectin versus ivermectin. Parasit Vectors. 2026;19(1).

**Reviewer 3**

**Comment 1**

*The key discovery is the transmission decline without CDTI, and the title does not highlight this. The authors should rephrase this title to make it more informative.*

1. **Response**

We agree that the decline, and the fact that it occurred in an area without routine onchocerciasis CDTI, should be visible in the title. We have not described the entire focus as untreated, because ivermectin delivery was heterogeneous. Angumu and Rethy implemented onchocerciasis CDTI; Nyarambe received ivermectin through lymphatic filariasis mass drug administration; small numbers of residents were treated within clinical trials; and Logo and Mahagi received no routine CDTI. The revised title therefore foregrounds both the decline and the limited extent of ivermectin delivery:

*“Declining Onchocerca volvulus transmission despite limited ivermectin delivery in the Kakoi–Koda focus, Ituri, Democratic Republic of the Congo: an epidemiological, entomological and landscape evidence synthesis”*

We have also harmonised the running title accordingly, as follows: *“Declining transmission despite limited ivermectin distribution”.*

**Comment 2**

*The background does not state the specific knowledge gap for Kakoi-Koda. Why do you conduct this study.*

2. **Response**

We agree and have rewritten the final paragraph of the Introduction to state why the decline observed in Logo required investigation, what remained unknown, how the present synthesis differs from the dedicated trial-screening article, and the three objectives:

*“The microfilarial prevalence decline observed in Logo, in the absence of CDTI, was unexpected and raised the question about how it related to the longer-term epidemiological history and wider spatial context of the Kakoi–Koda focus. In particular, the geographical extent of the decline, the location of possible residual transmission signals and their spatial correspondence with heterogeneous ivermectin delivery, entomological observations over time and landscape change remained to be elucidated. We therefore synthesised the available epidemiological, serological, programmatic, entomological and geospatial evidence in the last 25 years, including forest-cover change around streams, to describe changes in transmission indicators across the focus and to place the trial-screening findings within their broader eco-epidemiological context. While Ngave et al. (2026) describe the individual-level comparison of the two trial-screening periods [22], the present manuscript addresses the wider spatial and temporal evidence base. Our objectives were to identify temporal trends in infection indicators, characterise ivermectin delivery and entomological observations, and assess their descriptive spatial correspondence with tree-cover loss.”*

**Comment 3**

*Line 31: In the methodology, “we synthesized” is too vague. State clearly that this is a scoping review.*

3. **Response**

Revised accordingly, as follows:

“*We conducted a scoping evidence synthesis of onchocerciasis and Simulium vectors in the Kakoi-Koda focus from 1980 to 31 July 2025, informed by PRISMA-ScR guidance (PRISMA extension for scoping reviews, as detailed by Tricco et al. 2018 [29]).*”.

The search strategy, sources consulted, and screening and extraction procedures are set out in the Methods subsection 2.3.

**Comment 4**

*Line 45-46: The authors stated an overly confident causal statement: “Progressive deforestation…” change to “Progressive deforestation and canopy opening are consistent with a shift from crab-associated S. neavei to more open-habitat vectors, providing a plausible ecological mechanism.*

Line 62-64: "This decline appears linked to..." is again too causal. Change to: "This decline appears to be associated with..."

4. **Response**

We agree and have adopted the reviewer's wording in both places, and have carried the same correction through the Author Summary, Results, Discussion and Conclusion so that the language is consistent throughout. The Abstract now reads:

*“Extensive dense forest loss (75-90% in historically hyperendemic Logo) and canopy opening are consistent with a shift from crab-associated S. neavei habitats towards more open-habitat vectors, providing a plausible ecological mechanism.”*

and the Author Summary now reads:

*“This decline appears to be linked to the deforestation of the habitat suitable for some blackfly species, and to ivermectin distribution to treat onchocerciasis in parts of the focus.”*

**Comment 5**

*Line 96-103: The authors describe CDTI expansion in DRC, which is only tangentially relevant to the Kakoi-Koda focus. They should condense this part to one sentence.*

5. **Response**

We have condensed the passage substantially. It now runs to two sentences rather than one. The first gives the minimum programmatic context needed to explain why some areas of the focus were treated, and others were not, and the second notes the coverage constraints in the DRC. We retained the second sentence because heterogeneous and incompletely verified coverage is central to the interpretation of Kakoi–Koda and recurs in the Discussion and Limitations.

**Comment 6**

*Line 111-113 and line 118-126: The authors should move the detailed trial descriptions to a new subsection in methods (“Clinical trial contexts").*

6. **Response**

We agree and have created the subsection as suggested (Methods 2.4, “*Clinical trial contexts and repeated screening comparison*”). It distinguishes the trial-screening populations, the common four-snip protocol, the screening denominators, the enrolment criteria and the pre-treatment nature of the comparison, and it states that screening eligibility and treatment allocation are distinct. As follows:

*“Four trials delivered moxidectin or ivermectin to recruited participants in the focus: the Phase III moxidectin trial 3110A1-3000 (2010–2011 [33, 34]), a proof-of-concept ivermectin trial in persons with epilepsy (2017–2018 [35, 36]), and the Phase IIIb moxidectin trials MDGH-MOX-3002 (2021–2023 [22, 37]) and MDGH-MOX-3001 (2021–2026, ongoing, [22, 38]). The moxidectin trials recruited community residents aged ≥12 years and used four iliac-crest skin snips per person during screening [22, 33, 39, 40]. Screening eligibility and subsequent treatment allocation are distinct, that is, the prevalence denominator comprises all screened residents with valid microscopy, whereas enrolment required a specified infection threshold. The later trials expanded screening because few residents met the 2010–2011 trial criterion of ≥10 microfilariae per milligram of skin. Our repeated cross-sectional comparison is restricted to screening outcomes measured before each trial treatment. The individual-level, village-adjusted logistic regression comparing the two screening periods is reported in the dedicated 2026 article [22]. Therefore, the present synthesis reports HA-level estimates descriptively and does not duplicate that analysis.”*

**Comment 7**

*In the last paragraph the authors present a descriptive aim (“we synthesize… and relate”), not testable hypothesis. The authors should reframe this last paragraph and state specifics hypotheses.*

7. **Response**

We have reframed the paragraph and now state three explicit objectives, addressing temporal change in infection indicators, the spatial distribution of possible residual transmission signals, and their correspondence with programmatic, entomological and landscape evidence, as follows:

*“Our objectives were to identify temporal trends in infection indicators, characterise ivermectin delivery and entomological observations, and assess their descriptive spatial correspondence with tree-cover loss.”*

We have framed these as objectives rather than as formal hypotheses because the assembled evidence is observational, retrospective and heterogeneous, and a hypothesis-testing framing would imply an inferential design the sources cannot support. The corresponding hypothesis, that transmission in the Kakoi-Koda focus has been declining since at least the early 2000s under ecological rather than solely due to programmatic pressure, is stated in the Discussion together with the transmission-modelling study that would be required to test it quantitatively, as follows:

*“The concordance between very low-to-absent anti-Ov16 seroprevalence in children, decreasing adolescent and adult infection prevalence and intensity, and the spatial contraction of transmission suggested by the evidence to the Muda/Kuda–Lebu rivers corridor (where CDTI has not been delivered) supports prioritising this corridor for further investigation. Future transmission modelling could account for both programmatic exposure (including the targeted trial doses) and ecological change when attributing the observed declines.”*

**Comment 8**

*The authors should state in this section that most data sources are not population-representative and that results should be interpreted as descriptive of individuals sample, not necessarily in the general population. The authors should add a strong caveat like “due to the small number of skin-snip positive children, the sensitivity estimate is imprecise and should be interpreted with extreme caution.*

8. **Response**

We agree on both points. The revised Abstract and Methods distinguish the population-based comparison, baseline REMO and community serology from the purposive contextual datasets, which are not pooled, and the Limitations state that several contributing studies are not representative of the focus. For the diagnostic comparison, we used exact methods and have added the caveat the reviewer proposes, for example:

*“Skin-snip results were taken as a reference to estimate the sensitivity and specificity of anti-Ov16 RDT in the field with exact (Clopper-Pearson) 95% CIs, given the small sample size and sparse discordant data”.*

and

*“The sensitivity of skin-snip microscopy increases with the number of snips and may miss light infections (e.g., in low-transmission or settings under CDTI), as suggested by modelling work [60]. Similarly, the Ov16 IgG4 RDT performed on whole blood has low and setting-dependent sensitivity [57, 76]. To minimise bias, we restricted formal comparisons within study design groups, reported 95% CIs and ranges, and used exact tests (Fisher/Clopper-Pearson) when data were sparse.”*

**Comment 9**

*Standardized HLC protocols have existed for decades. "Informal spot-checks" are non-reproducible and highly biased. Entomologic data should be removed or heavily caveated. Furthermore, opportunistic collections with tiny sample sizes cannot support quantitative conclusions about vector species or infection rates..*

9. **Response**

We accept the substance of this criticism and have acted on it. We have withdrawn the pooled figure that combined the two compartments (previously reported as an overall prevalence of infection in head and/or body of 38.7%, 60/155), because heads and bodies are not independent observations from the same flies and the combined figure invited exactly the over-reading the reviewer warns against. We have retained the compartment-specific proportions, reported with their full numerators and denominators, because they describe the specimens actually examined rather than an estimate of a population parameter. We have made explicit, in the Methods, Results and Limitations, that the informal procedures do not permit estimation of the entomological indices that would matter programmatically, being biting rate, monthly or annual transmission potential, and the community infective rate, since these require standardised catch effort, duration, seasonality and site coverage, none of which the spot checks provide. The Limitations now state:

*“Thirdly, entomological findings are constrained by seasonal gaps, informal HLC procedures, limited PCR testing of S. vorax for O. volvulus larvae, and incomplete spatial coverage (notably across parts of Kakoi, Awo, Kuda/Muda and Lebu rivers), which limit the understanding of the capacity of these simuliid species to sustain O. volvulus transmission”.*

The Recommendations call for standardised entomological monitoring, with appropriate collector protection, before any programmatic use is made of entomological indicators in this focus, as follows:

*“Where ongoing transmission is verified, entomological monitoring should be considered to further characterise breeding sites, vector species, seasonal biting patterns, and infection/infectivity rates to guide interventions… In addition, blood-meal analysis could clarify host preferences of the current Simulium species found biting on humans and whether habitat change has altered human-vector contact, particularly in areas where flies may feed away from humans.”*

**Comment 10**

*Line 601: Why were only 4/23 S. vorax tested? This needs justification. The prevalence estimate (1/4 = 25%) is statistically meaningless.*

10. **Response**

Unfortunately, only four of the 23 *S. vorax* specimens collected were preserved and available for molecular testing. We agree that 1/4 cannot support a prevalence estimate, and we do not treat it as one. The figure is reported as a raw fraction so that readers can see how little it rests on, it is not compared with the *S. dentulosum* result, and no inference about *S. vorax* infection levels is drawn anywhere in the manuscript. Its only role is to record that *O. volvulus* DNA was detected in a species known to be anthropophagic in this focus. The Limitations state explicitly that these data cannot yield robust infection or infectivity rates:

*“Thirdly, entomological findings are constrained by seasonal gaps, informal HLC procedures, limited PCR testing of S. vorax for O. volvulus larvae, and incomplete spatial coverage (notably across parts of Kakoi, Awo, Kuda/Muda and Lebu rivers), which limit the understanding of the capacity of these simuliid species to sustain O. volvulus transmission. Nevertheless, S. dentulosum samples were confirmed to indicate infectivity (O. volvulus DNA in heads) within the focus [18], and S. vorax has demonstrated competence for O. volvulus in laboratory settings [65] and can transmit Onchocerca dukei in cattle [18, 77], indicating that both species remain epidemiologically relevant.”*

**Comment 11**

*Considering the different studies for skin snip in this article, two vs four skin snips is a major issue for comparing intensities. The authors mention this in Discussion, but it should be stated as a limitation in methods and handled more rigorously (e.g., only compare prevalences, not intensities, between the two protocols). The authors compare prevalence estimates between Table 4 (4 snips, mf/mg) and Table 5 (2 snips, mf/snip). This is invalid due to protocol heterogeneity.*

11. **Response**

We agree, and we have now ensured to compare neither intensities nor prevalences across the two protocols. The temporal comparison is restricted to the two trial-screening prevalence rounds, which used the same four-snip protocol and the same age criterion, against baseline REMO. The two-snip studies are retained as further contextual evidence, with their table moved to S1 Appendix (Table D), and no comparison of prevalence or of intensity is drawn between the two-snip and four-snip datasets. The reason is stated in the Methods and repeated in the Limitations, as follows:

*“Secondly, heterogeneity in study designs (trial screening, cross-sectional, case-control, cohort) and protocols (two versus four skin snips; microfilariae counted per snip or milligram of skin; differing age groups) limits precision and hampers direct comparisons of microfilarial intensity. The sensitivity of skin-snip microscopy increases with the number of snips and may miss light infections (e.g., in low-transmission or settings under CDTI), as suggested by modelling work [60]…”*

**Comment 12**

*Line 651-669: The paragraph with multiple percentages is indigestible. The authors should create supplementary table with all HA-level forest cover and loss percentages and summarize key patterns only in the text.*

12. **Response**

We agree and have done so. The complete health area-level data (i.e., dense forest present in 2000 and its cumulative loss, for all health areas of the five health zones) are now tabulated in S1 Appendix Table E, and the main text retains only the health zone summary and the key spatial patterns. The river basin results, previously given as a long parenthetical list, are now presented in a new Table 3 that reports the dense forest present in 2000 and the area lost in square kilometres alongside the percentage.

**Comment 13**

*Comparison of prevalence estimates between 2009-2011 and 2021-2023 by looking at whether confidence intervals overlap is a weak descriptive approach. A mixed-effects logistic regression with village as random effect and survey period as fixed effect would quantify the decline while accounting for clustering.*

13. **Response**

We agree that clustering by village and changes in participant composition require consideration. The individual-level comparison has recently been reported in the 2026 Parasites & Vectors article using logistic regression adjusted for age, sex, village and relevant interaction terms [Reference 1 below]. As the present manuscript is a scoping evidence synthesis, we now cite and summarise the published adjusted result rather than duplicate the individual-level analysis. As follows in the methods:

*“Our repeated cross-sectional comparison is restricted to screening outcomes measured before each trial treatment. The individual-level, village-adjusted logistic regression comparing the two screening periods is reported in the dedicated 2026 article [22]. Therefore, the present synthesis reports HA-level estimates descriptively and does not duplicate that analysis.”*

Reference 1: Ngave FN, Wonyarossi DU, Abhafule GM, Mande JL, Nyathirombo A, Uvon CB, et al. Onchocerca volvulus infection prevalence and intensity in Logo and Nyarambe Health Zones in Ituri, Democratic Republic of the Congo in 2010 and in 2021-2023: results of screening for clinical trials of moxidectin versus ivermectin. Parasit Vectors. 2026;19(1).

**Comment 14**

*Line 764-765: The hypothesis “sociopolitical instability and immigration of relatively wealthier households who are less engaged in…” is not supported by any data presented in the manuscript. The authors should remove it.*

14. **Response**

The explanation concerning sociopolitical instability, immigration and wealthier households has been removed.

**Comment 15**

*Line 781-785: "The marked onchocerciasis prevalence decline in Logo... points towards a substantial role for ecological drivers…" This is correlation, not causation. The authors do not have no historical entomological data on S. neavei abundance before deforestation. They should revise causal language.*

15. **Response**

We agree and have revised the statement, as follows:

*“The marked onchocerciasis prevalence decline in Logo despite the absence of routine CDTI is compatible with a potential contribution from ecological drivers, particularly canopy loss along streams, which could have reduced suitable breeding habitat for S. neavei and constrained transmission to short river segments where anthropophagic Simulium species remain. Their vector competence and ability to maintain transmission are uncertain”.*

**Comment 16**

*The authors claim the RDT is useful and supports its use. However, with 43% sensitivity, the RDT would miss more than half of truly infected children. The authors should revise this conclusion about anti-Ov16 RDTs.*

16. **Response**

We have adjusted the conclusion accordingly, as for *“cautious use as a complementary exploratory tool where the deployment of better diagnostics may not be feasible”.* Moreover, we no longer describe the sensitivity as “*moderate*”: the Discussion states that the field RDT showed low sensitivity…, and that this falls short of the WHO target product profile thresholds of ≥60% sensitivity for elimination mapping and ≥89% for stopping decisions, at ≥99.8% specificity.

**Comment 17**

*As part of the peer review process, I have reviewed the manuscript entitled "Epidemiological, vectorial and landscape changes in the context of declining Onchocerca volvulus transmission across the Kakoi-Koda focus, Ituri, Democratic Republic of the Congo" (Manuscript PNTD-D-26-00524). It is a synthesis work, bringing together epidemiological, entomological, programme-related and geospatial data from a historical onchocerciasis focus in the DRC over two decades. The triangulation of evidence and the public release of data are notable strengths.*

*However, the manuscript has several methodological and stylistic weaknesses that, in my opinion, prevent it from being accepted in its current form. I therefore recommend major revisions.*

17. **Response**

We thank the reviewer for recognising the value of integrating the epidemiological, entomological, programmatic and geospatial evidence, and for setting out so precisely where the manuscript was underdelivering. We have reorganised and shortened the manuscript, clarified the scoping-review methods and the hierarchy of evidence, created a dedicated Methods subsection on the clinical-trial contexts, moved heterogeneous detail to S1 Appendix, and substantially narrowed any potentially causal and diagnostic claims. The individual responses above identify each change.

**Journal requirements:**

*1) We noticed that you used the phrase 'unpublished data' in the manuscript. We do not allow these references, as the PLOS data access policy requires that all data be either published with the manuscript or made available in a publicly accessible database. Please amend the supplementary material to include the referenced data or remove the references.*

**Response**: The data previously described as unpublished are now supplied with the manuscript. The individual-level records from the 2018 and 2021 community child surveys generated within the NSETHIO project are included in S1 Dataset and summarised in S1 Appendix Table B, and the corresponding note now reads:

“C*ommunity survey data generated within the NSETHIO project [30] (see Methods [31]). Individual-level records are provided in S1 Dataset and summarised in S1 Appendix Table C*.”

*2) Please upload all main figures as separate Figure files in .tif or .eps format. For more information about how to convert and format your figure files please see our guidelines: https://journals.plos.org/plosntds/s/figures*

**Response**: We will submit the Figures separately.

*3) Some material included in your submission may be copyrighted.*

**Response:** All figures were created by the authors for this manuscript using the data sources cited in the figure legends. No previously published third-party figure has been reproduced. Required source and licence attributions have been included.

*4) Please amend your detailed Financial Disclosure statement.*

**Response:** amended.

We thank the Editor and the three Reviewers for the time and consideration they have devoted to this manuscript, and we hope the revised version is now suitable for publication in PLOS Neglected Tropical Diseases.

Yours sincerely,

Luís-Jorge Amaral, on behalf of all authors
