# Supplementary material for: Declining Onchocerca volvulus transmission despite limited ivermectin delivery in the Kakoi–Koda focus, Ituri, Democratic Republic of the Congo: An epidemiological, entomological and landscape evidence synthesis
Source: PLoS Negl Trop Dis. 2026 Aug 31;20(8):e0014164. doi: 10.1371/journal.pntd.0014164 (PMC13557497; doi:10.1371/journal.pntd.0014164)
Supplement: Peer review history

# Peer review: Decision Letter 0

**DOI:** 10.1371/journal.pntd.0014164.r001

**Authors:** Eva Clark, Robert Adamu SHEY

25 May 2026

PNTD-D-26-00524

Epidemiological, vectorial and landscape changes in the context of declining Onchocerca volvulus transmission across the Kakoi-Koda focus, Ituri, Democratic Republic of the Congo

PLOS Neglected Tropical Diseases

Dear Dr. Amaral,

Thank you for submitting your manuscript to PLOS Neglected Tropical Diseases. After careful consideration, we feel that it has merit but does not fully meet PLOS Neglected Tropical Diseases's publication criteria as it currently stands. Therefore, we invite you to submit a revised version of the manuscript that addresses the points raised during the review process.

Please submit your revised manuscript within by Jul 24 2026 11:59PM. If you will need more time than this to complete your revisions, please reply to this message or contact the journal office at plosntds@plos.org. When you're ready to submit your revision, log on to https://www.editorialmanager.com/pntd/ and select the 'Submissions Needing Revision' folder to locate your manuscript file.

Please include the following items when submitting your revised manuscript:

* A letter that responds to each point raised by the editor and reviewer(s). You should upload this letter as a separate file labeled 'Response to Reviewers'. This file does not need to include responses to any formatting updates and technical items listed in the 'Journal Requirements' section below.

* A marked-up copy of your manuscript that highlights changes made to the original version. You should upload this as a separate file labeled 'Revised Manuscript with Track Changes'.

* An unmarked version of your revised paper without tracked changes. You should upload this as a separate file labeled 'Manuscript'.

If you would like to make changes to your financial disclosure, competing interests statement, or data availability statement, please make these updates within the submission form at the time of resubmission. Guidelines for resubmitting your figure files are available below the reviewer comments at the end of this letter.

As the corresponding author, your ORCID iD is verified in the submission system and will appear in the published article. PLOS supports the use of ORCID, and we encourage all coauthors to register for an ORCID iD and use it as well. Please encourage your coauthors to verify their ORCID iD within the submission system before final acceptance, as unverified ORCID iDs will not appear in the published article. Only the individual author can complete the verification step; PLOS staff cannot verify ORCID iDs on behalf of authors.

We look forward to receiving your revised manuscript.

Kind regards,

Robert Adamu SHEY, Ph.D.

Guest Editor

PLOS Neglected Tropical Diseases

Eva Clark

Section Editor

PLOS Neglected Tropical Diseases

Shaden Kamhawi

co-Editor-in-Chief

PLOS Neglected Tropical Diseases

orcid.org/0000-0003-4304-636XX

Paul Brindley

co-Editor-in-Chief

PLOS Neglected Tropical Diseases

orcid.org/0000-0003-1765-0002

**Journal Requirements:**

1) We noticed that you used the phrase 'unpublished data' in the manuscript. We do not allow these references, as the PLOS data access policy requires that all data be either published with the manuscript or made available in a publicly accessible database. Please amend the supplementary material to include the referenced data or remove the references.

2) Please upload all main figures as separate Figure files in .tif or .eps format. For more information about how to convert and format your figure files please see our guidelines:

https://journals.plos.org/plosntds/s/figures

3) Some material included in your submission may be copyrighted. According to PLOS's copyright policy, authors who use figures or other material (e.g., graphics, clipart, maps) from another author or copyright holder must demonstrate or obtain permission to publish this material under the Creative Commons Attribution 4.0 International (CC BY 4.0) License used by PLOS journals. Please closely review the details of PLOS's copyright requirements here: <a data-cke-saved-href="https://journals.plos.org/plosntds/s/licenses-and-copyright" href="https://journals.plos.org/plosntds/s/licenses-and-copyright">PLOS Licenses and Copyright</a>. If you need to request permissions from a copyright holder, you may use the&nbsp;<a data-cke-saved-href="https://storage.googleapis.com/plos-published-prod/c4ff/Content%20Copyright%20Permission%20Form.pdf?X-Goog-Algorithm=GOOG4-RSA-SHA256&X-Goog-Credential=wombat-sa%40plos-prod.iam.gserviceaccount.com%2F20220817%2Fauto%2Fstorage%2Fgoog4_request&X-Goog-Date=20220817T210608Z&X-Goog-Expires=86400&X-Goog-SignedHeaders=host&X-Goog-Signature=cd9dc868773378f7033b9f8cfb91430167d6275df90bce01342a454f36512530b64f24736fbf68c4fd96da74d663b6ee95b3420bded8fb0e833af2390b896b8706643b476d7f532408921b60576d65b60617070900fd2bcd28eec4cd32fefcf007d3655df17fcc91023329a28479ce0d1c5758174eedcc9e3c1788c5f7dd8523f3ddd871e02c998a20f7dc78ec00f313d4b5a78ba2037a6c7ef0a05dc29817da9368065460c8d8446659f35f0458fefabd3101eb7f926e9cb7a0b53e7bbe47aaed3e0ba52f9af9c2b8f6d7d3a8871b205fc4cbe97384ed37c7604fd7eecfedccf49cba12820b2e3b649541cefde3103cd7d72731b3e8b0248439b4cba691ca8a" href="https://storage.googleapis.com/plos-published-prod/c4ff/Content%20Copyright%20Permission%20Form.pdf?X-Goog-Algorithm=GOOG4-RSA-SHA256&X-Goog-Credential=wombat-sa%40plos-prod.iam.gserviceaccount.com%2F20220817%2Fauto%2Fstorage%2Fgoog4_request&X-Goog-Date=20220817T210608Z&X-Goog-Expires=86400&X-Goog-SignedHeaders=host&X-Goog-Signature=cd9dc868773378f7033b9f8cfb91430167d6275df90bce01342a454f36512530b64f24736fbf68c4fd96da74d663b6ee95b3420bded8fb0e833af2390b896b8706643b476d7f532408921b60576d65b60617070900fd2bcd28eec4cd32fefcf007d3655df17fcc91023329a28479ce0d1c5758174eedcc9e3c1788c5f7dd8523f3ddd871e02c998a20f7dc78ec00f313d4b5a78ba2037a6c7ef0a05dc29817da9368065460c8d8446659f35f0458fefabd3101eb7f926e9cb7a0b53e7bbe47aaed3e0ba52f9af9c2b8f6d7d3a8871b205fc4cbe97384ed37c7604fd7eecfedccf49cba12820b2e3b649541cefde3103cd7d72731b3e8b0248439b4cba691ca8a">PLOS Content Copyright Permission&nbsp;Form</a>

Please respond directly to this email and provide any known details concerning your material's license terms and permissions required for reuse, even if you have not yet obtained copyright permissions or are unsure of your material's copyright compatibility. Once you have responded and addressed all other outstanding technical requirements, you may resubmit your manuscript within Editorial Manager.

Potential Copyright Issues:

i) Figures Figures 1, 2, 4, Appendix Figures B, C, D, E, F, G, and and H.. Please (a) provide a direct link to the base layer of the map (i.e., the country or region border shape) and ensure this is also included in the figure legend; and (b) provide a link to the terms of use / license information for the base layer image or shapefile. We cannot publish proprietary or copyrighted maps (e.g. Google Maps, Mapquest) and the terms of use for your map base layer must be compatible with our CC BY 4.0 license.

Note: if you created the map in a software program like R or ArcGIS, please locate and indicate the source of the basemap shapefile onto which data has been plotted.

If your map was obtained from a copyrighted source please amend the figure so that the base map used is from an openly available source. Alternatively, please provide explicit written permission from the copyright holder granting you the right to publish the material under our CC BY 4.0 license.

If you are unsure whether you can use a map or not, please do reach out and we will be able to help you. The following websites are good examples of where you can source open access or public domain maps:

* U.S. Geological Survey (USGS) - All maps are in the public domain. (http://www.usgs.gov)

* PlaniGlobe - All maps are published under a Creative Commons license so please cite u201cPlaniGlobe, http://www.planiglobe.com, CC BY 2.0u201d in the image credit after the caption. (http://www.planiglobe.com/?lang=enl)

* Natural Earth - All maps are public domain. (http://www.naturalearthdata.com/about/terms-of-use/).

4) Please amend your detailed Financial Disclosure statement. This is published with the article. It must therefore be completed in full sentences and contain the exact wording you wish to be published.

State what role the funders took in the study. If the funders had no role in your study, please state: "The funders had no role in study design, data collection and analysis, decision to publish, or preparation of the manuscript.".

If you did not receive any funding for this study, please simply state: u201cThe authors received no specific funding for this work.u201d

**Reviewers' Comments:**

Reviewer's Responses to Questions

**Key Review Criteria Required for Acceptance?**

As you describe the new analyses required for acceptance, please consider the following:

**Methods**

-Are the objectives of the study clearly articulated with a clear testable hypothesis stated?

-Is the study design appropriate to address the stated objectives?

-Is the population clearly described and appropriate for the hypothesis being tested?

-Is the sample size sufficient to ensure adequate power to address the hypothesis being tested?

-Were correct statistical analysis used to support conclusions?

-Are there concerns about ethical or regulatory requirements being met?

Reviewer #1: While the authors do a good job of avoiding inappropriate statistical comparisons, the net result is a very lengthy paper that is hard to read and extract the key findings. There aren't a lot of analyses in this paper, it is really a synthesis of existing data

Reviewer #2: This is a very well written paper where the authors combine multiple datasets to provide an assessment of changes in O. volvulus transmission in one district of DRC.

-Are the objectives of the study clearly articulated with a clear testable hypothesis stated?

Yes, clear, but this should be better aligned with the abstract (missing an objective).

-Is the study design appropriate to address the stated objectives?

Yes

Methods clearly described. Only remark, as most of the infection prevalence data is generally difficult to obtain, maybe good to know where the original village-level data was obtained from (besides the scoping review data).

-Is the population clearly described and appropriate for the hypothesis being tested?

Difficult to assess if the eligibility criteria for sampling of population for moxidectin clinical trial are comparable to the nodule prev and serological studies.

-Is the sample size sufficient to ensure adequate power to address the hypothesis being tested?

Yes

-Were correct statistical analysis used to support conclusions?

Yes

-Are there concerns about ethical or regulatory requirements being met?

This study is using adult blackfly collection data obtained during a study in 2017, where Human Landing Catches were performed with ethical concerns for the catchers.

Reviewer #3: Major revision:

The key discovery is the transmission decline without CDTI, and the title does not highlight this. The authors should rephrase this title to make it more informative.

The background does not state the specific knowledge gap for Kakoi-Koda. Why do you conduct this study.

Line 31: In the methodology, “we synthesized” is too vague. State clearly that this is a scoping review

Line 45-46: The authors stated an overly confident causal statement: “Progressive deforestation…” change to “Progressive deforestation and canopy opening are consistent with a shift from crab-associated S. neavei to more open-habitat vectors, providing a plausible ecological mechanism."

Line 62-64: "This decline appears linked to..." is again too causal. Change to: "This decline appears to be associated with..."

Line 111-113 and line 118-126: The authors should move the detailed trial descriptions to a new subsection in methods (“Clinical trial contexts").

Line 96-103: The authors describe CDTI expansion in DRC, which is only tangentially relevant to the Kakoi-Koda focus. They should condense this part to one sentence.

In the last paragraph the authors present a descriptive aim (“we synthesize… and relate”), not testable hypothesis. The authors should reframe this last paragraph and state specifics hypotheses.

The authors should state in this section that most data sources are not population-representative and that results should be interpreted as descriptive of individuals sample, not necessarily in the general population. The authors should add a strong caveat like “due to the small number of skin-snip positive children, the sensitivity estimate is imprecise and should be interpreted with extreme caution.

Standardized HLC protocols have existed for decades. "Informal spot-checks" are non-reproducible and highly biased. Entomologic data should be removed or heavily caveated. Furthermore, opportunistic collections with tiny sample sizes cannot support quantitative conclusions about vector species or infection rates.

Line 601: Why were only 4/23 S. vorax tested? This needs justification. The prevalence estimate (1/4 = 25%) is statistically meaningless.

Considering the different studies for skin snip in this article, two vs four skin snips is a major issue for comparing intensities. The authors mention this in Discussion, but it should be stated as a limitation in methods and handled more rigorously (e.g., only compare prevalences, not intensities, between the two protocols).

Line 651-669: The paragraph with multiple percentages is indigestible. The authors should create supplementary table with all HA-level forest cover and loss percentages and summarize key patterns only in the text.

Comparison of prevalence estimates between 2009-2011 and 2021-2023 by looking at whether confidence intervals overlap is a weak descriptive approach. A mixed-effects logistic regression with village as random effect and survey period as fixed effect would quantify the decline while accounting for clustering.

The authors compare prevalence estimates between Table 4 (4 snips, mf/mg) and Table 5 (2 snips, mf/snip). This is invalid due to protocol heterogeneity.

**********

**Results**

-Does the analysis presented match the analysis plan?

-Are the results clearly and completely presented?

-Are the figures (Tables, Images) of sufficient quality for clarity?

Reviewer #1: The ArcGIS interactive map is a useful feature and a nice product to come from this paper.

I don't find Table 3 very useful because of the drastically different sampling approachs (e.g. case-control vs. cross-sectional vs. epilepsy cohort) and differing sample sizes (ranging from 4 - 163).

Reviewer #2: -Does the analysis presented match the analysis plan?

Sources of data described under Results section (lines 275-287 should rather be moved to Methods section)

-Are the results clearly and completely presented?

How were contradictory coverage data been dealt with (between CDTi cov data of different data sets and different age groups/target populations), as by Table 1 (should be explained in Methods section).

As mentioned, due to the merging of various datasets with different study populations, some data are not representative of actual prevalence of infection. This also mentioned by the authors (lines 405-409), but how to deal with this to correct for the expected overestimation of seroprevalence?

- Table 1, nodule prevalence surveys likely only performed among cases with epilepsy in the epilepsy cohort. If so, these nodule data are not representative for the full population. Difficult to assess as no reference to original epilepsy cohort dataset. If so, then the only representative dataset is the 2003 REMO dataset, which is already 20+ years outdated. How dealt with?

The description is complete for the various sections representing Tables 3-5, but as there are many variations across districts and diagnostics, a clear visualisation of these trends per district would be very much appreciated.

Likewise, an easy visualisation (besides the online map) guiding the text concerning crab collections and Simulium breeding sites or species, (for instance with a stagged bar chart) would be welcome to guide the reader through the text.

The dense forest cover loss seems a stand-alone analysis, as no associative or causal analyses were performed between infection declines and forest losses. This is a lost chance. Also the spatial boundaries of the forest cover map has not been overlayed with infection prevalence. Fig 2 and Fig 4 are no separate images whereas it would be interesting to see where (over time) high infection rates would be found, having the forest cover loss directly underneath for clear comparison.

-Are the figures (Tables, Images) of sufficient quality for clarity?

yes

Reviewer #3: Minor revisions:

**********

**Conclusions**

-Are the conclusions supported by the data presented?

-Are the limitations of analysis clearly described?

-Do the authors discuss how these data can be helpful to advance our understanding of the topic under study?

-Is public health relevance addressed?

Reviewer #1: (No Response)

Reviewer #2: -Are the conclusions supported by the data presented?

Yes, as mentioned a causal/associative relationship between canopy and forest cover with infection would help to substantiate these conclusions

-Are the limitations of analysis clearly described?

Yes

-Do the authors discuss how these data can be helpful to advance our understanding of the topic under study?

Lines 733-735, the authors refer several times to modelling studies, but why not compare your data with actual trends using field data? Think of publications from Burkina Faso, Ghana, Togo, Guinea-Bissau?

-Is public health relevance addressed?

yes, relevant study and strong that various ways of measuring transmission are taken into account. This study shows that areas of Muda/Kuda and Lebu River basins of DRC should be prioritised for onchocerciasis interventions (Kakoi-Koda focus). Interesting enough, it seems that – all data combined – the overall/global results are still not very different than the map presented in Fig. 2. In absence of an updated, and integrated map, this a actual lack in addressing the public health relevance.

Reviewer #3: Major revision:

Line 764-765: The hypothesis “sociopolitical instability and immigration of relatively wealthier households who are less engaged in…” is not supported by any data presented in the manuscript. The authors should remove it.

Line 781-785: "The marked onchocerciasis prevalence decline in Logo... points towards a substantial role for ecological drivers…" This is correlation, not causation. The authors do not have no historical entomological data on S. neavei abundance before deforestation. They should revise causal language.

The authors claim the RDT is useful and supports its use. However, with 43% sensitivity, the RDT would miss more than half of truly infected children. The authors should revise this conclusion about anti-Ov16 RDTs.

**********

**Editorial and Data Presentation Modifications?**

Use this section for editorial suggestions as well as relatively minor modifications of existing data that would enhance clarity. If the only modifications needed are minor and/or editorial, you may wish to recommend “Minor Revision” or “Accept”.

Reviewer #1: The article is written very clearly - the prose is excellent - it is just too long and lacks cohesion.

Reviewer #2: In order to accept this manuscript, the authors will need to revise their manuscript. Recommendation is minor revision.

- Abstract “We collated anti-Ov16 serological data from epilepsy-related studies”, study population may be different (were OV16 samples collected

- Abstract, unclear aim of the study

- Introduction: ref 2 doesn’t fit the quote (99% oncho in Africa, but ref is about GBD 2016 epilepsy)

- Ref 3 is not befit for quote in line 87, rather use studies on vector control from the OCP area.

- Lines 90-91, I think ref by Chesnais et al would be more appropriate : https://pubmed.ncbi.nlm.nih.gov/30268645/

- Line 117, CDTi for LF, this can only be ivermectin if Loa loa non-endemic, so LF Tx does not per se means it helps against O. volvulus. Explain more sharply

- S1 Database does not seem to have entomological data. There are some mistakes in the LAT/LON. Analyses actually only based on about 2000 records (age and sex stratified)?

Reviewer #3: Minor revision

**********

**Summary and General Comments**

Use this section to provide overall comments, discuss strengths/weaknesses of the study, novelty, significance, general execution and scholarship. You may also include additional comments for the author, including concerns about dual publication, research ethics, or publication ethics. If requesting major revision, please articulate the new experiments that are needed.

Reviewer #1: This study seeks to synthesize data on onchocerciasis (both in humans and vectors) in the Kakoi-Koda focus in DRC. While such an effort is laudable and the authors clearly did a lot of work to compile all the information, it is difficult for the reader to digest as presented. The data include a mix of geographic locations, time points, sampling strategies and diagnostic tests, which makes it difficult (and inappropriate) to combine and compare statistically. The authors refrain from statistical comparisons when the sampling methodology or geographic scale are different; however, the net result is the paper lacks cohesion. It is hard to see the "forest through the trees" with the density of informaton presented, much of which is not directly comparable. The authors' main thesis: that the prevalence is declining over time, likely due to deforestation, doesn't come through clearly from the way the data are presented (and the sheer length of the paper). I strongly encourage the authors to rethink how the informaton is presented and to remove any data that don't enable a comparison over time (e.g. any sites with single time point data) - or at least move this to a supplemental file. Focusing in on the few datasets and locations that tell the story will greatly strengthen this article.

Reviewer #2: Overall, a nice and well-written manuscript with an in-depth analysis of one region in DRC. This is useful for advancing district-level onchocerciasis interventions. However, there is a strong recommendation to more clearly link forest cover and canopy data with infection. Now, these associations seem to be more of a speculation. Also, the authors use a lot of detailed tables, which are also informative, but I would strongly recommend to visualise the merged data – as visualations is more intuitive for many readers (the main figures are now the old REMO map, and the landcover map, which is not associated with infection occurrence.

Reviewer #3: As part of the peer review process, I have reviewed the manuscript entitled "Epidemiological, vectorial and landscape changes in the context of declining Onchocerca volvulus transmission across the Kakoi-Koda focus, Ituri, Democratic Republic of the Congo" (Manuscript PNTD-D-26-00524). It is a synthesis work, bringing together epidemiological, entomological, programme-related and geospatial data from a historical onchocerciasis focus in the DRC over two decades. The triangulation of evidence and the public release of data are notable strengths.

However, the manuscript has several methodological and stylistic weaknesses that, in my opinion, prevent it from being accepted in its current form. I therefore recommend major revisions.

**********

PLOS authors have the option to publish the peer review history of their article (what does this mean?). If published, this will include your full peer review and any attached files.

If you choose “no”, your identity will remain anonymous but your review may still be made public.

**Do you want your identity to be public for this peer review?**  For information about this choice, including consent withdrawal, please see our Privacy Policy.

Reviewer #1: No

Reviewer #2: No

Reviewer #3: No

[NOTE: If reviewer comments were submitted as an attachment file, they will be attached to this email and accessible via the submission site. Please log into your account, locate the manuscript record, and check for the action link "View Attachments". If this link does not appear, there are no attachment files.]

**Figure resubmission:**

**Reproducibility:**

To enhance the reproducibility of your results, we recommend that authors of applicable studies deposit laboratory protocols in protocols.io, where a protocol can be assigned its own identifier (DOI) such that it can be cited independently in the future. Additionally, PLOS ONE offers an option to publish peer-reviewed clinical study protocols. Read more information on sharing protocols at https://plos.org/protocols?utm_medium=editorial-email&utm_source=authorletters&utm_campaign=protocols

Attachment Submitted filename: Revision_Comment_Plos.docx

Attachment Submitted filename: Review report.docx

# Author comment: Author response to Decision Letter 1

**DOI:** 10.1371/journal.pntd.0014164.r002

4 Aug 2026

Attachment Submitted filename: IturiResponse to Reviewers.docx

# Editor report: Decision Letter 1

**DOI:** 10.1371/journal.pntd.0014164.r003

**Authors:** Eva Clark, Robert Adamu SHEY

17 Aug 2026

Dear Dr Amaral,

We are pleased to inform you that your manuscript 'Declining Onchocerca volvulus transmission despite limited ivermectin delivery in the Kakoi–Koda focus, Ituri, Democratic Republic of the Congo: an epidemiological, entomological and landscape evidence synthesis' has been provisionally accepted for publication in PLOS Neglected Tropical Diseases.

Before your manuscript can be formally accepted you will need to complete some formatting changes, which you will receive in a follow up email. A member of our team will be in touch with a set of requests.

Please note that your manuscript will not be scheduled for publication until you have made the required changes, so a swift response is appreciated.

IMPORTANT: The editorial review process is now complete. PLOS will only permit corrections to spelling, formatting or significant scientific errors from this point onwards. Requests for major changes, or any which affect the scientific understanding of your work, will cause delays to the publication date of your manuscript.

Should you, your institution's press office or the journal office choose to press release your paper, you will automatically be opted out of early publication. We ask that you notify us now if you or your institution is planning to press release the article. All press must be co-ordinated with PLOS.

Thank you again for supporting Open Access publishing; we are looking forward to publishing your work in PLOS Neglected Tropical Diseases.

Best regards,

Robert Adamu SHEY, Ph.D.

Guest Editor

PLOS Neglected Tropical Diseases

Eva Clark

Section Editor

PLOS Neglected Tropical Diseases

Shaden Kamhawi

co-Editor-in-Chief

PLOS Neglected Tropical Diseases

orcid.org/0000-0003-4304-636XX

Paul Brindley

co-Editor-in-Chief

PLOS Neglected Tropical Diseases

orcid.org/0000-0003-1765-0002

***********************************************************

p.p1 {margin: 0.0px 0.0px 0.0px 0.0px; line-height: 16.0px; font: 14.0px Arial; color: #323333; -webkit-text-stroke: #323333}span.s1 {font-kerning: none

# Editor report: Acceptance letter

**DOI:** 10.1371/journal.pntd.0014164.r004

**Authors:** Eva Clark, Robert Adamu SHEY

Dear Dr Amaral,

We are delighted to inform you that your manuscript, "Declining Onchocerca volvulus transmission despite limited ivermectin delivery in the Kakoi–Koda focus, Ituri, Democratic Republic of the Congo: an epidemiological, entomological and landscape evidence synthesis," has been formally accepted for publication in PLOS Neglected Tropical Diseases.

We have now passed your article onto the PLOS Production Department who will complete the rest of the publication process. All authors will receive a confirmation email upon publication.

The corresponding author will soon be receiving a typeset proof for review, to ensure errors have not been introduced during production. Please review the PDF proof of your manuscript carefully, as this is the last chance to correct any scientific or type-setting errors. Please note that major changes, or those which affect the scientific understanding of the work, will likely cause delays to the publication date of your manuscript. Note: Proofs for Front Matter articles (Editorial, Viewpoint, Symposium, Review, etc...) are generated on a different schedule and may not be made available as quickly.

Soon after your final files are uploaded, the early version of your manuscript will be published online unless you opted out of this process. The date of the early version will be your article's publication date. The final article will be published to the same URL, and all versions of the paper will be accessible to readers.

For Research Articles, you will receive an invoice from PLOS for your publication fee after your manuscript has reached the completed accept phase. If you receive an email requesting payment before acceptance or for any other service, this may be a phishing scheme. Learn how to identify phishing emails and protect your accounts at https://explore.plos.org/phishing.

Thank you again for supporting open-access publishing; we are looking forward to publishing your work in PLOS Neglected Tropical Diseases.

Best regards,

Shaden Kamhawi

co-Editor-in-Chief

PLOS Neglected Tropical Diseases

Paul Brindley

co-Editor-in-Chief

PLOS Neglected Tropical Diseases
